# Fluorescent PyrAte-(*S*)-citalopram conjugates enable imaging of the serotonin transporter in living tissue†

**DOI:** 10.1039/d4sc06949h

**Published:** 2025-03-03

**Authors:** Oliver J. V. Belleza, Iakovos Saridakis, Nadja K. Singer, Xavier Westergaard, Sergio Armentia Matheu, Miran Lemmerer, Margaux Riomet, Pedro A. Sánchez-Murcia, Nina Kastner, Stefanie Rukavina, Yi Xiao, Kathrin Jäntsch, Marco Niello, Klaus Schicker, David Sulzer, Leticia González, Nuno Maulide, Harald H. Sitte

**Affiliations:** a Centre of Physiology and Pharmacology, Institute of Pharmacology, Medical University of Vienna Währinger Straβe 13A 1090 Vienna Austria harald.sitte@meduniwien.ac.at; b Institute of Organic Chemistry, Faculty of Chemistry, University of Vienna Währinger Straβe 38 1090 Vienna Austria nuno.maulide@univie.ac.at; c Vienna Doctoral School in Chemistry (DoSChem), University of Vienna 1090 Vienna Austria; d Institute of Theoretical Chemistry, Faculty of Chemistry, University of Vienna Währinger Straβe 17 1090 Vienna Austria leticia.gonzalez@univie.ac.at; e Department of Psychiatry, Columbia University Irving Medical Center New York New York 10032 USA; f Department of Biological Sciences, Columbia University New York New York 10027 USA; g CeMM Research Center for Molecular Medicine of the Austrian Academy of Sciences Lazarettgasse 14 1090 Vienna Austria; h Division of Neurophysiology and pharmacology, Medical University of Vienna Währinger Straβe 13A 1090 Vienna Austria; i Departments of Psychiatry, Neurology and Pharmacology, Columbia University Irving Medical Center New York New York 10032 USA; j Division of Molecular Therapeutics, New York State Psychiatric Institute New York New York 10032 USA; k Research Platform NeGeMac Josef-Holaubek-Platz 2 (UZA II) 1090 Vienna Austria; l Hourani Center for Applied Scientific Research, Al-Ahliyya Amman University Amman Jordan; m Center for Addiction Research and Science – AddRess, Medical University Vienna Währinger Straβe 13A 1090 Vienna Austria

## Abstract

Fluorescent labeling techniques have enabled the visualization of various biomolecules, cellular structures, and their associated physiological processes. At the same time, there remains a demand for developing novel fluorescent compounds possessing unique chemical properties for biological imaging. A recently developed class of fluorophores, termed *PyrAtes*, displays optimal brightness and large Stokes shifts that are ideal for fluorescence microscopy. Herein, we report the development of PyrAte-based fluorescently labeled ligands that bind to the serotonin transporter (SERT), a membrane transport protein important for neurotransmitter homeostasis, which hitherto has not been visualized in its native environment using fluorescent small molecules. The design of a PyrAte fluorophore attached to (*S*)-citalopram, a selective serotonin reuptake inhibitor, resulted in the synthesis of two fluorescent drug conjugates varying in linker length: PYR-C6-CIT and PYR-C3-CIT. Docking and molecular dynamics experiments are performed to estimate their binding affinities to SERT. Our *in vitro* experiments confirm both compounds are effectively binding to SERT overexpressed in human embryonic kidney 293 cells, with the shorter conjugate displaying improved SERT affinity and membrane staining properties. Furthermore, *ex vivo* imaging of endogenous SERT was demonstrated in acute mouse brain slices using two-photon microscopy. The large Stokes shift of the PyrAte fluorophore enables simultaneous detection of its own fluorescence signal at 500 nm along with that of a yellow fluorescent protein-based serotonergic marker. Our findings provide novel tools for unprecedented SERT visualization and establish the utility of PyrAtes for the selective staining of membrane proteins in live cells and tissue.

## Abstract 2

We deploy a new class of fluorophores, PyrAtes, in the first-time use of small-molecule fluorophore–drug conjugates in imaging endogenous SERT *ex vivo*.

## Graphical abstract

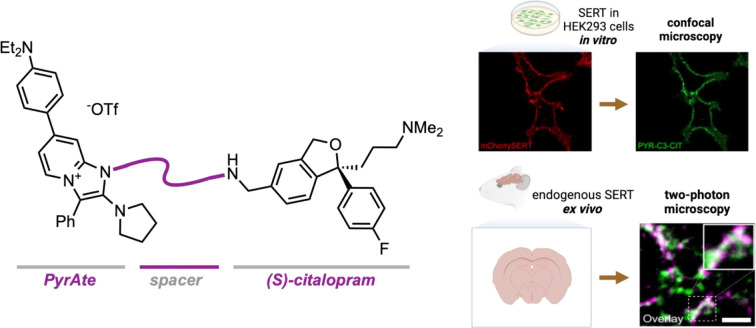

## Introduction

Fluorescence microscopy has become one of the most important and widely used techniques to investigate proteins and their associated biological processes. Advancements in microscopy, along with the aid of fluorescent labeling tools, have enabled the visualization of the expression and functional dynamics of ion channels, receptors, and transporters with remarkable spatial and temporal resolution.^1–5^ Traditionally, this has been done either through the use of fluorescent stains and fluorescently-labeled antibodies for fixed samples, or by genetically encoded fluorescent protein fusions for both live-cell and *in vivo* imaging.^1,2,4^ Other techniques include the use of protein tags which are covalently modified by reactive fluorescent probes, which allow simultaneous imaging of live biological samples and generation of fluorescent protein conjugates *in situ* (*e.g.*, HaloTag, SNAP-Tag).^6–9^ A similar approach is employed by fluorescently-labeled ligands or fluorophore–drug conjugates.^5,10,11^ These are functionalized, high-affinity protein-binding ligands chemically modified with a small-molecule fluorophore, which enable the observation of target proteins in their native state.

Fluorescent small-molecule dyes can possess certain advantages over fluorescent proteins in terms of enhanced brightness, photostability, and the ability to fine-tune their photophysical and chemical properties using organic synthesis.^4,12^ However, most available fluorescent ligands make use of the same core structures (*e.g.*, rhodamines or cyanines),^5,12,13^ which can limit their applications according to the spectral profiles, cell permeability, signal intensity, and synthetic routes that these chemical scaffolds would allow. Therefore, there is a need to continually develop novel fluorescent chemical structures to further expand the capacity of small-molecule dyes for biological imaging.

To address and fill this gap, we tapped into a recently reported family of fluorophores called *PyrAtes* to generate a class of novel fluorescent drug conjugates. The PyrAte fluorophores offer several advantages over other classes of compounds that are based on traditional fluorophores. The facile and highly modular synthesis of PyrAtes has been previously highlighted, enabling the fast buildup of a fluorophore library with a wide spectrum of emission at different wavelengths. The physicochemical properties relevant to their experimental use in organic solvents and aqueous buffers have also been reported.^14^ With their large Stokes shifts (typically >135 nm), PyrAtes offer versatility, allowing for combinations of various wavelengths of excitation and emission which could overcome some technical difficulties often encountered in microscopy. Large Stokes shifts also diminish the possibility of signal cross-talk and self-absorption, resulting in improved images with enhanced signal-to-background ratios.^15–18^

The serotonin transporter (SERT) is a transmembrane protein belonging to the solute carrier 6 (SLC6) family of proteins which also includes the dopamine transporter (DAT) and the norepinephrine transporter (NET). These neurotransmitter transporters (NTTs) are expressed in the central nervous system where their primary role is to participate in the clearance of monoamine neurotransmitter substrates (*e.g.*, serotonin, 5-HT, dopamine, DA, and norepinephrine, NE) from the synaptic cleft.^19,20^ Physiologically, this process is important in the regulation of neuromodulatory effects (*e.g.*, attention, motivation, learning) and neurotransmitter homeostasis. Dysregulation of these functions is related to common mental illnesses such as anxiety, depression, and attention-deficit hyperactivity disorder. Therefore, SERT, DAT, and NET are the therapeutic targets of several drugs which alleviate symptoms of these disorders, including the most widely prescribed, selective antidepressants.^21,22^ Moreover, these NTTs are the targets of commonly abused psychoactive substances such as cocaine and amphetamines.^23^

A recently published review provides an excellent overview of different fluorescent ligands that have been used to image the expression and function of monoamine NTTs.^24^ The use of a fluorescently-labeled drug conjugate to visualize NTTs is best exemplified by the rhodamine-based cocaine analogue JHC 01-64 in imaging DAT both in cellular models and primary cultures of neurons.^25–27^ Since the cocaine analogue exhibits non-selective affinity towards NTTs, it has also been used to investigate SERT oligomerization in live cells.^28^ Despite the fact that SERT is among the most widely studied NTTs,^29^ there remains a noticeable gap in the development of SERT-specific fluorophore–drug conjugates. For imaging SERT, quantum dot-based fluorescent ligands and antibodies have been used successfully in neuronal cells, although their method of application differs from organic fluorophores due to their larger size and the additional steps required in generating their conjugates *in situ*.^30,31^ Meanwhile, the selective serotonin reuptake inhibitors (SSRI), (*rac*)-citalopram and its (*S*)-enantiomer have been chemically tagged with rhodamine, resulting in the fluorescent compounds ZP 455 and VK2-83.^32,33^ These compounds have only been used, however, in the imaging of SERT overexpressed in human embryonic kidney (HEK293) cells. The utility of small-molecule fluorescent drug conjugates in imaging endogenously expressed SERT in neurons or tissue has so far not yet been described. For this reason, we chose to target SERT for our first report of a PyrAte-based fluorophore–drug conjugate.

The design, synthesis, and pharmacological characterization of PyrAte-based (*S*)-citalopram conjugates are herein described towards the live visualization of SERT. We demonstrate through computational and experimental methods the improvements in the design of fluorescent PyrAte conjugates. Using confocal microscopy, we show that PyrAte-(*S*)-citalopram conjugates specifically stain SERT-expressing HEK293 cells up to 60 minutes of continuous imaging and compound incubation. Finally, endogenously expressed SERT is visualized in acute mouse brain slices using two-photon microscopy. This work shows for the first time the successful use of novel PyrAte fluorophores in protein-targeted imaging, and more specifically that of native SERT in living brain tissue.

## Results and discussion

### The fluorescent PyrAte-C6-(*S*)-citalopram conjugate (PYR-C6-CIT) exhibits SERT activity and labels SERT expression in HEK293 cells

**Fig. 1:**
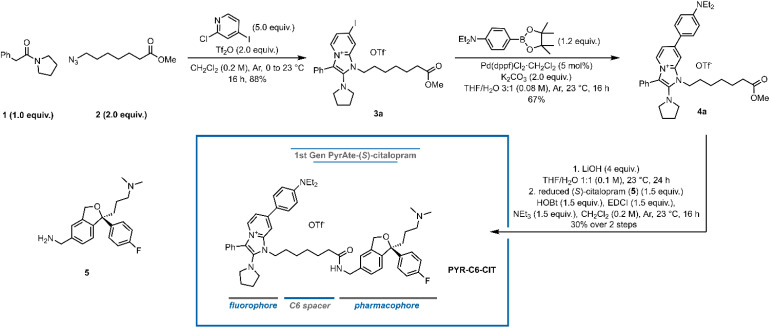
Synthesis of the first PyrAte-C6-(*S*)-citalopram conjugate PYR-C6-CIT. Equiv. = equivalents, Tf_2_O = trifluoromethanesulfonic anhydride, THF = tetrahydrofuran, dppf = 1,1′-bis(diphenylphosphino)ferrocene, HOBt = hydroxybenzotriazole, EDCI = 1-ethyl-3-(3-dimethylaminopropyl)carbodiimide. See ESI† for details.

In designing fluorescent drug conjugates, it is crucial to identify the optimal attachment point of the fluorophore to the parent drug where maximum ligand-target affinity can be retained. The inherent molecular simplicity of the particular pharmacophore bears the risk of dramatic activity loss by even subtle perturbation of its original structural features. In the case of citalopram, an affinity resin devised for the purification of SERT from blood platelets made use of the reduction of the nitrile group to an amine to form a conjugation site.^34^ The binding pose of (*S*)-citalopram at SERT also supports the idea that this nitrile moiety is amenable to covalent attachment to other larger moieties.^35,36^ Strømgaard and co-workers also demonstrated modifications on the *N*,*N*-dimethylamine are highly tolerated, while the group of Newman proved otherwise.^32,33,37^ In an analysis of rhodamine-based fluorescently labeled (*S*)-citalopram probes, they demonstrated that functionalization on the nitrile moiety of (*S*)-citalopram afforded 3-fold to 60-fold higher affinity to SERT compared to the respective amine-modified ligand. Inspired by this precedent, we designed the first PyrAte-C6-(*S*)-citalopram conjugate (PYR-C6-CIT, Fig. 1). Following the established protocol for the multicomponent synthesis of PyrAtes,^14^ benzylic carboxamide 1, azide 2, and 2-Cl-4-I-pyridine form the non-fluorescent iodo-Pyrate 3a. Suzuki–Miyaura cross-coupling on the chromophore core followed by carboxylic ester hydrolysis and amide coupling with the reduced (*S*)-citalopram 5 delivers the desired probe PYR-C6-CIT.

**Fig. 2:**
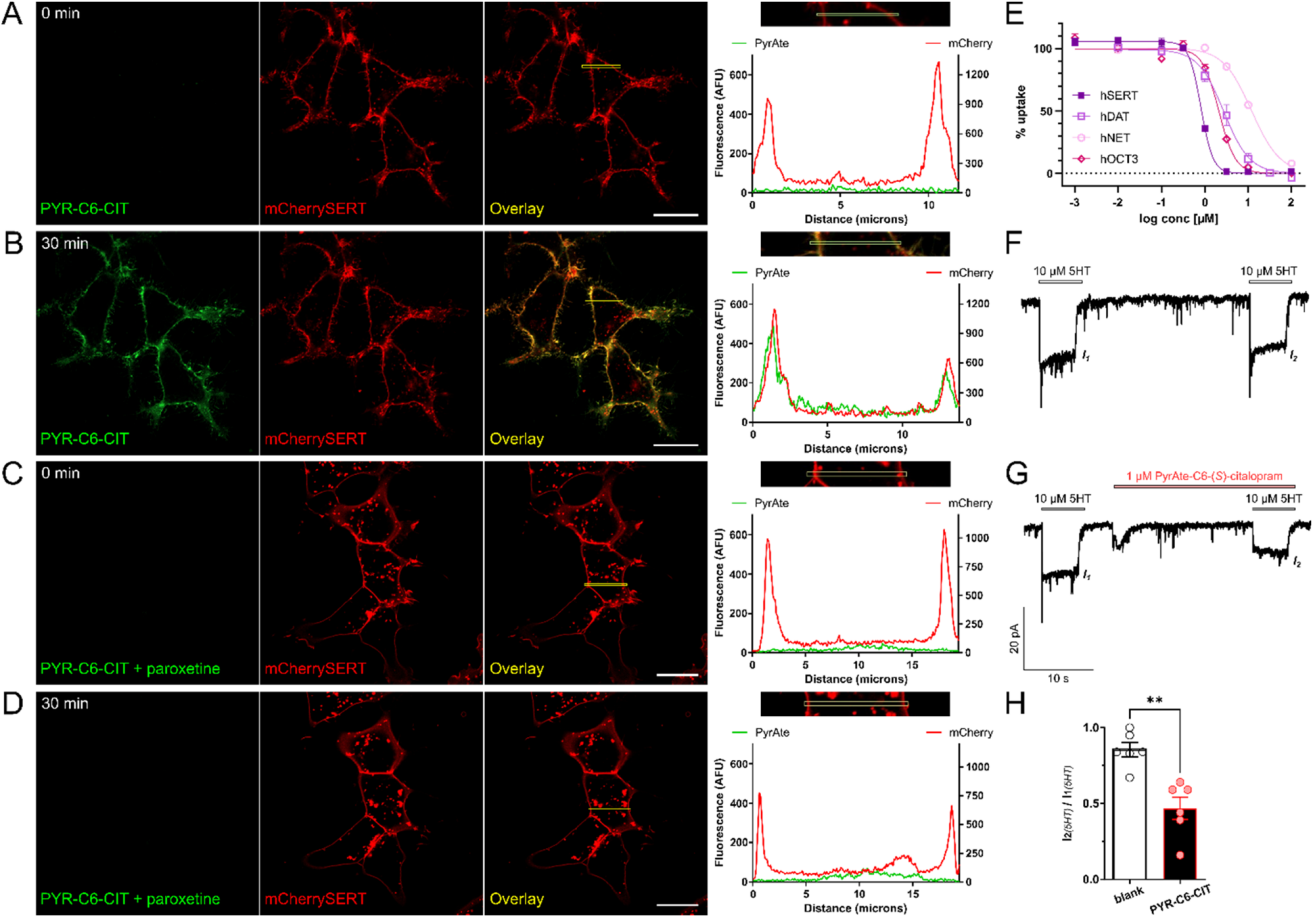
PyrAte-C6-(*S*)-citalopram (PYR-C6-CIT) is active in SERT as demonstrated in microscopy, radiotracer uptake inhibition, and electrophysiology experiments. SERT-specific membrane staining is displayed in confocal images: mCherry-SERT-expressing HEK293 cells imaged (A) prior to, and (B) after 30 min incubation with 20 nM PYR-C6-CIT; (C) after 10 min incubation with paroxetine; and (D) after 30 min incubation with 20 nM PYR-C6-CIT in the presence of paroxetine. Histograms show the pixel intensity profiles across the highlighted regions, showing co-localization of PyrAte and mCherry signals in (B), which is absent in other conditions (A, C and D), indicating SERT-specific membrane staining. PyrAte was imaged in the green channel (Ex. 405 nm, Em. 525/50 nm) and mCherry in the red channel (Ex. 561 nm, Em. 595/50 nm) using a confocal microscope. Representative images of at least three different experiments are shown. Scale bar = 20 μm. The observed binding is supported by inhibitory activity (E) at SERT (IC_50_ = 0.83 μM, Hill coefficient *n*_H_ = −3.1), along with accompanying activity in DAT (IC_50_ = 2.05 μM, *n*_H_ = −1.5), NET (IC_50_ = 12.6 μM, *n*_H_ = −1.3), and OCT3 (IC_50_ = 2.10 μM, *n*_H_ = −2.3). Data points display mean and SEM from *N* = 3–4, performed in triplicates. This was furthermore confirmed with electrophysiology: representative traces show (F) 5-HT-evoked currents measured in SERT-expressing HEK293 cells which are reduced (G) in the presence of 1 μM PYR-C6-CIT. (H) The calculated ratios of 5-HT-mediated currents before and after application of PYR-C6-CIT show a reduction to an average of 47% (*N* = 6). Data include individual points, mean, SEM, and **P < 0.01 as determined using two-tailed paired *t*-test.

HEK293 cells stably expressing mCherry-SERT were used to demonstrate the binding of PYR-C6-CIT to SERT. The red signals seen in Fig. 2A show that mCherry-SERT is localized predominantly in the membrane of these cells. Addition of 20 nM of PYR-C6-CIT increased the green signal co-localized at the membrane after 30 minutes of incubation (Fig. 2B), also shown as a vast difference in fluorescence signal in the histogram, indicating the high level of staining at the plasma membrane *versus* intracellular. This membrane staining was no longer observed when cells were pre-incubated with another SSRI, 30 μM paroxetine, 10 minutes prior to addition of PYR-C6-CIT (Fig. 2C and D). This confirms that the compound binds specifically to membrane-expressed SERT.

Interestingly, increasing the concentration to 100 nM resulted in signals localized in intracellular structures which were identified as mitochondrial staining (Fig. S1 and S2†). Such internalization became visible between 20 to 30 minutes after addition of the compound. This suggests that at higher concentrations and longer incubation times, PYR-C6-CIT is able to cross the cellular membrane and accumulate in intracellular organelles. It has been shown previously that the naked PyrAte dye enriches intracellularly in the mitochondria in a similar manner. This behavior can be attributed to the permanent positive charge of the pyridinium group along with the lipophilicity of the PyrAte molecule, which is presumably even enhanced by the addition of a six-carbon aliphatic chain linked to (*S*)-citalopram. Such phenomenon is not unique to the PyrAte fluorophores; it is also observed for rhodamine dyes and APP+, a fluorescent substrate of DAT and SERT also bearing a pyridinium functional group.^38–41^ Taken together, we have identified that a lower concentration (20 nM) and shorter incubation time (30 min) are the optimal conditions for the purpose of imaging plasma-membrane expressed SERT using PYR-C6-CIT.

The observed binding of PYR-C6-CIT at SERT is supported by data from uptake inhibition assays (Fig. 2E and Table S1†). It can be noted that PYR-C6-CIT retains the inhibitory activity of the parent drug (*S*)-citalopram. The measured IC_50_ value of 0.83 ± 0.11 μM (mean ± SD from *N* = 4, performed in triplicates) for PYR-C6-CIT, however, corresponds to a decrease of the compound's affinity by around 20-fold compared to (*S*)-citalopram. It is also noted how the selectivity to SERT *vs.* DAT is greatly affected by the PyrAtes. (*S*)-Citalopram, as an SSRI, exhibits greater than 1000-fold selectivity to SERT relative to DAT. In comparison, PYR-C6-CIT shows only 2.5-fold selectivity. When it comes to NET, PYR-C6-CIT also shows a reduction of selectivity compared to (*S*)-citalopram, going from around 300-fold down to 15-fold. This is unsurprising, as fluorophore–drug conjugates tend to lose some of their parent ligand's selectivity and affinity to their protein target.^5,24,25,32,33,42^ For example, AC1-146, a fluorescent ligand based on the selective NET inhibitor nisoxetine, has its NET affinity reduced by around 28-fold, and its selectivity towards SERT reduced to only about 18-fold compared to native nisoxetine.^42^ However, this reduction in selectivity is to a much greater extent compared to the rhodamine-based fluorescent (*S*)-citalopram analogue, VK2-83, which did not show any significant activity in DAT at 10 μM.^33^ This can be explained by the observed intrinsic affinity of the PyrAte fluorophore itself towards SERT, DAT, NET, which might have also contributed to differences in Hill coefficients (*n*_H_) observed. The unconjugated PyrAte-C6-methylester (PYR-C6-ME) exhibited IC_50_ values of 11.2 ± 0.4, 6.38 ± 0.5 μM, and 8.75 ± 2.14 μM at SERT, DAT, and NET, respectively (Table S1,† mean ± SD from *N* = 3–4, performed in triplicates). Despite its affinity towards SERT, however, PYR-C6-ME did not stain the membrane of mCherrySERT-expressing cells. Instead, it displayed rapid internalization (Fig. S1†). This suggests that the (*S*)-citalopram moiety of PYR-C6-CIT is the predominant driver of binding to SERT. On the other hand, since the PyrAte fluorophore bears a permanent positive charge in its pyridinium ring, it is possible that it could behave similarly to other cationic dyes (*e.g.*, ASP^+^) that are substrates or inhibitors of other proteins such as the organic cation transporters (OCT).^43–45^ Indeed, PYR-C6-ME displayed an affinity towards the organic cation transporter 3 (OCT3) with an IC_50_ value = 2.37 ± 0.46 μM, which is likewise observed in its conjugate with (*S*)-citalopram (Fig. 2E and Table S1†). This is in contrast to (*S*)-citalopram, which does not display affinity towards OCT3 on its own.^46^ A more extensive investigation of the pharmacology of naked PyrAte dyes could further improve our understanding of possible off-target effects.

To further confirm that the observed uptake inhibition is facilitated by the direct and inhibitory binding of the conjugate to the transporter, PYR-C6-CIT was also tested in membrane binding and electrophysiology experiments. Both PYR-C6-CIT and PYR-C6-ME also exhibited competitive binding with imipramine in SERT-containing membrane fractions with *K*_i_ = 0.11 ± 0.07 and 0.73 ± 0.25 μM, respectively (Fig. S3;† numbers represent mean ± SD of three experiments performed in duplicates). PYR-C6-CIT was able to inhibit 5-HT-mediated currents in SERT-expressing HEK293 cells by around 50% at 1 μM concentration, which further confirms the activity observed in the uptake inhibition assay (Fig. 2F–H).

### Optimizing the linker length: PyrAte-C3-(*S*)-citalopram (PYR-C3-CIT) improves on PYR-C6-CIT – both in SERT binding affinity and lipophilicity

**Fig. 3:**
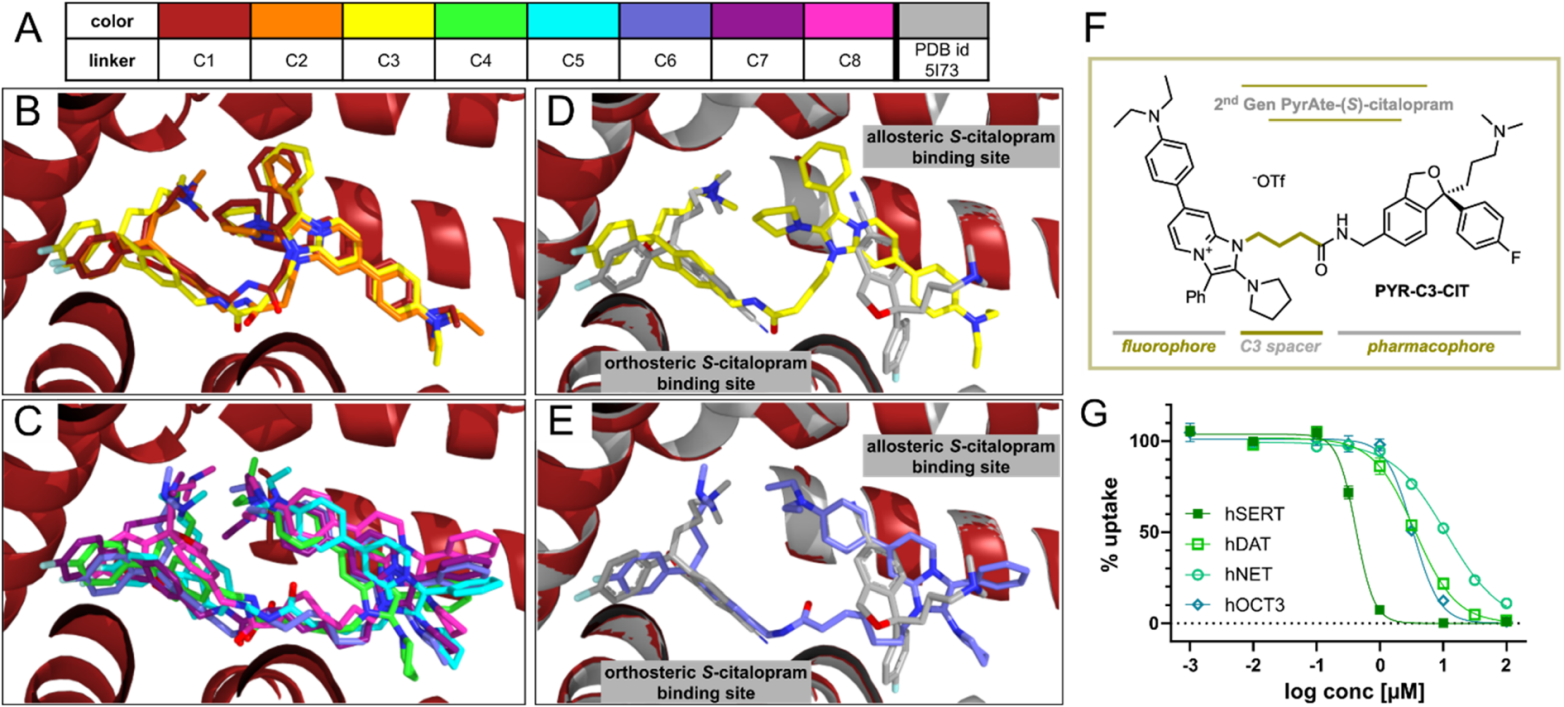
Varying the carbon spacer length results in a PyrAte-(*S*)-citalopram conjugate with improved activity at SERT and better selectivity with respect to DAT and NET. (A–E) Poses of PyrAte-(*S*)-citalopram ligands with variable linkers C1 to C8 (indicates the number of aliphatic carbon atoms in the spacer, color scheme shown in (A)) inside SERT (PDB. 5I73)^36^ obtained by docking and MD minimization. Linkers C1 to C3 are shown in (B), while C3 is shown again in (D) overlayed with the human SERT structure including two (*S*)-citalopram molecules in the orthosteric and allosteric binding sites. Linkers C4 to C8 are shown in (C), while C6 is shown again in (E) overlayed with SERT. (F) Structure of the second-generation PyrAte-C3-(*S*)-citalopram conjugate, PYR-C3-CIT. (G) Inhibitory activity of PYR-C3-CIT at SERT (IC_50_ = 0.40 μM, *n*_H_ = −3.0) is enhanced with better selectivity *vs.* DAT (IC_50_ = 3.57 μM, *n*_H_ = −1.3), NET (IC_50_ = 10.9 μM, *n*_H_ = −1.0), and OCT3 (IC_50_ = 3.32 μM, *n*_H_ = −2.1) in comparison to PYR-C6-CIT. Data points in the graph display mean and SEM from *N* = 3–4, performed in triplicates.

Aside from the fluorophore itself, the spacer or linker introduced between the fluorophore and the parent ligand can affect the physical and biological properties of the resulting conjugate as well as determine its practical utility.^5,47^ The ideal size of the linker depends on the nature of the fluorophore and how it can possibly interact within the binding site of the target protein.^47^ Along these lines, we sought to determine the optimal length of the carbon chain that links the PyrAte fluorophore moiety to the (*S*)-citalopram molecule. We hypothesized that a shorter carbon chain would reduce the overall lipophilicity of the resulting fluorescent conjugate, and therefore result in improved membrane staining. In order to test this hypothesis, we performed molecular docking, all-atom classical molecular dynamics (MD) simulations, free energy calculations, and quantum chemical lipophilicity studies. As an initial exploration, we docked different PyrAte-(*S*)-citalopram ligands with variable linker lengths (C1 to C8, with the number indicating the amount of aliphatic carbon atoms in the spacer) into the binding site of (*S*)-citalopram in the crystallographic structure of the human SERT (hSERT, PDB id. 5I73) (Fig. 3A).^36^ In all cases, the (*S*)-citalopram scaffold occupies the orthosteric (*S*)-citalopram binding site (also called S1-site), while the PyrAte fluorophore moiety resides in the allosteric binding site (the S2-site).^36,48–51^ However, we discovered some differences with the chain lengths. In the shorter linkers, with one up to three carbon atoms (C1 to C3, Fig. 3B), the pyrrolidine and phenyl moieties of the PyrAte scaffold are projected towards the (*S*)-citalopram part, whereas for larger linkers (C4 to C8, Fig. 3C) the PyrAte scaffold is rotated, pointing the diethylaniline moiety towards (*S*)-citalopram. For a clearer visual comprehension of the ligands' binding to hSERT, we present exemplary binding poses of C3 and of C6 superimposed with the two (*S*)-citalopram molecules in the orthosteric and allosteric binding sites of the crystallographic hSERT structure in Fig. 3D and E, respectively. Based on the observed binding poses and synthetic accessibility of the simulated derivatives, we selected the C3-derivative for chemical synthesis and more elaborate computational analysis. The synthesis of the 2nd generation PyrAte-C3-(*S*)-citalopram (PYR-C3-CIT) conjugate follows the same principles as the initial probe. Readily available materials undergo the multi-component reaction developed by us, with the exception of deploying the shorter methyl 4-azidobutanoate in place of azide 2 in Fig. 1. The photophysical properties of PYR-C6-CIT and PYR-C3-CIT are summarized in Table S5.†

To investigate whether PYR-C3-CIT provides a similar *in silico* binding affinity to hSERT, we performed 100 ns all-atom MD simulations of the two ligands in hSERT using the NAMD code.^52,53^ Additionally, we analyzed the hydrogen bonds that are involved in the binding of the ligands and estimated the binding free energies using the Molecular Mechanics/Poisson Boltzmann Surface Area (MM/PBSA) method as implemented in Amber.^54^ The MD simulations evidence an overall stable binding for PYR-C3-CIT and PYR-C6-CIT along the 100 ns trajectory (Fig. S7A and B†). The hydrogen bond analysis (Fig. S7C, D and Table S2†) reveals a comparable binding of PYR-C3-CIT and PYR-C6-CIT in terms of hydrogen bonds. Both PYR-C3-CIT and PYR-C6-CIT build their main hydrogen bond through the interaction of their linker-amide with T497 at an occupancy of around 50–60%. Although the conformation of the PyrAte scaffold is drastically different in the allosteric binding site of hSERT for PYR-C3-CIT and PYR-C6-CIT, we cannot disclose a major difference in hydrogen bond occupancy between the two poses. Moreover, the MM/PBSA results (Table S3†) indicate an equally favorable binding for PYR-C3-CIT and PYR-C6-CIT. All results evidence comparable binding strengths of PYR-C3-CIT and PYR-C6-CIT, making PYR-C3-CIT a viable alternative to PYR-C6-CIT.

To test whether the decrease in linker length leads to reduction in lipophilicity, the octanol/water partition coefficients (log *P*) were calculated quantum chemically for PYR-C3-CIT, PYR-C6-CIT, and also for comparison, for the unconjugated PYR-C6-ME. The calculations were performed with density functional theory at the B3LYP-D3BJ/def2-TZVP@SMD (water/*n*-octanol) level using the Gaussian 16 suite (Table S4†).^55–59^ The results nicely show a reduction of lipophilicity in terms of log *P* from 10.6 for PYR-C6-CIT to 8.4 for PYR-C3-CIT. Without the (*S*)-citalopram conjugate, the naked PYR-C6-ME dye has a log *P* value of 7.8.

**Fig. 4:**
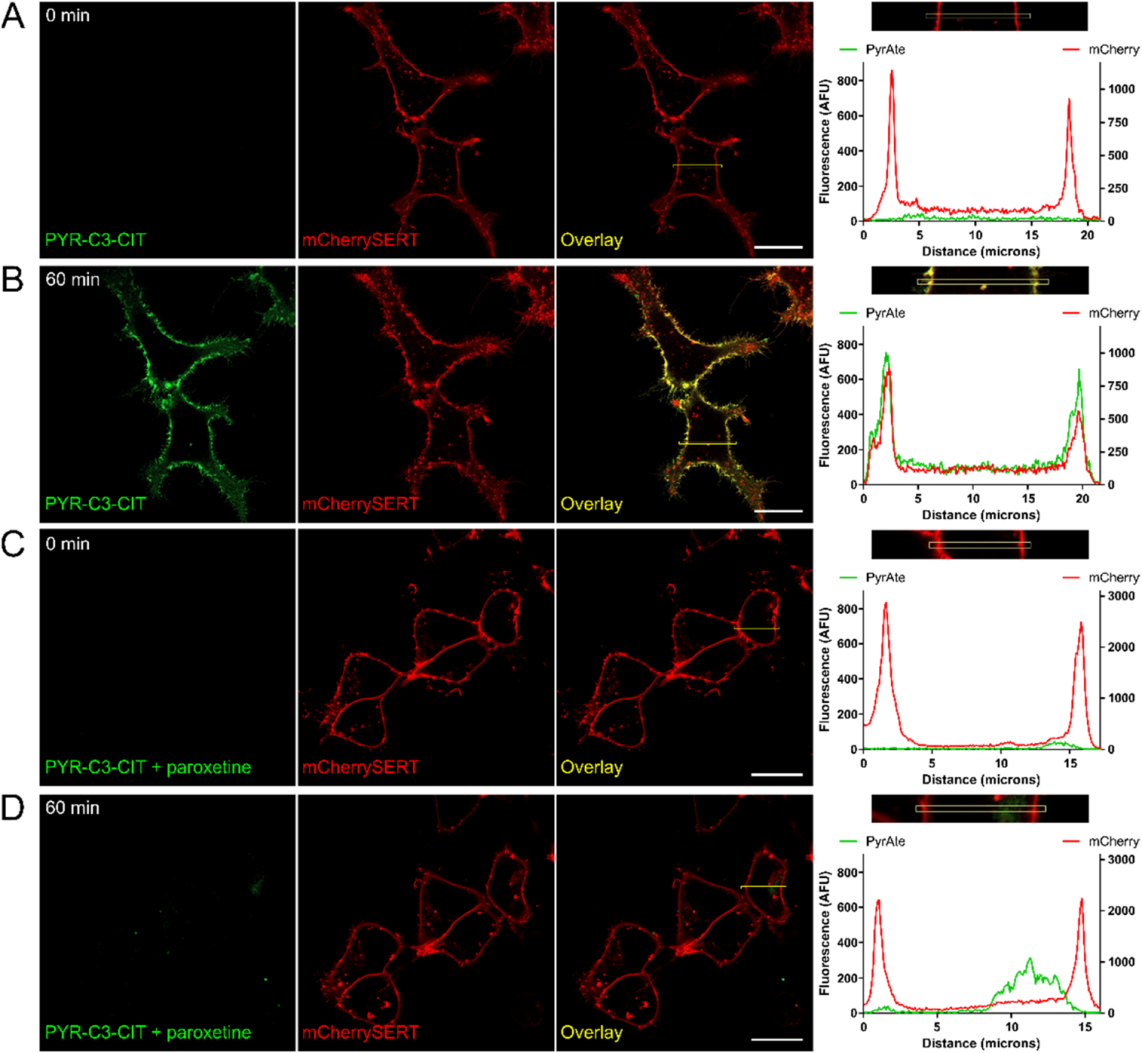
PyrAte-C3-(*S*)-citalopram (PYR-C3-CIT) exhibits SERT-specific membrane staining at a longer incubation period: mCherry-SERT-expressing HEK293 cells imaged (A) prior to, and (B) after 60 min incubation with 20 nM PYR-C3-CIT; (C) after 10 min incubation with paroxetine; and (D) after 60 min incubation with 20 nM PYR-C3-CIT in the presence of paroxetine. Histograms show the pixel intensity profiles across the highlighted regions, showing co-localization of PyrAte and mCherry signals in B, which is absent in other conditions (A, C and D), indicating SERT-specific membrane staining. A small degree of intracellular staining is observed in (D), which suggests SERT-independent internalization of compound. PyrAte was imaged in the green channel (Ex. 405 nm, Em. 525/50 nm) and mCherry in the red channel (Ex. 561 nm, Em. 595/50 nm) using a confocal microscope. Representative images of at least three different experiments are shown. Scale bar = 20 μm.

Importantly, experiments confirm that the change in the carbon linker length of PYR-C3-CIT resulted in improved imaging properties. As can be seen in Fig. 4, 20 nM of PYR-C3-CIT allowed a longer incubation period of 60 min with apparently enhanced co-localization of PyrAte and mCherrySERT signals and no internalization. Certainly, this is an improvement from the 30 minute incubation used for PYR-C6-CIT. Similar to what was previously observed with PYR-C6-CIT, the membrane staining is absent when co-incubated with paroxetine, confirming SERT-specific binding. A small degree of internalization was observed after 60 minutes in the presence of paroxetine, consistent with PyrAte-conjugate internalization by the cells being independent of the transporter. The improvement on SERT binding affinity is also seen from results of SERT uptake inhibition assays (Fig. 3G and Table S1†). The compound exhibited an IC_50_ value of 0.40 ± 0.05 μM (mean ± SD), which corresponds to a 10-fold difference compared to (*S*)-citalopram. The selectivity to SERT *vs.* DAT, NET, and OCT3 is also improved, showing around 10-fold selectivity against DAT, as opposed to the 2.5-fold selectivity observed with PYR-C6-CIT; 27-fold selectivity against NET compared to 15-fold; and lastly 8.2-fold selectivity against OCT3 instead of 2.5-fold. These results are in line with the previous knowledge that the difference in chain linker characteristics can greatly affect the binding properties of fluorescent drug conjugates.^5,10,47,60^ These also correlate well with the results of our computational experiments. The length of the linker influences the binding pose of PyrAte-(*S*)-citalopram conjugates in hSERT *in silico*, which translates to improvements in SERT inhibition potency and selectivity for PYR-C3-CIT. Moreover, the reduced log *P* calculated for PYR-C3-CIT supports the longer incubation period that is allowed for observing SERT-specific membrane staining without any significant internalization. A decrease in internalization also enhances the apparent signal at the membrane relative to background, which overall results in improved imaging of membrane-expressed SERT. Intracellular accumulation of fluorescent probes has been previously reported to mask the detection of specific binding at the membrane.^61,62^ In another study, a class of fluorescent cocaine analogues made use of various lengths of polyethylene glycol (PEG) linkers to overcome the lipophilicity of Janelia Fluor dyes and prevent cellular entry, which permitted their use in imaging live dopaminergic neurons.^63^

### PYR-C6-CIT labels SERT in acute mouse brain slices; PYR-C3-CIT co-localizes with yellow fluorescent protein (eYFP)-expressing serotonergic projections

**Fig. 5:**
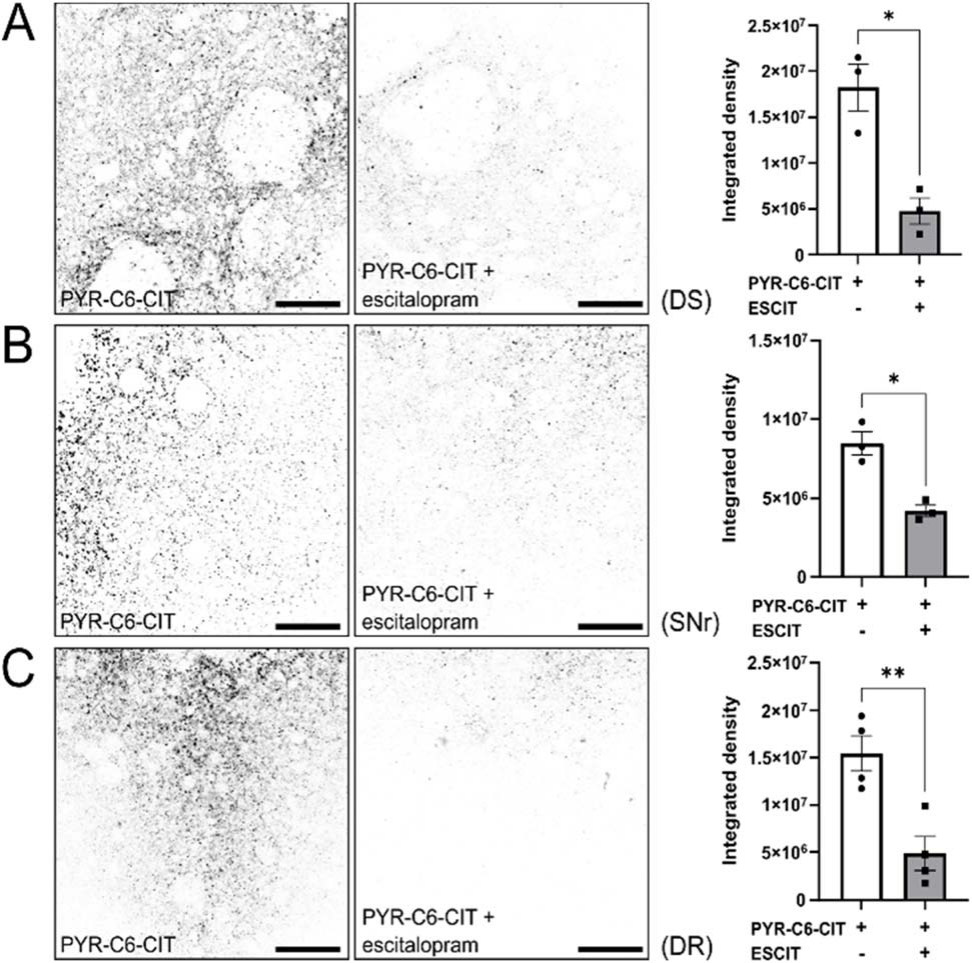
PyrAte-C6-(*S*)-citalopram (PYR-C6-CIT) exhibits SERT-specific staining in acute mouse brain slices at 500 nM concentration. Coronal mouse brain slices were prepared to observe staining in specific regions, namely the (A) dorsal striatum (DS), (B) substantia nigra reticulata (SNr), and (C) dorsal raphe (DR). Fluorescence staining by PYR-C6-CIT of SERT expression in axonal projections is shown as inverted images (non-inverted images in Fig. S4†). In the presence of escitalopram, the staining is significantly reduced as seen in integrated density analysis. PyrAte was imaged at 810 nm (Ex.) and 525/50 nm (Em.) using a two-photon microscope. Representative images were collected from 3–4 adult male C57/BL-6 mice. Scale bar = 50 μm. Data presented include individual values, mean, SEM, and *P < 0.05, **P < 0.01, as determined using Welch's unequal variances *t*-test.

To demonstrate the capability of PYR-C6-CIT to label endogenously expressed SERT, we used two-photon microscopy to observe different regions of the mouse brain where we expect SERT to be expressed.^64^ We examined the dorsal raphe (DR), the nucleus where diverse groups of mostly serotonergic neurons are concentrated, and the origin of serotonergic projections to the forebrain.^65,66^ We also examined the substantia nigra reticulata (SNr), a region in the midbrain where serotonergic projections co-enervate alongside dopaminergic neurons.^67^ Lastly, we examined the dorsal striatum (DS), an area that receives mostly dopaminergic but also serotonergic inputs. As seen in Fig. 5 and S4,†PYR-C6-CIT effectively stains all three areas examined: DR, SNr, and DS, as evidenced by bright, punctate signals. These are likely specific to SERT, since pre-incubation of the mouse brain slices with escitalopram (unconjugated (*S*)-citalopram) resulted in the reduction of these signals (Fig. 5).

**Fig. 6:**
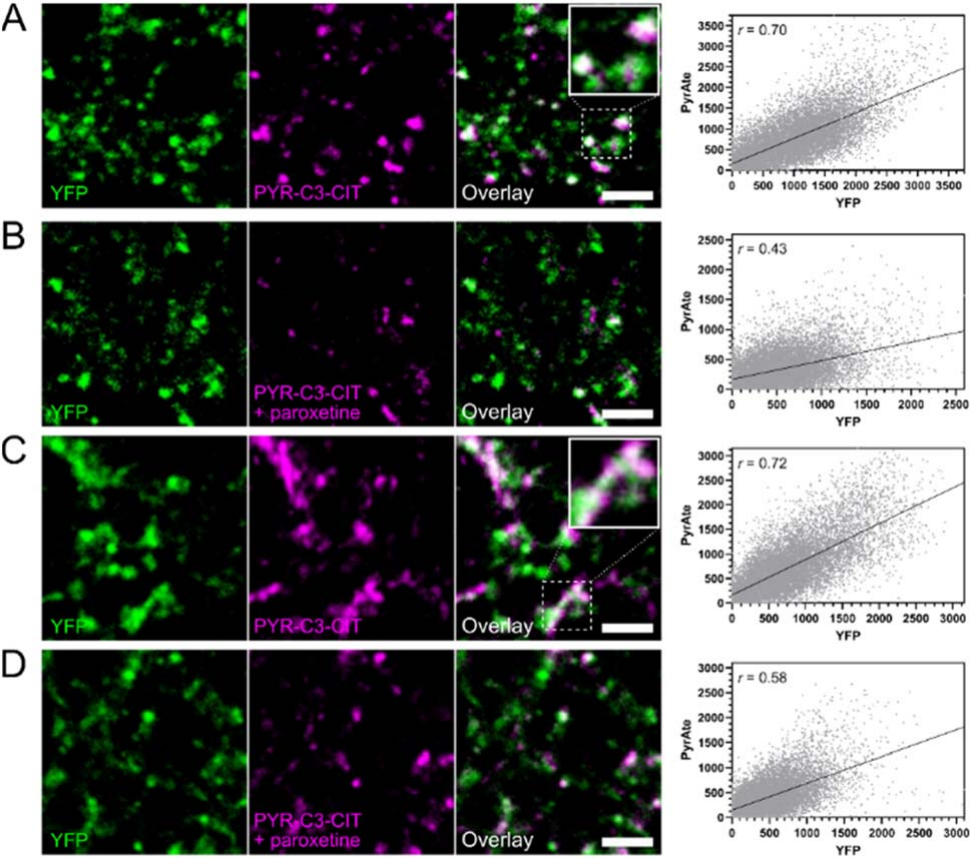
PyrAte-C3-(*S*)-citalopram (PYR-C3-CIT) co-localizes with eYFP-expressing serotonergic projections in the DS (A) and SNr (C), with Pearson's correlation coefficients, *r* = 0.70 and *r* = 0.72, respectively. In the presence of paroxetine, the amount of staining and overlap are reduced as seen in both the DS (B) and SNr (D), with Pearson's correlation coefficients, *r* = 0.43 and *r* = 0.58, respectively. Insets in solid squares show magnified images of areas within dashed squares. PyrAte was imaged at 810 nm (Ex.), eYFP was imaged at 965 nm (Ex.), and both detected at 525/50 nm (Em.). Representative images were collected from coronal brain slices of 3–4 adult Pet1-Cre/ChR2-eYFP mice. Scale bar = 5 μm.

PYR-C3-CIT also shows improved imaging properties in acute mouse brain slices. A lower laser power was needed to obtain images with similar signal-to-noise ratios seen using PYR-C6-CIT (Fig. S5†). To confirm the spatial distribution of SERT staining, we used transgenic Pet1-eYFP mice (Pet1-Cre/flox-ChR2-eYFP, see ESI†) expressing eYFP to visualize serotonergic neurons and axonal projections. Generation of this reporter line enables the Pet1-specific visualization of the serotonergic system.^68–70^ The overlap in PYR-C3-CIT and eYFP signals in brain slices obtained from Pet1-eYFP mice (Fig. 6) confirms the localization of the compound in serotonergic regions in the DS and SNr. This is further confirmed when the signals were reduced upon co-incubation with paroxetine, which is also reflected in the reduction in pixel values and Pearson's correlation coefficients seen in the fluorescence intensity graphs. These signals arising from both PYR-C6-CIT and PYR-C3-CIT, however, were not completely abolished when SERT was blocked by either inhibitor: escitalopram or paroxetine. As is the case for most fluorescent stains, non-specific background staining remains to some extent, which could be arising from both off-target effects of the dye and autofluorescence in the tissues. OCT3 is widely distributed and plays a significant role in the clearance of monoamine neurotransmitters in the brain.^71^ Interestingly, the decrease in fluorescent signals in the presence of SERT inhibitors is least pronounced in the SNr, part of a dopaminergic region in the midbrain, which could reflect the weakness of the compounds in terms of SERT/DAT selectivity. The combined results from PYR-C6-CIT and PYR-C3-CIT nevertheless show the extensive distribution of SERT in the mouse brain. It has been previously established using immunohistochemistry, autoradiography, and voltammetry studies that SERT is distributed in the DR, the SNr, and the caudate putamen (which includes the DS) at varying densities.^64,67,72–74^ The punctate staining pattern observed with the PyrAtes also reflects the distribution of SERT into axonal terminals and serotonergic boutons. This is consistent with SERT functionality, as it is also seen in other fluorescence imaging studies that describe SERT expression in the brain.^70,72,75,76^

It is also worth noting that this is the first instance we have shown that the PyrAte fluorophores are compatible with a two-photon imaging system. The PyrAte fluorophore emits in the green region of light at around 500 nm which is typically detected using a 525/50 nm bandpass filter in most imaging applications. Due to its large Stokes shift, its wavelengths of excitation lie within the blue region. Using two-photon excitation microscopy, the PyrAte was found to be excitable at 810 nm which corresponds to twice the excitation wavelength normally used in single-photon microscopy (405 nm). Another green-emitting fluorophore, eYFP, is not excited at this wavelength; its maximum two-photon excitation is found at 960 nm.^77^ This enabled us to simultaneously detect both PyrAte and eYFP signals in a single emission channel using two different excitation lasers, with no apparent signal cross-over (Fig. S6†). Such lack of overlap in spectral profiles is particularly useful in obtaining multi-channel images in combination with other fluorophores. In recent years, two-photon microscopy has been applied in the detection of serotonergic activity in living brain tissues and also *in vivo* using genetically encoded sensors based on fluorescent protein fusions of 5-HT receptors.^78–81^ For example, GRAB_5-HT_ is a selective fluorescent sensor for 5-HT based on the 5-HT_2C_ receptor and cpGFP which has been shown to detect changes in extracellular 5-HT concentrations in the prefrontal cortex of mice in response to a psychostimulant, methylenedioxymethamphetamine (MDMA).^80^ A recently reported fluorescent probe based on a SERT substrate demonstrates a SERT-dependent intracellular accumulation and labeling of serotonergic neuronal cell bodies and axonal projections in the mouse brain that is orthogonal to the fluorescent sensing function of GRAB_5-HT_.^82^ Since SERT plays an important role in regulating 5-HT concentrations, we envision a possibility of co-imaging 5-HT concentration fluxes along with SERT expression and activity *ex vivo*. Our images demonstrate the capacity for live imaging of axonal SERT expression in their native state, *i.e.*, without the need for creating genetically modified constructs and without fixation or permeabilization, which can be both expensive and time-consuming. This enables a clearer visualization of SERT with temporal control, and the possibility of real-time monitoring of changes in response to different treatment conditions.

In summary, we have developed PYR-C6-CIT and PYR-C3-CIT, two fluorescent (*S*)-citalopram analogues based on a novel family of fluorophores, a first-time use of fluorescent drug conjugates in labeling endogenously expressed SERT in acute mouse brain slices. An interesting caveat is presented by the intrinsic pharmacological profile of the PyrAte fluorophore, which can possibly interact with the allosteric binding site at SERT, but may also confer off-target binding affinities on its fluorophore–drug conjugates. Both SERT-targeting fluorescent probes previously reported based on citalopram and (*S*)-citalopram, ZP 455 and VK2-48, display the extraordinary selectivity towards SERT *versus* DAT that is expected of SSRIs.^32,33^ In this regard, both compounds should be better suited for selectively imaging SERT in live neurons or brain tissues where a heterogeneous population of NTTs is expressed. However, it has been reported that despite its remarkable selectivity, ZP 455 was not optimal for imaging live neurons.^24,32^ To our knowledge, VK2-48 has only been successfully used in cultured cell models, and not for endogenous SERT.^33^ The enhanced optical properties provided by the PyrAte fluorophore could compensate for the decrease in affinity of its conjugates towards SERT, therefore enabling the SERT-specific imaging demonstrated by PYR-C6-CIT and PYR-C3-CIT. Such display of SERT visualization in both live cultured cells and brain tissues shows great promise in advancing the practical utility of fluorophore–drug conjugates. However, the loss of selectivity and resulting off-target effects can complicate their practical use. In order to effectively isolate SERT-specific signals, non-specific staining could be reduced by co-application with other pharmacological blockers (*e.g.*, DAT, NET, and OCT3 inhibitors).

This study also provides insights into the effective design of PyrAte fluorophores so that their chemical properties can be improved, especially in the context of biological applications. We have shown that changing the linker length results in a reduction of lipophilicity, along with consequent improvements in affinity, selectivity, and membrane staining properties. This could be further improved by adding hydrophilic groups such as charged sulfonate or carboxylate groups into the core structure of PyrAtes. Several classical fluorophores, such as rhodamines and cyanines are made water-soluble by their sulfonated analogues.^12^ Hydrophilic fluorophores are more desirable not only due to their ease of handling in biological buffers (*i.e.*, lessening the need for organic co-solvents), but also because lipophilic fluorophores can cause undesirable non-specific staining which severely limits their application in complex biological systems. For example, the Oregon Green-based cocaine analogue MFZ 9-18 is more hydrophilic compared to the rhodamine-based JHC 1-64.^26^ The latter has been used to image expressed DAT and NET in live neuronal and non-neuronal cells,^26,27,83–85^ while the former was successfully utilized in imaging dopaminergic axons in brain tissue slices from rhesus macaques.^86^ Although PYR-C3-CIT already improves on PYR-C6-CIT, non-specific staining in tissue still remains. In the design of fluorescent conjugates, the linker itself could also be changed from an aliphatic chain to a more polar chain, *e.g.*, PEG or peptide-containing linker, to further improve labeling properties.^5,60,63,87,88^ Such strategies can be employed in designing the next generation of fluorescent drug conjugates. Taken together, our results present significant forward steps both in the use of fluorescent drug conjugates in imaging endogenously expressed SERT, and in the development of PyrAte fluorophores towards imaging specific protein targets in live samples.

## Conclusions

We report for the first time the use of a PyrAte-based fluorophore–drug conjugate in imaging endogenous SERT *ex vivo* in living mouse brain tissues using two-photon microscopy. These compounds were synthesized by modifying a SSRI, (*S*)-citalopram, with a PyrAte fluorophore. We show through computational and experimental methods that a change in linker length leads to improvements in affinity, selectivity, and imaging properties. The use of a PyrAte fluorophore also enables the simultaneous detection of both SERT-specific and YFP signals in a two-photon imaging system. Although both conjugates displayed weaker selectivity compared to their parent ligand, our results show unprecedented SERT-specific staining, which can be further improved by changing the physicochemical properties of either the fluorophore or the linker. Such approaches can potentially lead to enhanced SERT selectivity and more specific staining in the future.

## Data availability

The data supporting this article have been included as part of the ESI.†

## Author contributions

OJVB, IS, NKS designed and performed experiments, and co-wrote the manuscript. XW, SAM, ML, MR, SR, YX, NK, KJ performed experiments. XW, PASM and MN designed experiments, analyzed results, and contributed to the manuscript. KS and DS co-supervised experiments and contributed to the manuscript. LG, NM, and HHS conceived and supervised the work, and contributed to the writing of the manuscript. All authors have given approval to the final version of the manuscript.

## Conflicts of interest

There are no conflicts to declare.

## Supplementary Material

SC-OLF-D4SC06949H-s001
