# Supplementary material for: Fluorescent PyrAte-(S)-citalopram conjugates enable imaging of the serotonin transporter in living tissue
Source: Chem Sci. 2025 Mar 3;16(14):6003–13. doi: 10.1039/d4sc06949h (PMC11891579; doi:10.1039/d4sc06949h)
Supplement: SC-016-D4SC06949H-s001 [file SC-016-D4SC06949H-s001.pdf]

Supplementary Information (SI) for Chemical Science.
This journal is © The Royal Society of Chemistry 2025

SUPPORTING INFORMATION

Fluorescent PyrAte-(*S*)-citalopram conjugates enable imaging of the serotonin transporter in living tissue

Oliver J. V. Belleza,[†,‡] Iakovos Saridakis,[†,§,||] Nadja K. Singer,[†,||,#] Xavier Westergaard,[¶,††] Sergio Armentia Matheu,[§,||] Miran Lemmerer,[§,||] Margaux Riomet,[§] Pedro A. Sánchez-Murcia,[#] Nina Kastner,[‡] Stefanie Rukavina,[§] Yi Xiao,[§,||,‡‡] Kathrin Jäntsch,[‡] Marco Niello,[‡] Klaus Schicker,[‡,**] David Sulzer,[¶,§§,##] Leticia González,[*,#] Nuno Maulide,[*,§,‡‡,¶¶] Harald H. Sitte[*,‡,†††,‡‡‡]

[‡] Center of Physiology and Pharmacology, Institute of Pharmacology, Medical University of Vienna, 1090 Vienna, Austria. [§] Institute of Organic Chemistry, University of Vienna, 1090 Vienna, Austria. [||] Vienna Doctoral School in Chemistry (DoSChem), University of Vienna, 1090 Vienna, Austria. [#] Institute of Theoretical Chemistry, University of Vienna, 1090 Vienna, Austria. [¶] Department of Psychiatry, Columbia University Irving Medical Center, New York, New York 10032, United States. [††] Department of Biological Sciences, Columbia University, New York, New York 10027, United States. [‡‡] CeMM Research Center for Molecular Medicine of the Austrian Academy of Sciences, 1090 Vienna, Austria. ** Division of Neurophysiology and Neuropharmacology, Medical University of Vienna, 1090 Vienna, Austria. [§§] Departments of Psychiatry, Neurology, and Pharmacology, Columbia Irving Medical Center, New York, New York 10032, United States. [##] Division of Molecular Therapeutics, New York State Psychiatric Institute, New York, New York 10032, United States. [¶¶] Research Platform NeGeMac, 1090 Vienna, Austria. [†††] Hourani Center for Applied Research, Al-Ahliyya Amman University, 19328 Amman, Jordan. [‡‡‡] Center for Addiction Research and Science – AddRess, Medical University Vienna, 1090 Vienna, Austria.

† These authors contributed equally.

* Correspondence should be addressed to: Harald H. Sitte (orcid.org/0000-0002-1339-7444) E-mail: harald.sitte@meduniwien.ac.at; Nuno Maulide (orcid.org/0000-0003-3643-0718) E-mail: nuno.maulide@univie.ac.at; Leticia González (orcid.org/0000-0001-5112-794X) E-mail: leticia.gonzalez@univie.ac.at

Table of Contents

1. Biological characterization

1.1. Experimental methods S3

1.2. Tables and Figures S6

Table S1. *Activity of PyrAte-(S)-citalopram conjugates in radioligand uptake inhibition assays* S6

Figure S1. *PYR-C6-CIT and PYR-C6-ME in SERT-expressing HEK293 cells* S7

Figure S2. *Co-incubation of PYR-C6-CIT and TMRM in SERT-expressing HEK293 cells* S8

Figure S3. *Radioligand binding assays* S9

Figure S4. *Non-inverted images of PYR-C6-CIT in mouse brain slices* S10

Figure S5. *PYR-C3-CIT and PYR-C6-CIT signal-to-noise ratios* S11

Figure S6. *Representative two-photon eYFP/PyrAte control images* S12

2. Computational methods

2.1 Docking studies and molecular dynamics S13

Figure S7. *Snapshots of ligands PYR-C3-CIT and PYR-C6-CIT inside hSERT* S14

Table S2. *Hydrogen bond occupancies of PYR-C3-CIT or PYR-C6-CIT to the hSERT* S15

2.2 Binding free energy estimation S16

Table S3. *Free energy change between the unbound and bound ligand to the hSERT* S16

2.3 Quantum chemical lipophilicity study S17

Table S4. *Octanol/water Gibbs free energy difference and partition coefficients* S17

Appendix S18

3. Chemical synthesis and characterization

Chemical Synthesis, Photophysical properties S34

Table S5. *Photophysical properties of PyrAtes* S35

Experimental procedures S36

3.1. Reduction of citalopram S36

3.2. General Procedures S36

3.3. Characterization data S38

NMR spectra S44

References S54

1. Biological characterization

1.1. Experimental methods

Materials and Chemicals

Buffer and solution compositions (in mM) are as follows:

Krebs-HEPES buffer (KHB): 120 NaCl, 3 KCl, 2 $CaCl_2$, 2 $MgCl_2$, 20 glucose, 10 HEPES NaOH, pH = 7.3 – 7.4.

Hypotonic lysis buffer (HME): 2 $MgCl_2$, 1 EDTA, 20 HEPES NaOH, pH = 7.5.

Binding assay buffer (B2): 120 NaCl, 3 KCl, 2 $MgCl_2$, 1 EDTA, 20 Tris HCl, pH = 7.5.

SERT wash buffer: 120 NaCl, 1 $MgCl_2$, 10 Tris HCl, pH = 7.4.

External solution: 140 NaCl, 3 KCl, 2.5 $CaCl_2$, 2 $MgCl_2$, 20 glucose, 10 HEPES NaOH, pH = 7.4.

Internal solution: 5.9 NaCl, 133 K^+ $HOCH_2(CHOH)_4COO^-$, 1 $CaCl_2$, 0.7 $MgCl_2$, 10 EGTA, 10 HEPES KOH, pH = 7.2.

Artificial cerebrospinal fluid (ACSF): 125 mM NaCl, 2.5 mM KCl, 1.5 mM $CaCl_2$, 1 mM $MgCl_2$, 25 mM $NaHCO_3$, 10 mM glucose, 1.25 mM NaH_2PO_4, pH = 7.4 (oxygenated with 95% O_2, 5% CO_2).

High-sucrose cutting solution: 10 mM NaCl, 2.5 mM KCl, 0.5 mM $CaCl_2$, 7 mM $MgCl_2$, 180 mM sucrose, 10 mM glucose, 1.25 mM NaH_2PO4, pH = 7.4 (oxygenated with 95% O_2, 5% CO_2).

Cell culture

Human embryonic kidney 293 cells (HEK293) stably expressing the mCherry-tagged, YFP-tagged, and GFP-tagged human isoforms of SERT (mCherry-SERT, YhSERT, GhSERT), DAT (YhDAT), NET (hNET), and OCT3 (YhOCT3) were cultured in Dulbecco's modified Eagle's medium (DMEM) with high glucose (4.5 g/L) and l-glutamine (584 mg/L), supplemented with 10% fetal calf serum (FCS), 100 units/mL penicillin and 100 µg/mL streptomycin. The cells were cultured in a humidified atmosphere (37 °C, 5% CO_2). Geneticin (50 µg/mL), Blasticidin (6 µg/mL), and Zeocin® (150 µg/mL) were added to maintain the selection pressure for the stable cell lines. Cells were typically seeded 24 hours prior to the experiments. For uptake inhibition experiments, cells were seeded at 3.6 x 10^4 cells/0.2 mL into 96-well plates coated with poly-d-lysine (PDL). For microscopy experiments, cells were seeded at 2.5 x 10^4 cells/0.25 mL into PDL-coated 8-well polymer coverslip slides (ibidi GmbH, Gräfelfing, Germany). For electrophysiology, cells were seeded at a low density into PDL-coated 3-cm Petri dishes.

Radioligand-based assays

For uptake inhibition experiments, the cell medium was aspirated and the cells were washed once with 0.2 mL/well KHB at room temperature. The cells were pre-incubated with increasing concentrations of the substance of interest, diluted in KHB (0.05 mL/well). The pre-incubation solution was replaced with 0.05 mL/well KHB solution still containing the substance of interest and additionally the tritiated substrate (100 nM $[^3H]$DA for YhDAT; 100 nM $[^3H]$5HT for YhSERT; 20 nM and 50 nM $[^3H]MPP^+$ for hNET and YhOCT3), after 6 minutes (YhSERT), 5 minutes (YhDAT, hNET), or 10 minutes (YhOCT3). Substrate uptake was stopped after one minute (for $[^3H]$DA and $[^3H]$5HT), three minutes, or

ten minutes (for [^{3}H]MPP$^+$), by exchange of the substrate-containing buffer with 0.2 mL ice-cold KHB. The KHB was aspirated immediately. Cells were lysed and prepared for [^{3}H] content analysis by adding 0.2 mL Ultima Gold™ XR liquid scintillation cocktail. Measurements were performed in a 1450 MicroBeta microplate liquid scintillation counter. Non-specific uptake was determined in the presence of 30 µM paroxetine (for YhSERT), 50 µM GBR12909 (for YhDAT and hNET), or 100 µM decynium-22 (for YhOCT3).

For radioligand binding assays, membranes were first prepared from HEK293 cells stably expressing YhSERT. Cells were harvested from dishes using a plastic scraper and ice-cold PBS. Cells were recovered as a pellet after centrifugation (400× g) for 10 min at 4°C, and then re-suspended in HME buffer. The cell suspension was frozen rapidly by immersing in liquid N_2, and then immediately thawed and sonicated for three 12 pulses/min cycles. Membrane fractions were collected after centrifugation at 40000× g for 15 minutes, and then re-suspended in HME buffer. Protein concentration was determined using Coomassie Brilliant Blue kit (BioRad Laboratories, California, USA). The binding reactions were carried out in a final volume of 0.25 mL B2 buffer containing 15 µg membranes, 3 nM [^{3}H]-imipramine, and increasing concentrations of test compound, for 60 min at 20 – 22 °C. The reactions were terminated by filtration through polyethylenimine-coated glass fiber filters using a Skatron cell harvester and cold SERT wash buffer. The amount of radioactivity in the filters was measured using a Tri-Carb 2800TR liquid scintillation analyzer (PerkinElmer). The K_i was calculated from the measured IC_{50} using the Cheng-Prusoff equation.

Confocal microscopy

Prior to imaging, cell culture medium was replaced with pre-warmed KHB. HEK293 cells expressing mCherry-SERT were imaged using a Nikon A1R laser scanning confocal microscope equipped with a GaAsP detector, using a 60× oil-immersion objective. PyrAtes dissolved in DMSO were diluted in KHB (0.1% v/v) to make solutions with the desired final concentration (20 nM, 100 nM), which were added to each well containing the cells. To test specific binding, cells were pre-incubated with 10 – 30 µM paroxetine for 10 minutes. PyrAtes were excited using a 405 nm laser, and its fluorescent signals collected using 525/50 nm emission filters; mCherry was excited using a 561 nm laser, and fluorescence was collected using 595/50 nm emission filters. Images were captured prior to and at specific time points after addition of the PyrAte compounds using NIS-Elements software. Images were processed using ImageJ and further co-localization analysis was performed using the JACoP plug-in.[1]

Electrophysiology

5HT-mediated currents were measured at room temperature (20-25°C) using whole-cell patch clamp in HEK293 cells stably expressing SERT. Cells were voltage-clamped (−60 mV) and continuously superfused with a physiological external solution containing 140mM NaCl, 2.5 mM CaCl2,2mM MgCl2, 20mM glucose and 10mM HEPES, pH= 7.4. Micropipettes were made from borosilicate glass capillaries with a resistance ranging from 2.5 – 6 MΩ using a P-97 micropipette puller (Sutter Instrument), which were then filled with an internal solution containing 133mM K-gluconate, 6mM NaCl, 1mM CaCl2, 0.7 mM MgCl2, 10mM HEPES, 10mM EGTA, pH= 7.2. For the measurements we used an amplifier Axopatch 700B and pClamp 11.2 software (MDS Analytical Technologies, Sunnyvale, CA, USA). The compounds were diluted the day of the experiments in external solutions and applied using a DAD-12 superfusion system with a 8-tube perfusion manifold (ALA Scientific Instruments, Farmingdale, NY, USA), guaranteeing rapid solution exchange. Current traces were filtered at 1 kHz and digitized at 10 kHz using a Digidata 1550 (MDS Analytical Technologies). Current amplitudes in response to application of test compounds or 5-HT were quantified using Clampfit 10.2 software (Molecular Devices, San Jose, CA,

USA). The effect of the drug on SERT-mediated current was analysed by normalizing the current elicited by a saturating concentration of 5HT (10 µM) following a pre-incubation with the drug of interest over the current elicited by 5HT (10 µM) before drug application. The drug was applied around the IC_{50} concentration. Electrophysiology data was analyzed using Clampfit 10.2.

Animal Protocols

All animal protocols were approved by the Institutional Animal Care and Use Committee (IACUC) of Columbia University, following guidelines established in the NIH Guide for the Care and Use of Laboratory Animals. All animals were caged in groups of 5 or less in a 12-hr light/dark cycle with access to food and water *ad libitum*.

Acute mouse brain slices preparation

Wild-type C57/BL-6 (The Jackson Laboratory, Bar Harbor, ME) and Pet1-eYFP mice were sacrificed by cervical dislocation at ages > 12 weeks. Pet1-eYFP mice (Cg-Tg(Fev-cre)1Esd/J (ePet-cre; The Jackson Laboratory) crossed with Ai32(RCLChR2(H134R)/EYFP (Ai32; The Jackson Laboratory) maintained on a 129SvEv/Tac background) were kindly provided by Dr. Mark S. Ansorge (Columbia University Irving Medical Center; Department of Psychiatry, New York, NY 10032). Mice were decapitated, and the brain was removed and placed in an ice-cold sucrose cutting solution. Coronal brain slices with 300 µm thickness were prepared using a Leica VT1200 vibratome at 0 – 4 °C. Slices containing the striatum, substantia nigra, and the raphe were transferred to oxygenated ACSF solution and allowed to equilibrate at room temperature.

Two-photon microscopy

Slices were incubated with PyrAte-(*S*)-citalopram conjugates (500 nM in ACSF) for 30 minutes at room temperature. For inhibition experiments, escitalopram (2 µM) and paroxetine (30 µM) were incubated for 15 minutes prior to addition of the test compound. Each slice was then transferred to an imaging chamber and held in place by a stainless-steel slice anchor (Warner Instruments). ACSF solutions containing the test compounds were continuously perfused throughout the imaging experiments. Structures at depths between 20 to 30 µm from the top of the slice were visualized with fluorescent signals using a Prairie Ultima multi-photon microscopy sytem: PyrAte was imaged using 810 nm excitation wavelength and 525/50 nm emission filter, eYFP was imaged using 965 nm excitation wavelength and 525/50 nm emission filter. Images were captured using Prairie View software and processed with ImageJ. Further co-localization analysis was performed using the JACoP plug-in.[1]

Statistical and data analysis

Data treatment and statistical analysis were performed using Microsoft Excel 2016 and GraphPad Prism v10.0. Data and statistical tests as shown in Figure 2 include individual points, mean, SEM, and $P = 0.0023$ as determined using two-tailed, paired t-test ($t = 5.690$, $df = 5$). Data and statistical tests as shown in Figure 5 include individual values, mean, SEM, and P values as determined using two-tailed unpaired t-test with Welch's corrections (Fig. 5A: $P = 0.0166$, $t = 4.665$, $df = 3.157$; Fig. 5B: $P = 0.0147$, $t = 5.220$, $df = 2.913$; Fig. 5C: $P = 0.0064$, $t = 4.097$, $df = 5.987$).

1.2. Tables and Figures

Table S1. *Activity of PyrAte-(S)-citalopram conjugates was tested in radioligand uptake inhibition assays, along with unconjugated PyrAte and (S)-citalopram. Shown here are the mean IC_{50} values and the standard deviation in parentheses from N = 3 – 6 independent experiments, in µM.*

	YhSERT	YhDAT	hNET	YhOCT3
(*S*)-citalopram	0.04 (0.01)	> 100	11.9 (5.38)	n.d.
PyrAte-C6-(*S*)-citalopram (**PYR-C6-CIT**)	0.83 (0.11)	2.05 (0.66)	12.6 (3.49)	2.10 (0.27)
PyrAte-C3-(*S*)-citalopram (**PYR-C3-CIT**)	0.40 (0.05)	3.57 (0.91)	10.9 (2.36)	3.32 (0.25)
PyrAte-C6-methylester (**PYR-C6-ME**)	11.2 (0.42)	6.38 (0.46)	8.75 (2.14)	2.37 (0.46)

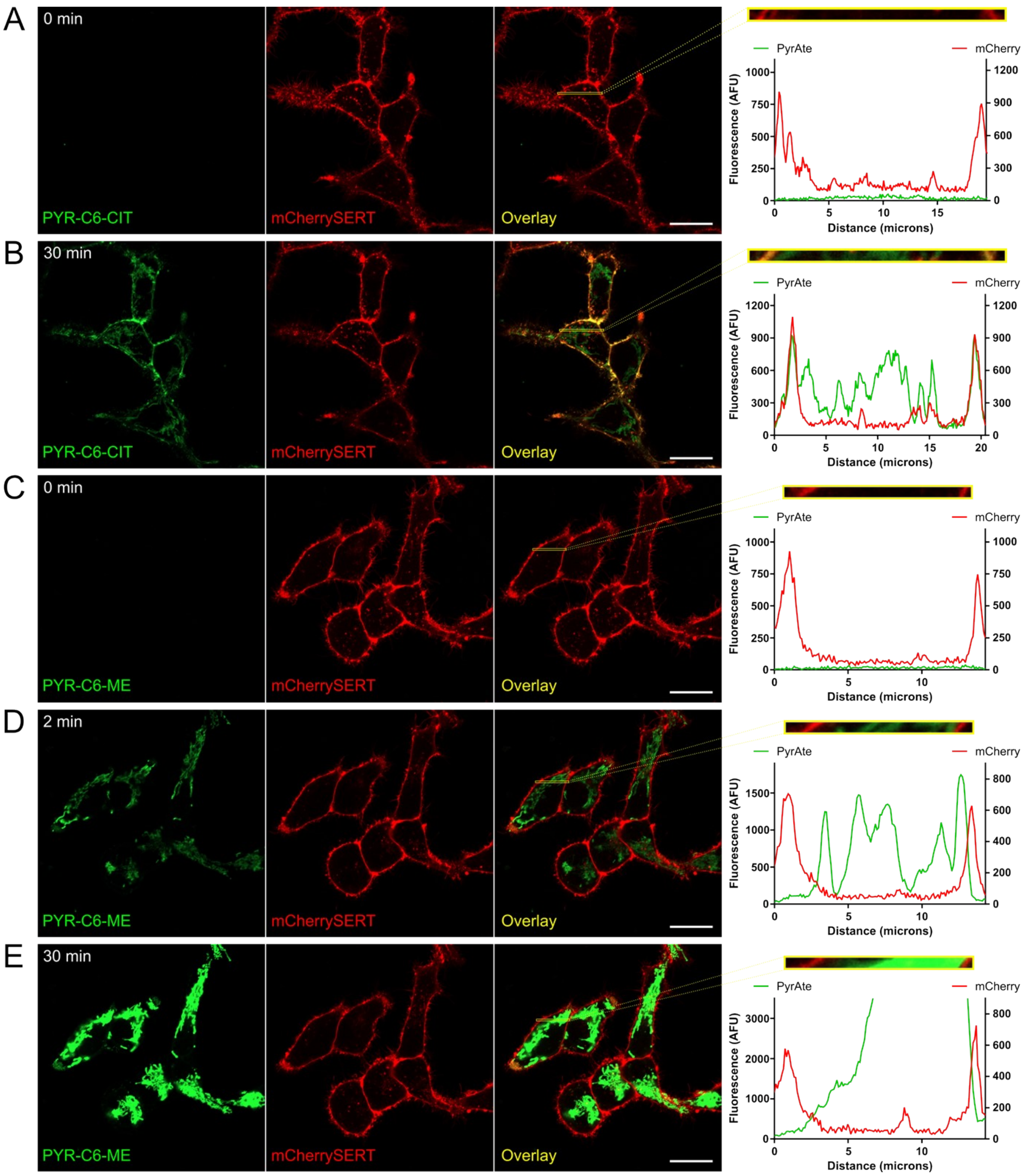

*<u>Figure S1.</u> (A-B) Addition of 100 nM PyrAte-C6-(S)-citalopram (**PYR-C6-CIT**) to mCherrySERT-expressing HEK293 cells results in membrane staining and intracellular accumulation. (C-E) In contrast, membrane staining is absent when 100 nM of the unconjugated PyrAte-C6-methylester (**PYR-C6-ME**) is added to the cells. Rapid internalization is instead observed. PyrAte is displayed in green (Ex. 405 nm, Em. 525/50 nm) and mCherry in red (Ex. 561 nm, Em. 595/50 nm). Overlap of signals is observed as yellow. Representative images of at least three different experiments are shown. Scale bar = 20 µm.*

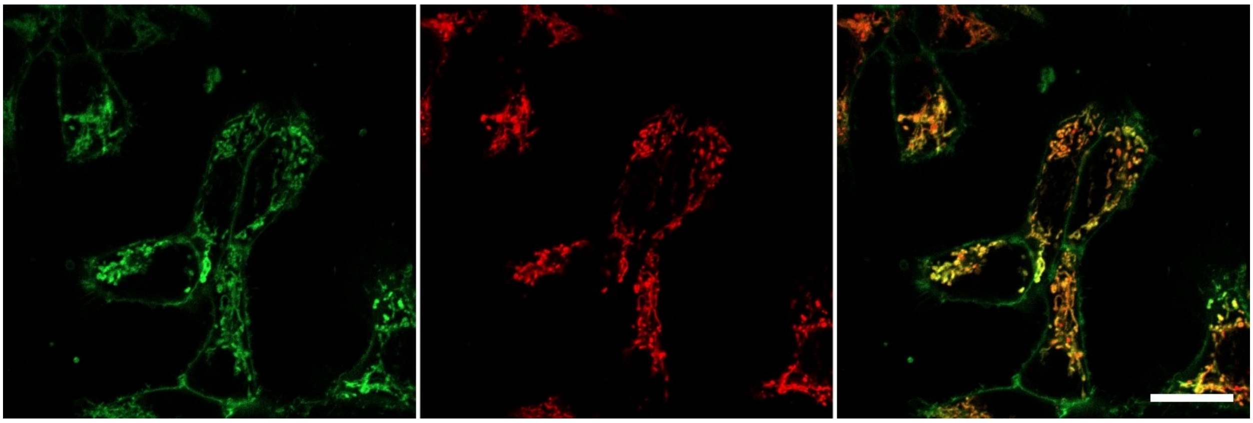

Figure S2. *Co-incubation of GhSERT HEK cells with 100 nM PyrAte-C6-(S)-citalopram (**PYR-C6-CIT**) and 20 nM of the mitochondrial marker TMRM for 30 mins reveals co-localization. Both compounds therefore accumulate in the mitochondria. PyrAte is displayed in green (Ex. 405 nm, Em. 525/50 nm) and TMRM in red (Ex. 561 nm, Em. 595/50 nm). Representative images of at least three different experiments are shown. Scale bar = 20 µm.*

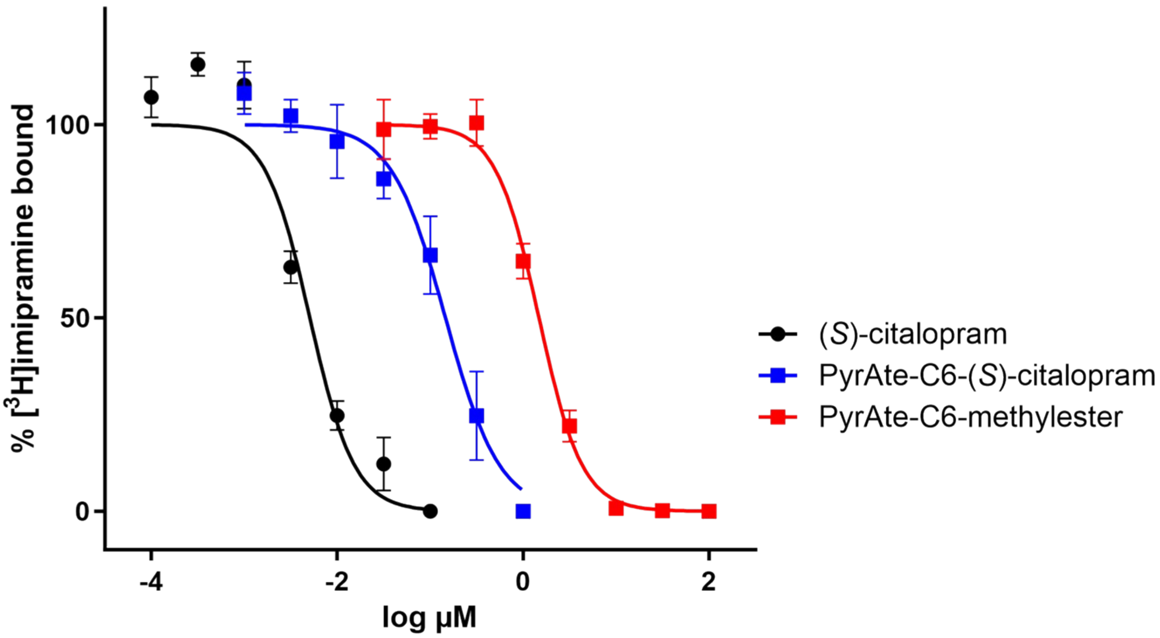

Figure S3. *Radioligand binding assays show PyrAte-C6-(S)-citalopram (**PYR-C6-CIT**), as well as naked PyrAte-C6-methylester (**PYR-C6-ME**) have a binding affinity to hSERT, with K_i of 0.11 ± 0.07 and 0.73 ± 0.25 µM, respectively. This is compared to (S)-citalopram with K_i of 0.0034 ± 0.0017 µM. The K_i values represent mean and standard deviation obtained from three independent experiments performed in duplicates. Data points in the graph show mean and SEM.*

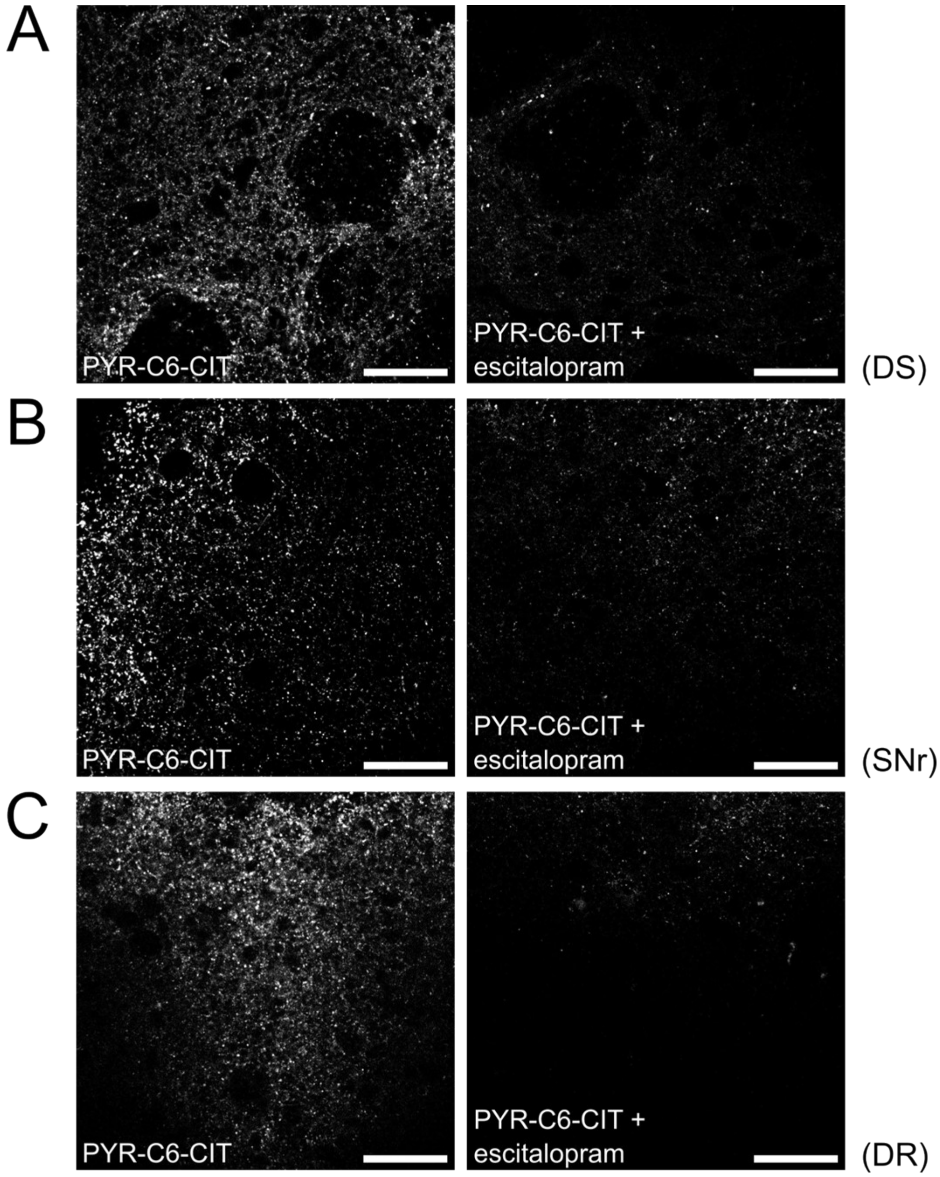

*Figure S4. Non-inverted images showing SERT-specific staining of **PYR-C6-CIT** in the (A) dorsal striatum (DS), (B) substantia nigra reticulata (SNr), and (C) dorsal raphe (DR) nucleus in acute coronal mouse brain slices at 500 nM concentration. Signals are reduced in the presence of the SERT inhibitor, escitalopram. PyrAte was imaged at 810 nm (Ex.) and 525/50 nm (Em.) using a two-photon microscope. Scale bar = 50 µm.*

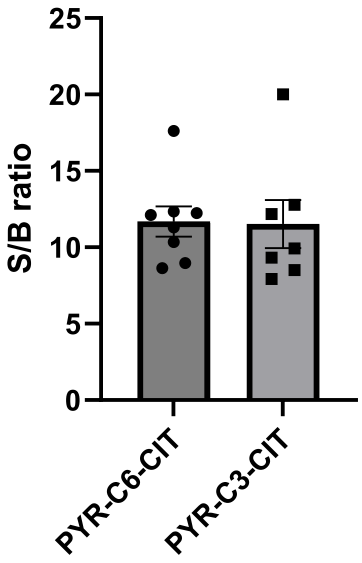

S/B measured from DS images
using 500 nM concentration

S/B ratio similar to PYR-C6-CIT
was achieved with PYR-C3-CIT
at a lower laser power
~ 2.8 mW for PYR-C6-CIT
~ 1.1 mW for PYR-C3-CIT

*<u>Figure S5.</u> Signal-to-background ratios were evaluated in the images of dorsal striatum (DS) obtained from WT mice using **PYR-C6-CIT** and **PYR-C3-CIT**. Similar ratios were observed in the images obtained using optimized imaging parameters.*

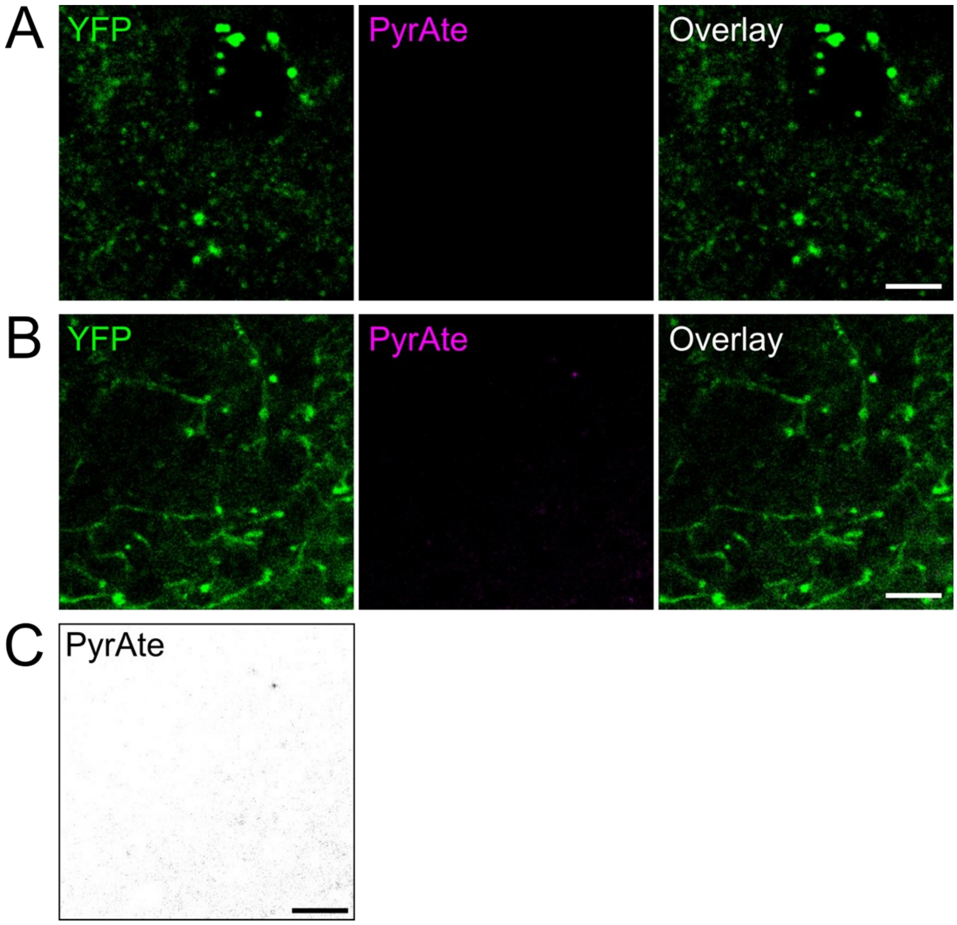

Figure S6. *Representative two-photon microscopy images showing the lack of signal cross-over of the eYFP (Ex. 965 nm) and PyrAte (Ex. 810 nm) channels both detected at 525/50 nm (Em.). Images were obtained from coronal brain slices of Pet1-Cre/Chr2-eYFP mice containing the (A) dorsal striatum and (B) substantia nigra reticulata, prior to addition of compounds. (C) Some weak signals arising from autofluorescence can also be detected from the PyrAte channel in Panel B, as seen in the corresponding inverted image in Panel C. Scale bar = 10 µm.*

2. Computational methods

2.1 Docking Studies

The docking studies were performed with AutoDock 4.2.6[2] using 50 Lamarckian genetic algorithm (LGA) runs for each ligand inside the combined orthosteric and allosteric serotonin binding site of hSERT (PDB entry 5I73 chain A, referred to hereafter as 5I73).[3] Subsequently, the best docking poses were used for a subsequent molecular dynamics (MD) minimization of the ligands inside 5I73 using AMBER17[4] and restraining the Cα-atoms. The ligand force field has been obtained using AMBER atom types with AM1-BCC[5] charges as implemented in AmberTools,[4] while for the protein the ff14SB force field[6] was used.

All-Atom Molecular Dynamics

To build an all-atom MD setup of the hSERT including the membrane, water, and ions, we have built an initial model of hSERT from the outward-open X-ray structure (PDB entry 5I71 chain A)[3] according to Singer et al.[7] The ligands (**PYR-C3-CIT** and **PYR-C6-CIT**) were placed in the newly built hSERT system by aligning it with the previously obtained minimized system. The final system contained ca. 150,000 atoms including 348 lipids, 91 sodium, 89 potassium and 184 chloride ions as well as 33,506 water molecules with box dimensions of ca. 12 nm^3. The two separate equilibrium MD simulations have been run using the NAMD code,[8,9] the lipid and protein being represented by the Amber Lipid14[10] and the ff14SB force field,[6] respectively. Water was modeled with TIP3P[11] and the ion parameters of Li/Merz were used.[12] Each system was minimized and equilibrated for 18 ns with a time step of 2 fs using a position restraint energy function on the protein backbone, lipids, and ligand (progressively relaxing the harmonic constrains by constraint scaling: 1, 0.5, and 0.1; 6 ns each). The final production of 100 ns was carried out under isobaric and isothermal conditions (NPT) and integrating Newton's equations of motion with a time step of 4 fs using Hydrogen Mass Repartitioning (HMR)[13] in combination with Rattle[14] and Shake[15] at 300 K and 1 atm.

Additionally, we have analyzed the hydrogen bonds that are involved in the binding of the ligands with the HBonds Plugin Version 1.1 in VMD1.9.4.[16] The donor-acceptor distance was set to 3.5 Å and the angle cutoff was set to 30°. The hydrogen bonds are shown in Figures S4C and D and the analysis is summarized in Table S2.

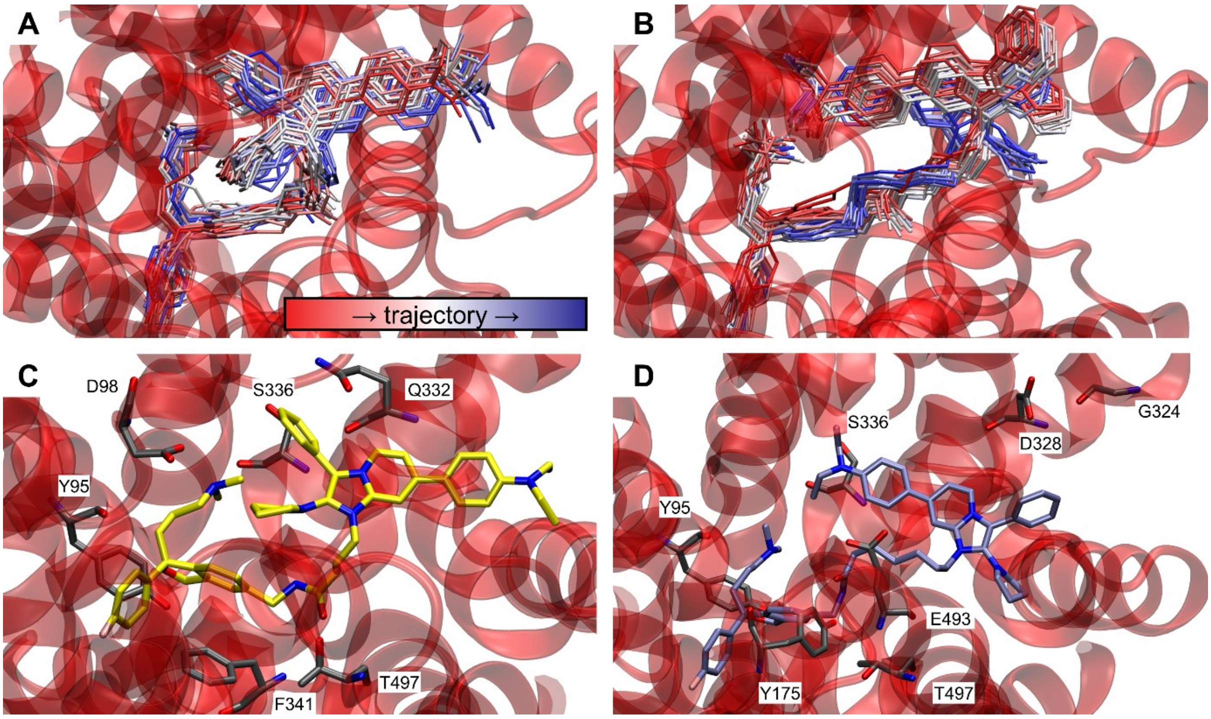

Figure S7. *25 equidistant snapshots of ligands* ***PYR-C3-CIT*** *(A) and* ***PYR-C6-CIT*** *(B) inside the hSERT obtained along the 100 ns MD simulation. The ligands are colored according to the occurrence in the trajectory (see panel in A, start is red, end is blue). The residues involved in hydrogen bonding with ligands* ***PYR-C3-CIT*** *and* ***PYR-C6-CIT*** *are shown in panels (C) and (D), respectively.*

*Table S2. Hydrogen bond occupancies of the ligands **PYR-C3-CIT** or **PYR-C6-CIT** to the hSERT given as a percentile of 2500 snapshots taken from the 100 ns MD simulation. Only values above 5% are shown.*

PYR-C3-CIT			PYR-C6-CIT		
donor	acceptor	occupancy	donor	acceptor	occupancy
T497	**PYR-C3-CIT**	57.84%	T497	**PYR-C6-CIT**	52.84%
Q332	**PYR-C3-CIT**	23.52%	Y175	**PYR-C6-CIT**	37.84%
**PYR-C3-CIT**	D98	12.00%	**PYR-C6-CIT**	D328	24.24%
**PYR-C3-CIT**	Y95-Main	11.48%	Y95	**PYR-C6-CIT**	17.40%
S336-Main	**PYR-C3-CIT**	9.60%	**PYR-C6-CIT**	Y175	9.24%
**PYR-C3-CIT**	S336-Main	8.32%	**PYR-C6-CIT**	Y95-Main	7.48%
Y95	**PYR-C3-CIT**	6.32%	**PYR-C6-CIT**	E493-Main	6.88%
**PYR-C3-CIT**	F341	5.88%	**PYR-C6-CIT**	S336-Main	6.88%
			**PYR-C6-CIT**	G324-Main	5.92%

2.2 Binding Free Energy Estimation

The free energy change between the unbound and bound ligand to the hSERT is calculated using the Molecular Mechanics / Poisson Boltzmann Surface Area (MM/PBSA) method,[17,18] a post-processing trajectory analysis technique. The method is computationally inexpensive but assumes that the configurational space explored by the receptor and ligand is unchanged between the bound and unbound states. The MM/PBSA analysis was performed using the MMPBSA.py script[19] implemented in AMBER20[20] for 500 snapshots taken from the last 20 ns of the MD simulations. The MMPBSA.py script was run with input values inspired by the "sample input for MMPBSA with membrane proteins" given in the AMBER20 manual.[20] The membrane thickness was set to 36 Å, it was centered to the center of the protein, and a dielectric constant of 7 was assigned to the membrane. The Periodic Incomplete Cholesky Conjugate Gradient (PICCG) iterative solver was chosen and the total electrostatic energy and forces were computed with the particle-particle particle-mesh (P3M) procedure.[21] The atom-based cutoff distance for van der Waals interactions was set to 99 Å and the atom-based cutoff distance to remove short-range finite-difference interactions as well as to add pairwise charge-based interactions was set to 7 Å. The MM/PBSA results are given in Table S3 broken up into components of the energy and entropy contributions.

Table S3. Free energy change between the unbound and bound ligand to the hSERT in kcal mol^{-1} given as the average of 500 snapshots taken from the last 20 ns of the MD simulation. The energy and entropy contributions are broken up into their components. The standard deviation (std. dev.) and standard error of mean (error) are given.

ligand	PYR-C3-CIT			PYR-C6-CIT		
energy Component	average	std. dev.	error	average	std. dev.	error
van der Waals	-99.0	4.8	0.2	-111.6	3.3	0.1
electrostatic	51.4	13.6	0.6	65.4	8.6	0.4
polar solvation	0.0	0.0	0.0	0.0	0.0	0.0
non-polar solvation	-9.1	0.3	0.0	-9.4	0.1	0.0
dispersion solvation free	0.0	0.0	0.0	0.0	0.0	0.0
total gas phase free	-47.6	13.2	0.6	-46.2	9.1	0.4
total solvation free	-9.1	0.3	0.0	-9.4	0.1	0.0
total	-56.6	13.3	0.6	-55.7	9.1	0.4

2.3 Quantum Chemical Lipophilicity Study

In order to measure lipophilicity, we have calculated the octanol/water partition coefficient $\log P_{o/w}$ quantum mechanically with density functional theory for **PYR-C3-CIT**, **PYR-C6-CIT**, and the unconjugated PyrAte-C6-methylester **PYR-C6-ME**. We used the following formula,[22] which includes the ground state Gibbs free energies ΔG^0 of the ligand in water (ΔG^0_w) and n-octanol solvation (ΔG^0_o).

$$\log P_{o/w} = \frac{-\Delta G^{0}_{o/w}}{2.303RT} = \frac{-(\Delta G^0_o - \Delta G^0_w)}{2.303RT},$$

To calculate the Gibbs free energies, a geometry optimization had to be done for each of the ligands (**PYR-C3-CIT**, **PYR-C6-CIT**, and **PYR-C6-ME**). The initial geometries for **PYR-C3-CIT** and **PYR-C6-CIT** were obtained from the final snapshot of the MD simulations. The initial geometry for **PYR-C6-ME** was obtained by using the **PYR-C6-CIT** geometry and changing the respective atoms with GaussView6.[23] The initial geometries were pre-optimized using the RI-BP86-D3BJ/def2-SVP method[24–28] and further optimized with the B3LYP-D3BJ/def2-SVP@SMD(water/n-octanol) approach[22,26–29] using the ORCA 5.0 software[30,31] (XYZ geometries are given in the Appendix, Supporting Information). The Gibbs free energies were calculated by performing the final energy and frequency calculations at the B3LYP-D3BJ/def2-TZVP@SMD(water/n-octanol)[22,26–29] level of theory with the Gaussian 16 suite at 296.15 K. We used the solvation model based on density (SMD) as it uses the full solute electron density to compute the cavity-dispersion contribution instead of the area only.[22] The resulting calculated octanol/water Gibbs free energy differences $\Delta G^{0}_{o/w}$ and partition coefficients $\log P_{o/w}$ for ligands **PYR-C6-ME**, **PYR-C3-CIT**, and **PYR-C6-CIT** are summarized in Table S4.

Table S4. Octanol/water Gibbs free energy difference $\Delta G^{0}_{o/w}$ and partition coefficient $\log P_{o/w}$ for ligands **PYR-C6-ME***, **PYR-C3-CIT**, and **PYR-C6-CIT** calculated at the B3LYP-D3BJ/def2-TZVP@SMD(water/n-octanol) level of theory.*

	$\Delta G^{0}_{o/w}$ / kcal mol^{-1}	$\log P_{o/w}$
**PYR-C6-ME**	-10.6	7.8
**PYR-C3-CIT**	-11.5	8.4
**PYR-C6-CIT**	-14.4	10.6

Appendix

Table A-1. XYZ coordinates of **PYR-C6-ME**@SMD(water)

Element	X	Y	Z
C	7.15554740406763	0.60710743958357	4.87129390599069
O	7.53962215905694	1.71394267319641	5.21272539835458
C	7.71548611405611	-0.66767322145328	5.45071836904537
H	8.75427152127580	-0.44756283842684	5.73300499443039
H	7.73262874557724	-1.46911732969428	4.69987224239013
C	6.91569087078864	-1.13506538094281	6.68935669693251
H	7.36442010168837	-2.08220915389240	7.03273746208003
H	5.88474771481646	-1.37274281214374	6.38200701121759
C	6.87713734628097	-0.12817648963063	7.84115896223374
H	6.11950754230832	-0.45726456134086	8.57237731727444
H	6.51641236649191	0.84280755667943	7.46423520099542
C	8.20869771281316	0.06127044454683	8.57947251634591
H	9.04626301716599	0.11557768789095	7.86209740388567
H	8.40297695330552	-0.82709605472162	9.20259070072287
C	8.21766736503825	1.32442858172509	9.45066842287158
H	8.93480671745013	1.22354968986711	10.28005909175992
H	7.22344694560081	1.46405261086566	9.90775611522189
C	8.56620963093468	2.56805106708266	8.62307375510934
H	8.18902074565062	2.46759719216167	7.59925437462141
H	9.64905611007459	2.69214463984735	8.52470256151178
N	7.98541434057250	3.80435399782981	9.15790975923214
C	8.32855871936358	4.48230363744944	10.33744543159505
N	9.44772778615946	4.21162314655872	11.05320941662302
C	9.57494587698443	4.58231928478568	12.47290966087627
C	10.89409996228154	3.93154481609113	12.88029014316370
C	11.73628680305285	4.08551164589475	11.61037799341570
C	10.73823978716889	3.75813803604287	10.49704211861904
H	10.96305565840197	4.27476639012418	9.55204782981373
H	10.72851275251848	2.67265955731942	10.30692606463438
H	12.61481835105481	3.42542203198891	11.57955121098499
H	12.08351000301028	5.12698260415775	11.51386166043806
H	10.74048321307063	2.86434877741342	13.11050364142602
H	11.33763175898367	4.41579789889382	13.76194186963100
H	8.70222778803388	4.22499228053909	13.04003063392112
H	9.63568989333904	5.67501656433639	12.60026262199989
C	7.29321588764119	5.36822389886853	10.63654985736285
C	7.15373636597200	6.41403568760365	11.66214123663998
C	6.07903158148060	6.39298477070674	12.57133412489267
C	5.94883163512111	7.39165422432677	13.53835849296070
C	6.89313947022965	8.42144928038278	13.61736199894290
C	7.96273062502340	8.45350579818918	12.71722472180953
C	8.08828312946143	7.46254986767771	11.74061849798679
H	8.91420330876065	7.49737705810771	11.02691966850247
H	8.70018322701220	9.25816785526547	12.76966229723665
H	6.79414278539011	9.19869762191962	14.37911105009413
H	5.11247939964800	7.35989283061621	14.24091530860602
H	5.35095977437031	5.57958062130017	12.52542189035071
N	6.31142337057801	5.16537871470974	9.65138959447086
C	5.10416468146105	5.78279292405554	9.46562386099573
C	4.30141210088728	5.41298471517661	8.41815906063260
H	3.35295424819942	5.93622727375381	8.30685331019622

H	4.83769487667496	6.55875835241130	10.18096839007534
C	6.74364062715127	4.21700304959438	8.75793285584217
C	5.95436371455191	3.81684060482768	7.68504989063104
H	6.33032787305886	3.04295496934447	7.02257035344464
C	4.69223748090076	4.39553745688962	7.49491998608155
C	3.81343251409543	3.95603277225886	6.40169084275491
C	4.29005151641778	3.12874209161482	5.36054098054548
C	3.46439982770577	2.65513024605533	4.35450474495223
H	3.90264869740184	2.01332528213890	3.59329557568838
H	5.34180349012146	2.84468090479486	5.31806820540376
C	2.44609844774328	4.30144562676996	6.34792999378290
H	2.00964150085241	4.93616591768599	7.12124869885574
C	1.60456605425825	3.84228172356745	5.34368458474436
H	0.56080074915770	4.15169924982687	5.36313930414152
C	2.08057663564023	2.98417322780755	4.31344631796902
N	1.25601686394526	2.49815729140281	3.33311444607618
C	1.75713088824841	1.61454370920299	2.28325518670562
C	1.83958209779753	0.14846197133316	2.70308233250312
H	0.84712363053474	-0.23112628203989	2.99572208678372
H	2.20907493710478	-0.46837188940249	1.86804135642395
H	2.52221444721019	0.01291883596081	3.55637198581753
H	2.74024541498141	1.96976690450276	1.93852546196966
H	1.08353305731053	1.71762608720166	1.41961572991378
C	-0.17432562734090	2.79311049673251	3.31776290751148
H	-0.56318836105141	2.79046087135435	4.34765109559234
H	-0.67650128268376	1.95815536409193	2.80654091211278
C	-0.52071844728491	4.10703096296540	2.62222916909028
H	-0.04504384687901	4.96373453283573	3.12538332412754
H	-0.18159565840446	4.09628638522536	1.57367414832486
H	-1.61089515024065	4.26851362783112	2.62824767343634
O	6.15767327905209	0.55412666109253	3.97536571696762
C	5.63553891744383	-0.68114963264425	3.46495441422481
H	5.19196846891737	-1.28723103240807	4.26697380966935
H	4.85429300987859	-0.40117085249471	2.74818638930959
H	6.41787798605335	-1.25252104161799	2.94519359747541

Table A-2. XYZ coordinates of **PYR-C6-ME**@SMD(n-octanol)

Element	X	Y	Z
C	7.04530498565339	0.47076053700655	5.09998935744227
O	7.34774048280159	1.60245564497840	5.43632282907810
C	7.77339159476794	-0.75027868723983	5.60771254335265
H	8.81001605848615	-0.43523097350364	5.79269573932729
H	7.79614844251162	-1.54524320996104	4.84970827996104
C	7.14109439583113	-1.29371642917108	6.91062330522566
H	7.71293574817945	-2.18995121670847	7.20584387111127
H	6.11640495818113	-1.63870124794468	6.69644118770499
C	7.10229994056459	-0.29336949003535	8.06910874438671
H	6.44984106752829	-0.69678050974694	8.86228543030381
H	6.61093166675169	0.63457281151672	7.73118386061228
C	8.46814892703706	0.03816784387356	8.68741183046164
H	9.23951262504193	0.13118405272524	7.90250364208669
H	8.79001660674699	-0.80193716457842	9.32498907119916
C	8.43572627318817	1.33417435967308	9.50809362522406
H	9.24463718065764	1.34838990262116	10.25495382197013
H	7.48836154781602	1.38984158133306	10.07058799266291
C	8.55321371153343	2.56428710378569	8.59676501015007
H	8.07960601282734	2.36882027327064	7.62846817932761
H	9.60249389883294	2.79289385178538	8.37392137160145
N	7.90787031701962	3.75910599954334	9.14395659538777
C	8.26697831659438	4.45489769865981	10.30570973815902
N	9.41366477595190	4.18743554650329	10.98598103682013
C	9.53156457094440	4.38816141053459	12.43937050642421
C	10.94909215974415	3.90130143105368	12.73588857483185
C	11.71421740788934	4.34050856231113	11.48298880451464
C	10.73569841263470	4.00922972298885	10.35224077485881
H	10.84482774328797	4.66969793238864	9.47816740600681
H	10.88411064847767	2.96852271809820	10.01785643631869
H	12.68023676360482	3.83075408636558	11.35438955391100
H	11.90111439079317	5.42654165787029	11.51762149649472
H	10.96651585804145	2.80253433227525	12.82956508792608
H	11.34815252740620	4.33176425067071	13.66590827931610
H	8.74636976160466	3.82564815636952	12.96829976009476
H	9.43070930543034	5.45021746087175	12.71795230636867
C	7.22946352478325	5.32788139813132	10.61770098109610
C	7.10687959222233	6.36286524852260	11.65482378252652
C	6.01660222853012	6.37277750776120	12.54557411961538
C	5.91163465725642	7.36166542210615	13.52573596388213
C	6.89668513333468	8.34879062244410	13.63769631973685
C	7.98511868847426	8.34626963628988	12.75973422319893
C	8.08724513681437	7.36557711103340	11.77069292619209
H	8.92971878104155	7.37448066247289	11.07534581825922
H	8.75612731949114	9.11694934913657	12.83941000785132
H	6.81590049046654	9.11922144669614	14.40890491399237
H	5.06170081852651	7.35491482957381	14.21302300138436
H	5.25643394254252	5.59050251036300	12.47892239435110
N	6.23884881621583	5.11687001007297	9.64212984859928
C	5.03314974386870	5.73850911276094	9.45722059839695
C	4.23331401724618	5.37872419238012	8.40579457840813
H	3.29018359082786	5.91119713717767	8.29271872666609
H	4.76945760950174	6.51502391893378	10.17324178142165
C	6.66878261591066	4.16888979607694	8.74371376997869

C	5.87759920998714	3.77392912522588	7.66705160486810
H	6.25415183109173	3.00230944081307	7.00008024629646
C	4.62113096795975	4.36397753513532	7.47541162956142
C	3.74503054099693	3.95294109216257	6.36963909414048
C	4.21696973502332	3.13238704977263	5.32090620276849
C	3.39619575452527	2.69923168440217	4.29311152987504
H	3.83174402350284	2.05786598543031	3.52917816201517
H	5.26059229522573	2.81770215111838	5.29080655863679
C	2.38603525450312	4.32885380863058	6.30357384993631
H	1.94985387485164	4.95648164182364	7.08291180372031
C	1.55026164661839	3.91107315762494	5.27750788946217
H	0.51376282018442	4.24459934323687	5.28962738833045
C	2.02250719442810	3.06768611333326	4.23299928676699
N	1.20362764863209	2.63170516939594	3.22551883083018
C	1.70440483900410	1.78295226524122	2.14806013463840
C	1.72771531339122	0.29783286018377	2.50537918632734
H	0.71443374870752	-0.06544735985753	2.74330587203909
H	2.11191877683510	-0.29620845424429	1.65979861924017
H	2.36881396022439	0.10593912863823	3.38008745884320
H	2.70731639702562	2.12107136229889	1.84521563330829
H	1.05812101973417	1.94523681008571	1.27221748565458
C	-0.21485423238495	2.97501105256353	3.18594007231918
H	-0.62840781069892	2.95892554144739	4.20618172441876
H	-0.73356944172889	2.17182318837262	2.64061939043961
C	-0.49697783559907	4.31846987688020	2.51689176948851
H	-0.00389817290700	5.14551856968094	3.05234253122724
H	-0.13350999724558	4.32337144509363	1.47616851987443
H	-1.58073330703940	4.52050940356200	2.50040864778728
O	5.98626431666877	0.33087521463129	4.29043167898694
C	5.56256867915062	-0.93808061885379	3.77654270143477
H	5.30178768120896	-1.63469373426527	4.58603031610419
H	4.66537294087097	-0.73213021719098	3.17891759009945
H	6.33557453583339	-1.37848954449157	3.12924678437922

Table A-3. XYZ coordinates of **PYR-C3-CIT**@SMD(water)

Element	X	Y	Z
C	-7.8944786	-5.5715398	-2.2732345
H	-7.5483727	-5.3082524	-3.2724725
C	-8.5932931	-6.7666595	-2.0774547
H	-8.8012717	-7.447076	-2.905865
C	-9.0317213	-7.0833093	-0.7966301
F	-9.7140048	-8.2292843	-0.5978084
C	-8.7881421	-6.2466707	0.28865967
H	-9.1448504	-6.5278625	1.28154592
C	-8.0933917	-5.0547371	0.07533292
H	-7.9107004	-4.3988484	0.92779377
C	-7.6383243	-4.7000631	-1.2064188
C	-6.8705014	-3.3967279	-1.422701
C	-7.6527779	-2.2025759	-0.8186348
C	-7.0495558	-0.8185993	-1.0910084
C	-6.0361992	-0.3424328	-0.0496956
N	-5.3540989	0.89852127	-0.4299336
C	-4.7572923	1.53041232	0.73718948
H	-4.2954729	2.49050064	0.45804071
H	-3.965893	0.90841833	1.21054147
H	-5.5263929	1.73257397	1.49495023
C	-4.3249906	0.64415441	-1.4288365
H	-3.8726175	1.59215647	-1.7601625
H	-4.7470975	0.14779603	-2.3141601
H	-3.5064161	-0.0024543	-1.0403219
H	-5.2982325	-1.137665	0.1733355
H	-6.5737992	-0.1559116	0.89251111
H	-7.8660404	-0.0792608	-1.128011
H	-6.6029757	-0.8133401	-2.0959382
H	-7.7726265	-2.3600029	0.2645068
H	-8.662783	-2.2520088	-1.2552362
O	-6.7158759	-3.1564718	-2.8390137
C	-5.3533027	-2.9253211	-3.210381
H	-5.2369265	-1.9011148	-3.6090026
H	-5.064817	-3.631318	-4.0098949
C	-5.4382298	-3.4916921	-0.9254739
C	-4.946138	-3.8015537	0.34226891
C	-3.5727849	-3.6961438	0.5752094
H	-3.1826191	-3.9242125	1.56942291
H	-5.6138868	-4.1027125	1.15015439
C	-4.5652801	-3.1417322	-1.9531957
C	-3.1969299	-3.0091986	-1.7196026
H	-2.5224538	-2.6883623	-2.518151
C	-2.6925923	-3.2732332	-0.4379471
C	-1.2153756	-3.0975488	-0.1511632
H	-0.7549993	-2.4916033	-0.9460067
H	-0.7012469	-4.0697397	-0.1436203
N	-0.975242	-2.4887393	1.14574352
H	-1.437294	-1.6026471	1.33160475
C	-0.5133746	-3.1869826	2.20887498
O	-0.0550316	-4.3319771	2.11453494
C	-0.63057	-2.4983365	3.55213279
H	-0.9767891	-1.4610583	3.42826904
H	0.3688132	-2.4702573	4.01602833

C	-1.5975057	-3.2941517	4.43635814
H	-1.2049927	-4.3121713	4.58192496
H	-2.5636939	-3.3933519	3.91971981
C	-1.801072	-2.6863417	5.81986735
H	-0.8577082	-2.6988487	6.37780757
H	-2.5193642	-3.2798926	6.40103372
N	-2.2548808	-1.2922572	5.78007246
C	-3.3769689	-0.8077681	5.08962046
N	-4.275729	-1.6345969	4.48713649
C	-4.8886615	-1.3160065	3.183237
C	-6.2425946	-2.0183114	3.24263911
C	-5.9573673	-3.240382	4.12018364
C	-5.0173032	-2.6922074	5.19621628
H	-4.3567976	-3.474542	5.59776414
H	-5.5779073	-2.2589478	6.04478564
H	-5.4368966	-4.0119016	3.52894624
H	-6.8601871	-3.6938357	4.55359246
H	-6.6238822	-2.2765308	2.24590469
H	-6.9793874	-1.360893	3.73304921
H	-4.2573245	-1.7203221	2.37322625
H	-4.9817542	-0.2368073	3.02463536
C	-3.3176689	0.57991055	5.10097453
C	-4.2987351	1.56758338	4.63975397
C	-5.6636345	1.37117547	4.92773497
C	-6.6198503	2.28700281	4.48805917
C	-6.2295015	3.42322325	3.77032878
C	-4.8774506	3.62734292	3.479031
C	-3.9190812	2.7018917	3.89708029
H	-2.8728775	2.85035419	3.62300432
H	-4.5667723	4.50138396	2.90201462
H	-6.9785428	4.14344293	3.43280208
H	-7.6748348	2.11801352	4.7170059
H	-5.9687995	0.49414275	5.50213615
N	-2.1279653	0.91422341	5.76827481
C	-1.613811	2.14125334	6.09817553
C	-0.4417411	2.22429296	6.79691566
H	-0.0573917	3.21910137	7.0164281
H	-2.1802598	3.01293703	5.77862642
C	-1.4905353	-0.2361037	6.18140789
C	-0.2922789	-0.1837925	6.89369592
H	0.1680595	-1.1155264	7.21486989
C	0.27382072	1.05678382	7.20710117
C	1.54588079	1.16545911	7.93486803
C	2.47097681	0.10094349	7.98274561
C	3.67611692	0.1986959	8.66304593
H	4.35081606	-0.654856	8.63509725
H	2.26228018	-0.8271231	7.44628728
C	1.9102258	2.34372989	8.62049759
H	1.23236198	3.19902871	8.63623128
C	3.10647262	2.45596599	9.31378712
H	3.31622841	3.3926369	9.8268701
C	4.03674496	1.38111682	9.36859401
N	5.20532083	1.47244823	10.0868356
C	6.19492411	0.39712371	10.0420525
C	7.05776011	0.36034909	8.78067562
H	7.68054267	1.26341375	8.69433265

H	6.44174743	0.28205935	7.87116335
H	7.72896826	-0.513345	8.81109996
H	5.67936683	-0.5667724	10.1732315
H	6.83913703	0.51571173	10.9256092
C	5.63524604	2.75389893	10.646405
H	6.40752015	2.53239855	11.3969506
H	4.79668985	3.20337752	11.2013161
C	6.18072352	3.75528117	9.62802311
H	5.44155487	3.97609526	8.84230751
H	7.09219386	3.37674333	9.14169677
H	6.43126348	4.70279002	10.1321661

Table A-4. XYZ coordinates of **PYR-C3-CIT**@SMD(n-octanol)

Element	X	Y	Z
C	-7.563600298	-5.639327582	-2.526419711
H	-7.112213596	-5.312877459	-3.463596929
C	-8.143915455	-6.907757286	-2.443726603
H	-8.155773755	-7.586047187	-3.299898335
C	-8.715707531	-7.306322212	-1.239801241
F	-9.274424294	-8.529718956	-1.149710394
C	-8.726606606	-6.473468112	-0.12504983
H	-9.186575045	-6.816770949	0.804176305
C	-8.145673694	-5.207032295	-0.225649112
H	-8.157060088	-4.559287394	0.652107842
C	-7.554930436	-4.773252121	-1.423976101
C	-6.859199607	-3.413636882	-1.514387896
C	-7.708179395	-2.296866993	-0.856580103
C	-7.193696307	-0.867144598	-1.069458213
C	-6.12458652	-0.398731563	-0.079216873
N	-5.604027569	0.933215232	-0.387570156
C	-4.952566135	1.526342216	0.765908962
H	-4.618641722	2.549424772	0.530141619
H	-4.056585896	0.95631913	1.105090566
H	-5.652730799	1.586587105	1.611679441
C	-4.708215062	0.920194835	-1.531229448
H	-4.379772214	1.945148944	-1.768070964
H	-5.211495083	0.520770363	-2.423915265
H	-3.794453073	0.30524087	-1.356584862
H	-5.296680371	-1.133782897	-0.013300214
H	-6.578400823	-0.357379879	0.92331872
H	-8.044821401	-0.170851507	-0.988890704
H	-6.831520549	-0.775456358	-2.103922931
H	-7.808380065	-2.50797216	0.219982847
H	-8.718706972	-2.381023458	-1.287562141
O	-6.655926566	-3.07414277	-2.901832027
C	-5.285958829	-2.803158907	-3.205747534
H	-5.167584124	-1.75138772	-3.52612892
H	-4.959634794	-3.445012573	-4.044674572
C	-5.446517251	-3.499830391	-0.963087845
C	-5.004965213	-3.866836651	0.307738819
C	-3.640541489	-3.783420599	0.59399224
H	-3.283640128	-4.070550662	1.58535078
H	-5.70694835	-4.206224158	1.071818522
C	-4.538099916	-3.098902064	-1.939252597
C	-3.176875014	-2.993432365	-1.652076754
H	-2.470958312	-2.645431252	-2.411696991
C	-2.71987635	-3.330430104	-0.369792859
C	-1.247301743	-3.217887669	-0.024958396
H	-0.728220132	-2.623560611	-0.792345239
H	-0.780944375	-4.214528609	-0.006845231
N	-1.025726847	-2.632752384	1.284817692
H	-1.318864651	-1.670916603	1.427098411
C	-0.688603616	-3.365691083	2.372609777
O	-0.407040796	-4.564225635	2.313706459
C	-0.723934547	-2.629551678	3.698475794
H	-0.987069693	-1.57174479	3.547700694
H	0.286825809	-2.663446377	4.138816425

C	-1.726519934	-3.31347804	4.633345043
H	-1.399489171	-4.347007963	4.825108694
H	-2.705831604	-3.375612222	4.136776876
C	-1.87525273	-2.623195032	5.985747982
H	-0.922167523	-2.647298283	6.5275357
H	-2.608728405	-3.151647393	6.610821613
N	-2.271767199	-1.215444895	5.878130172
C	-3.405760304	-0.722980984	5.212137308
N	-4.337539106	-1.543828602	4.646985073
C	-4.880886054	-1.303633515	3.296763414
C	-6.241441898	-2.000261402	3.326157254
C	-6.039066968	-3.134366506	4.336898075
C	-5.155554826	-2.498277566	5.411789678
H	-4.543823709	-3.245617957	5.939215646
H	-5.760751207	-1.966132312	6.170239087
H	-5.503974117	-3.973473558	3.861926875
H	-6.979597165	-3.52455139	4.752403022
H	-6.547601536	-2.354991737	2.333151897
H	-7.011700771	-1.299094691	3.687735063
H	-4.209498604	-1.753069818	2.543199994
H	-4.96515872	-0.233477059	3.072781911
C	-3.320504452	0.661489509	5.194837693
C	-4.288033137	1.650998167	4.707052681
C	-5.653674803	1.488788802	5.011421096
C	-6.59652173	2.408354791	4.550661131
C	-6.191790857	3.512493623	3.792523477
C	-4.839306289	3.680627218	3.481640655
C	-3.894264586	2.752066029	3.922689625
H	-2.847634415	2.873045062	3.635553402
H	-4.517936062	4.530219752	2.874115781
H	-6.930347921	4.236313842	3.438983348
H	-7.652402036	2.268496502	4.79605757
H	-5.970552436	0.640116924	5.620865576
N	-2.115196035	0.987308739	5.836933899
C	-1.572485677	2.210900648	6.134313773
C	-0.386460567	2.285382526	6.809688545
H	0.021560153	3.276771867	7.001868779
H	-2.129810193	3.086233848	5.807282233
C	-1.490326306	-0.167286937	6.260112903
C	-0.279614187	-0.122586799	6.953440074
H	0.16799721	-1.056194184	7.287286136
C	0.31665173	1.112208949	7.230058788
C	1.606396819	1.207910383	7.925280744
C	2.513492059	0.126899176	7.961628418
C	3.735703525	0.208146246	8.611040905
H	4.395451143	-0.656704213	8.571195829
H	2.277271776	-0.801590357	7.436887516
C	2.010297603	2.386959927	8.587580264
H	1.348676894	3.255138562	8.611696162
C	3.224687029	2.483893803	9.25020124
H	3.464200515	3.422598534	9.746555927
C	4.135539679	1.390888235	9.296326268
N	5.324404915	1.469084414	9.975546431
C	6.259049837	0.34624196	9.992970905
C	7.152133167	0.236249868	8.756983816
H	7.807935451	1.115139377	8.659160359

H	6.558008344	0.15034725	7.83322931
H	7.792479935	-0.658215394	8.832056836
H	5.694333291	-0.588201289	10.13648797
H	6.885711658	0.460275622	10.89017405
C	5.778911923	2.733803523	10.55070349
H	6.551086904	2.489687057	11.29460915
H	4.951885086	3.195291691	11.11432047
C	6.341277546	3.73019992	9.536404549
H	5.60024701	3.982613332	8.761640658
H	7.23423653	3.327578899	9.0338749
H	6.628864791	4.665019557	10.04533837

Table A-5. XYZ coordinates of **PYR-C6-CIT**@SMD(water)

Element	X	Y	Z
C	2.78136411085756	-3.60980996473481	-1.58340652045165
H	3.38921976858627	-3.00828030544096	-2.25908728624353
C	2.80412792413377	-5.00294553499150	-1.69118356481728
H	3.41691493350246	-5.50674342927247	-2.44162565817387
C	2.02671214484997	-5.75489514061128	-0.81648626854825
F	2.04439569442033	-7.09992111801182	-0.91117460017882
C	1.22932510532202	-5.15664843239781	0.15448899983798
H	0.63068706395349	-5.77799246251643	0.82351591821462
C	1.21249526155271	-3.76303379067743	0.24429545487200
H	0.58540244235684	-3.29436890408704	1.00401618570736
C	1.98736910378954	-2.97253399364467	-0.61998654167922
C	2.01608908512542	-1.45889220367279	-0.46294490885356
C	0.63224823558931	-0.84546297657787	-0.15958569297456
C	-0.42078658963807	-1.13543750244254	-1.23242186293321
C	-1.56400840592466	-0.12501017501982	-1.35989885123731
N	-2.59513588107736	-0.13718474568593	-0.31755264819824
C	-2.12827683827613	0.45585664181308	0.93061199168076
H	-1.75529895711018	1.49537903607236	0.79493219762101
H	-2.95305450282603	0.48960098180432	1.65875792769464
H	-1.32488197367722	-0.13922313812165	1.37653599653291
C	-3.76585974648391	0.59449517845152	-0.78599120729844
H	-3.54660413697468	1.66192005701304	-1.01465789702737
H	-4.16902733774301	0.13364118972750	-1.70094354961171
H	-4.55665753599128	0.57683226387391	-0.02007063994518
H	-2.07621643434426	-0.33658530032484	-2.31133169174666
H	-1.12969513702351	0.89681974623645	-1.46757814069583
H	-0.83704382872085	-2.14739985673587	-1.09973602326606
H	0.09156130267659	-1.13497925308152	-2.20848004790985
H	0.78477981854553	0.24235668938879	-0.07604160138968
H	0.30851189919570	-1.18396087907580	0.83298825654886
O	2.53109732332267	-0.86306896160609	-1.67452052239317
C	3.34389934201827	0.27274577791176	-1.36287853947283
C	3.76954125534533	0.05027699538344	0.06136770697156
C	4.72210391509915	0.70846853385558	0.83539270109049
H	5.30865825501906	1.53231158198762	0.42004870711726
H	4.18649141304423	0.31053488595045	-2.07202391002125
H	2.76723964393626	1.21143499954133	-1.47191172962593
C	3.02194677431610	-1.00436598201594	0.58570887893186
C	3.23179744829088	-1.44035428217615	1.89161290899233
H	2.65848530706281	-2.27210623087970	2.30601917640681
C	4.18983123401918	-0.78443299762425	2.67321202291575
H	4.35848608078209	-1.11479705553903	3.70042588495409
C	4.93419130252182	0.29385319411895	2.16034549062907
C	5.93323270269903	1.02957723665942	3.02043229744439
H	5.40922472339687	1.69365617908498	3.72608361418056
H	6.55719429798373	1.68093952801573	2.38559139776785
N	6.76377078133069	0.12102589347485	3.79452313363889
H	6.89436348016261	-0.82308538814059	3.44410458394439
C	7.38875931170255	0.47719680992001	4.93382413328060
O	7.30269070872349	1.62779607734229	5.39438619981141
C	8.17236753713894	-0.61918980107702	5.62598679833349
H	9.10191335220585	-0.16707084852872	6.00410816914573
H	8.45156238637809	-1.39392183393784	4.89604377053300

C	7.38433518622842	-1.26835246101119	6.78249224530400
H	7.95484347884049	-2.14659470938796	7.13075555914964
H	6.43210137719665	-1.65599633391815	6.38304130317960
C	7.10663407431218	-0.33542359731550	7.96394696241916
H	6.35640367475059	-0.80187192948015	8.62460679719377
H	6.63775406477644	0.58805444141798	7.58833557023035
C	8.34915721275758	0.01020140061371	8.79935980895841
H	9.23992081591745	0.08682011568961	8.15139282251686
H	8.55847607070350	-0.81509666297370	9.49917414539583
C	8.19299647288532	1.32503306082655	9.57065891022043
H	8.87731115476084	1.35934007152825	10.43269730889321
H	7.16843217959075	1.40174022704140	9.97310091576168
C	8.46173097752407	2.52256579998525	8.65551810224703
H	8.03680291035871	2.35089808597602	7.66010309717526
H	9.53473166813492	2.66878106591350	8.49582521446747
N	7.87327511816169	3.76977310175793	9.15304872309794
C	8.25327387297075	4.49894976373444	10.28999477865672
N	9.41235746796113	4.28714984192492	10.96095462855253
C	9.58621557404013	4.69792234504892	12.36468009288578
C	10.93310923597590	4.08435467492816	12.73750924539950
C	11.72236613460457	4.21680278180701	11.43168974727968
C	10.68819018908761	3.84032281764317	10.36787763776921
H	10.86696253117121	4.33285510195143	9.40030522374424
H	10.69217474000336	2.74993995023434	10.20901699318752
H	12.61069995735139	3.57093144130372	11.38475204128164
H	12.04739480516815	5.26067745472194	11.29282044071319
H	10.80976982445519	3.02172031709057	13.00424490036753
H	11.40094573858358	4.60234782233522	13.58683383559835
H	8.74349442534974	4.33800854506383	12.97401745579986
H	9.62836639733313	5.79464122211911	12.46166648942144
C	7.20841707458474	5.36516928689586	10.60930439553464
C	7.08999675138439	6.44326944142511	11.60348783721786
C	6.06247112477530	6.42710758574314	12.56567766104343
C	5.95289680275935	7.45619599590709	13.50276965189276
C	6.87134052561646	8.51201036504971	13.49836948014400
C	7.89445060156917	8.53892153834797	12.54549240196394
C	7.99872001976105	7.51706231531397	11.59881851487796
H	8.78747471418191	7.54628400665642	10.84404987709435
H	8.61171191797242	9.36319915585442	12.53346946357312
H	6.78861962024706	9.31335318436697	14.23671522203512
H	5.15340735484303	7.42840506042086	14.24717326147620
H	5.35616509958038	5.59371241163861	12.58436729881179
N	6.18652795627909	5.09853975727064	9.68275985102161
C	4.95558684222268	5.67873111508653	9.54176907905218
C	4.11442563416701	5.25674466776982	8.54696659608560
H	3.13241522192409	5.72365541716378	8.48997678252624
H	4.69884429722003	6.45888038419753	10.25598602931036
C	6.60066419125285	4.13028232872999	8.80058319857655
C	5.77456383680760	3.68168142302824	7.77215134571653
H	6.15306016648459	2.93324495601739	7.07907785412966
C	4.49084589242960	4.22736263465764	7.63129972544332
C	3.56225197788880	3.75823591971990	6.59345996368168
C	3.73159539128761	2.51329630153475	5.94810969120360
C	2.84452522051974	2.05233070604114	4.98619575672494
H	3.02175064791991	1.07218613874846	4.54741821573048
H	4.56579089697283	1.86464304518443	6.22158644295552

C	2.44488622769391	4.52326921999990	6.19523013395034
H	2.27430213845492	5.50909980668095	6.63167243739035
C	1.55274078952272	4.08449709059784	5.22804610664087
H	0.72817899721708	4.73918571211297	4.94976882607224
C	1.71154049360522	2.82110186823606	4.59369412526904
N	0.82127100389378	2.36590607005720	3.65930644005523
C	1.03275088741225	1.11518771553059	2.93685311702552
C	0.47461578364601	-0.10624457931507	3.66191713647517
H	-0.59992762166567	0.02172694327842	3.86950687466540
H	0.59581571420164	-1.01000500607888	3.04673784728273
H	0.99128346779596	-0.27461149774136	4.62021598111699
H	2.10280093620756	0.98647667890014	2.72713324015564
H	0.54710170039903	1.21965003600959	1.95731634789193
C	-0.41235466397703	3.08013873279442	3.34510169724760
H	-0.79430952576010	3.57336851828240	4.25102187324882
H	-1.16799327532889	2.32706334386384	3.07259586828392
C	-0.26217005363425	4.08858261091526	2.20985027199725
H	0.46034852618634	4.87834559614542	2.47064733768048
H	0.09032514209273	3.59300693517492	1.29060288626274
H	-1.23045894471901	4.56623631623665	1.98948425648718

Table A-6. XYZ coordinates of **PYR-C6-CIT**@SMD(n-octanol)

Element	X	Y	Z
C	2.89058349449147	-3.63787480837202	-1.36362004780587
H	3.51907371853024	-3.05739990116636	-2.03961595441360
C	2.93241553568312	-5.03363402834144	-1.40733784181489
H	3.58256291807043	-5.56293246816765	-2.10762596571878
C	2.12426095721115	-5.75602198551345	-0.53502648792819
F	2.15836000297759	-7.10294101768280	-0.56934188589736
C	1.27861152685452	-5.12409331328964	0.37165643478449
H	0.65525956658406	-5.72219543498957	1.03982584275288
C	1.24445163940415	-3.72791467978488	0.39826589206693
H	0.57675300462560	-3.23485560145896	1.10634767194301
C	2.04853264510231	-2.96743010089051	-0.46551483660539
C	2.06146772164225	-1.44617531755394	-0.38306444710017
C	0.66965646356237	-0.83047659452434	-0.12869401073369
C	-0.36308489561310	-1.16419740146519	-1.20840561979587
C	-1.56605720545712	-0.21899386973827	-1.31379265755190
N	-2.66324710464259	-0.45049204561039	-0.37698044199415
C	-2.32624468454642	-0.15755687242414	1.00547430830311
H	-1.96919003997515	0.88803054021976	1.15448861565769
H	-3.21405376335793	-0.29704433737840	1.64239661246486
H	-1.55083532363861	-0.83749307047055	1.37914224768779
C	-3.84599929981686	0.29067764346122	-0.78163700103356
H	-3.70104303544172	1.39629567090751	-0.75813428571531
H	-4.13887523440583	0.01750512490669	-1.80817277856629
H	-4.69078252861485	0.05484912242633	-0.11454953127844
H	-1.98965957054841	-0.33432573877300	-2.32423200563173
H	-1.20452024434453	0.83664741378534	-1.25439624172651
H	-0.72819329415026	-2.19825148311139	-1.09458950959060
H	0.15784020447847	-1.12054649519632	-2.17871839816936
H	0.81217827630101	0.26138530271582	-0.08464006668429
H	0.33584691961906	-1.13168036771088	0.87147985943697
O	2.59073113360848	-0.91309246150827	-1.61537220016166
C	3.43638966145204	0.20924524908152	-1.36074227346768
C	3.83082124502103	0.06591961340851	0.08263216269930
C	4.78674486360495	0.74872883120144	0.83225955047775
H	5.40093097139449	1.53064198803406	0.37667301014688
H	4.29350230884618	0.17302032200483	-2.05396315270764
H	2.89767983072867	1.15993279339005	-1.54330065150024
C	3.04992981152209	-0.93647680782803	0.65735497196053
C	3.22271951987752	-1.29024825884052	1.99456283815196
H	2.62325279656907	-2.08214496829543	2.44937394451364
C	4.18497689139844	-0.61116053562707	2.74989329084315
H	4.33698512308838	-0.88097264229857	3.79802368659581
C	4.96738482311729	0.41157616707206	2.18262251219089
C	5.98390091014468	1.15575862086523	3.01698515160220
H	5.47765937079143	1.80813834990186	3.74447028848006
H	6.58628043717591	1.81307727187165	2.36650189271881
N	6.84267316301451	0.25324328928211	3.76571348943993
H	7.14005187562984	-0.60247723373623	3.30699179804193
C	7.34580087769831	0.53445623282184	4.98616301667982
O	7.08343736379828	1.59046139300556	5.57926093446403
C	8.22946996044421	-0.53592575390855	5.59663719607237
H	9.13399654310701	-0.03249683940279	5.97348513775882
H	8.55082936663305	-1.24571301739269	4.81928544346445

C	7.53441469249364	-1.30764323815537	6.73725607432672
H	8.18075690577669	-2.15950163382335	7.01242232035799
H	6.60019252213824	-1.74462447547576	6.34396015501438
C	7.22574497326441	-0.47280657106211	7.98368291846122
H	6.55530150830609	-1.04800929832009	8.64481450620914
H	6.65416957768526	0.41702677541519	7.67840654075150
C	8.46585541831926	-0.04294742082469	8.78694084908344
H	9.33604774555870	0.08049825982415	8.11813321929628
H	8.74497506050519	-0.84457628961217	9.49030655820899
C	8.24944889240521	1.26861317579174	9.55043654353050
H	8.97347944459835	1.37057696106349	10.37404606405868
H	7.24590611869445	1.27123327685043	10.00963446359623
C	8.37334397073903	2.46800804052197	8.60449349213202
H	7.87806822233878	2.25702893983610	7.64926335365299
H	9.42371665199368	2.66801311699367	8.36021123415906
N	7.76646049563797	3.68904012118698	9.13789908144975
C	8.17198115739870	4.40098365339725	10.27416844633392
N	9.33230614045211	4.13033277180195	10.92935369085787
C	9.49113420212968	4.36141188076191	12.37441393620722
C	10.89572458486118	3.82821809582718	12.65139663390419
C	11.64790705081218	4.20875431838424	11.37180454753916
C	10.63176904408306	3.88948764510450	10.27092329227193
H	10.74489804681282	4.52577973819497	9.37953235187750
H	10.73480194699712	2.83688401125212	9.95781377138470
H	12.59007264527692	3.65840361630078	11.23401558159462
H	11.87812643363917	5.28697211577384	11.37652094360067
H	10.87386839554781	2.73200895579929	12.77072687475864
H	11.33188949314119	4.26433974724559	13.56191191790274
H	8.69745983007346	3.84243624419012	12.93412807868279
H	9.43622231771556	5.43304988430292	12.62816212600656
C	7.15808157110687	5.29614122652632	10.60078828921163
C	7.08724458954767	6.35583463138978	11.61745998354525
C	6.02812423358550	6.40466163343942	12.54367827865955
C	5.97237128624924	7.41720900698851	13.50338912339309
C	6.97646006764179	8.38977050188776	13.55925332562331
C	8.03462790963194	8.34855716566153	12.64590865993703
C	8.08727020440462	7.34402791869303	11.67722339272754
H	8.90575334356899	7.32199720124736	10.95409137004471
H	8.82050264258832	9.10744131418159	12.68200175658786
H	6.93416324176078	9.17895356852428	14.31440363032820
H	5.14621019056448	7.44047801929173	14.21872223574315
H	5.25394681122699	5.63373406546713	12.52033158839276
N	6.13386464687075	5.08038449214429	9.66245620370558
C	4.93040698199360	5.71468198399049	9.51079116705728
C	4.08970874788581	5.34593693208726	8.49543603948072
H	3.12903646304778	5.85502487643370	8.42945150054632
H	4.69563407887482	6.49559172892076	10.23195308522184
C	6.52106073954843	4.10911892883394	8.76825045408292
C	5.69419922384740	3.71462992872048	7.71640523590788
H	6.05680748213221	2.96781802304842	7.01184468052510
C	4.43743881312997	4.31557784656832	7.56756045569768
C	3.50637186864138	3.89880399854111	6.51021826050873
C	3.62995643164617	2.64907480326396	5.86425689853868
C	2.74173973091073	2.23164544290380	4.88486436551376
H	2.88467955004525	1.24648842168041	4.44567676962270
H	4.42881353249271	1.96342436210863	6.15068180084174

C	2.43449654669850	4.71609013786850	6.09259238079675
H	2.30232530223009	5.70858403615046	6.52805413248189
C	1.54147385004753	4.32057738606788	5.10740424347851
H	0.75356151869157	5.01387952182778	4.81698588090552
C	1.65194336809658	3.05116402754848	4.47394847773192
N	0.75718846426785	2.63479756412376	3.52566279118693
C	0.90710536596992	1.35468395207927	2.83989713049100
C	0.30539996059174	0.18250634319151	3.61179366281145
H	-0.76701358995594	0.34801605569810	3.80457953385395
H	0.40878462393031	-0.75164097578071	3.03974943254815
H	0.80693261802384	0.04102590244510	4.58239372681042
H	1.96896720141143	1.17078329307429	2.62355999038884
H	0.41495871815395	1.44700134056243	1.86146963623013
C	-0.41931732541027	3.42332787944207	3.17336300422466
H	-0.81191843908012	3.92455549790372	4.07124121063203
H	-1.20465574806936	2.71987179983111	2.85539982427880
C	-0.15936391039196	4.43962324958530	2.06389299411420
H	0.60985283358101	5.17010328402759	2.36157626402026
H	0.18576662813737	3.93801621351265	1.14478119821180
H	-1.08222307851694	4.99267756183786	1.82327685833649

3. Chemical synthesis and characterization

Chemical synthesis

Unless otherwise stated, all glassware/reaction vessels were flame-dried before use and all reactions were performed under argon atmosphere. Trifluoromethanesulfonic anhydride (Tf_2O) was distilled over P_4O_{10} prior to use, and stored under argon atmosphere at 4 °C. All other reagents were used as received from commercial suppliers and stored under argon atmosphere, unless otherwise stated. The progress of reactions was monitored by thin layer chromatography (TLC) performed on aluminum plates (0.2 mm thickness) coated with silica gel F_{254} or with ^1H NMR spectroscopy. TLC chromatograms were visualized by fluorescence quenching with UV irradiation at 254 nm (or 366 nm) and/or by staining using either potassium permanganate or phosphomolybdic acid. Flash column chromatography was performed using silica gel 60 (230–400 mesh, Merck and co.).

All ^1H, ^{13}C, and ^{19}F NMR spectra were recorded using a Bruker AV-400, AV-500, or AV-600 spectrometer at 300 K. Chemical shifts are given in parts per million (ppm, δ), referenced to the solvent peak of $CDCl_3$, defined at δ = 7.26 ppm (^1H NMR) and δ = 77.16 ppm (^{13}C NMR). Coupling constants (*J*) are quoted in Hz. ^1H or ^{13}C NMR splitting patterns were designated as singlet (s), doublet (d), triplet (t), quartet (q), pentet (p). Splitting patterns that could not be interpreted or easily visualized were designated as multiplet (m) or broad (br). Protons and carbons were assigned whenever unambiguously possible.

IR (infrared) spectra were recorded using a Perkin-Elmer Spectrum 100 FT-IR spectrometer. Wavenumbers (ν_{max}) are reported in cm^{-1}. Mass spectra were obtained using a Finnigan MAT 8200 (70 eV) or MAT 8400 (70 eV) using electrospray ionization (ESI). High resolution mass spectra were recorded on a Bruker APEX III FT-MS (7 T magnet).

Photophysical properties

Ultraviolet absorbance of PyrAtes was measured on a Thermo Fisher Scientific G10S spectrophotometer at concentrations of 1 µM, 5 µM, 10 µM and 20 µM in PBS:DMSO 99:1 v/v. Fluorescence was recorded on a SHIMADZU RF-6000 spectrofluorometer at concentration of 2 µM in PBS:DMSO 99:1 v/v. The fluorescence quantum yield is calculated relatively to a known standard by the spectrofluorometer (coumarin 153, C153; with Φ_f = 0.53 in EtOH).[32] The refractive index correction for measurements in different solvents was taken into account (1.33 for aqueous media).

Table S5. *Photophysical properties of PyrAte-(S)-citalopram conjugates in aqueous media (PBS:DMSO 99:1 v/v). C153 (coumarin 153) was used as internal standard for the measurement of quantum yield.*

Compound	λ_{max} (nm)	λ_{Em} (nm)	Stokes shift (nm)	ε at λ_{max} (M^{-1} cm^{-1})	Quantum yield	Brightness (at λ_{max}, 10^3 M^{-1} cm^{-1})
PYR-C3-CIT	405	493	88	30000	0.82	24.0
PYR-C6-CIT	406	493	87	39600	0.69	27.0

Experimental Procedures

3.1. Reduction of citalopram and (S)-citalopram

3-(5-(Aminomethyl)-1-(4-fluorophenyl)-1,3-dihydroisobenzofuran-1-yl)-*N,N*-dimethylpropan-1-amine (5)

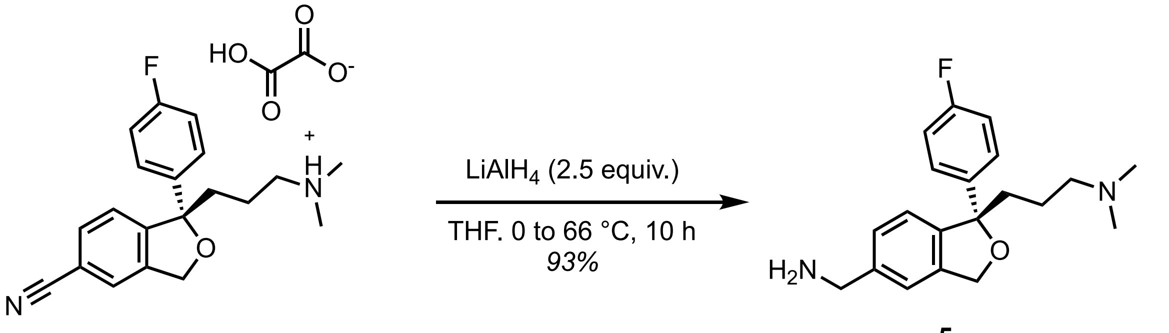

(*S*)-citalopram oxalate   **5**

To a cooled (0 °C) suspension of (*S*)-citalopram oxalate (249 mg, 0.6 mmol, 1.00 equiv.) in anhydrous THF (0.1 M), lithium aluminium hydride ($LiAlH_4$, 1 M in THF, 1.5 mL, 1.5 mmol, 2.50 equiv.) was added dropwise. The reaction mixture was heated to reflux (66 °C) for 5 hours, after which time aqueous solution of NaOH (0.1 M) was carefully added. The reaction mixture was filtered, diluted with water, and extracted with ethyl acetate. The combined organic phases were dried over anhydrous magnesium sulfate, filtered, and concentrated to give the desired primary amine **5**.

Yield: quant., 134 mg, yellow oil. Data are in accordance to the literature.[84]

^{1}H NMR (600 MHz, $CDCl_3$): δ 7.45 (dd*, J* = 8.9, 5.4 Hz, 2H), 7.22 (s, 2H), 7.16 (s, 1H), 6.97 (t*, J* = 8.8 Hz, 2H), 5.13 (d*, J* = 4.1 Hz, 2H), 3.86 (s, 2H), 2.24 – 2.15 (m, 4H), 2.13 (s, 6H), 1.54 – 1.42 (m, 1H), 1.39 – 1.29 (m, 1H) ppm. Amine protons could not be detected.

3.2. General Procedures

3.2.1. General Procedure A — Synthesis of azides

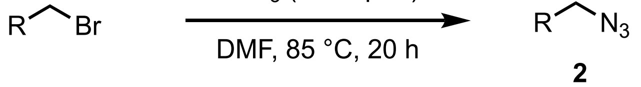

2

NaN_3 (2.5 equiv.) was added to the stirred solution of alkyl bromide (1.0 equiv.) in DMF at room temperature, and the mixture was subsequently heated to 80 °C for 20 h. Then, the mixture was cooled to room temperature and the salt was filtered off. The filter cake was washed wish Et_2O, the filtrate was

diluted with water, extracted with Et_2O (3 times) and the collected organic phases were washed with brine (6 times), dried over anhydrous magnesium sulfate and concentrated to give a crude oil. Purification by column chromatography (silica gel, EtOAc in heptanes) afforded the desired azides **2**.

3.2.2. General Procedure B — Synthesis of PyrAtes

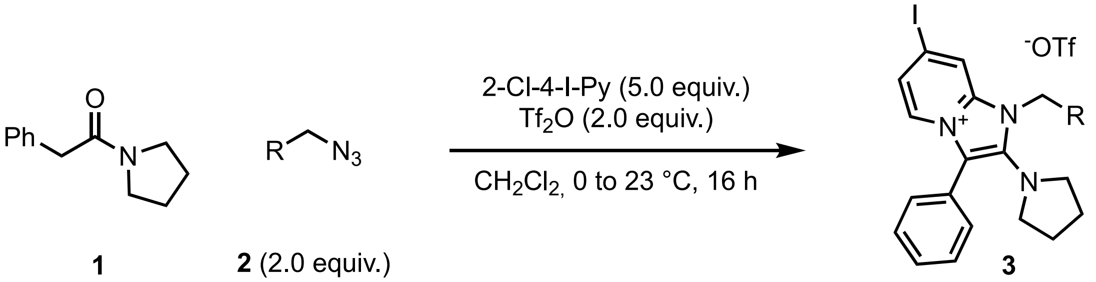

1 **2** (2.0 equiv.) **3**

Triflic anhydride (2.0 equiv.) was added dropwise to a mixture of the 2-phenyl-1-(pyrrolidin-1-yl)ethan-1-one **1** (1.0 equiv., typical scale: 0.2 mmol) and 2-Cl-4-I-pyridine (5.0 equiv.) in CH_2Cl_2 at 0 °C. After 15 min, the azide **2** (2.0 equiv.) was added, and the reaction mixture was allowed to reach room temperature. After stirring for 16 h, $NaHCO_3$ (aq.) was added to the reaction mixture, which was then extracted with CH_2Cl_2. The organic phase was dried over anhydrous magnesium sulfate, concentrated and the resulting brown oil was purified by column chromatography (silica gel, DMA in CH_2Cl_2 0% to 50%) affording the corresponding iodo-PyrAte **3**.

3.2.3. General Procedure C — Suzuki-Miyaura Cross-Couplings

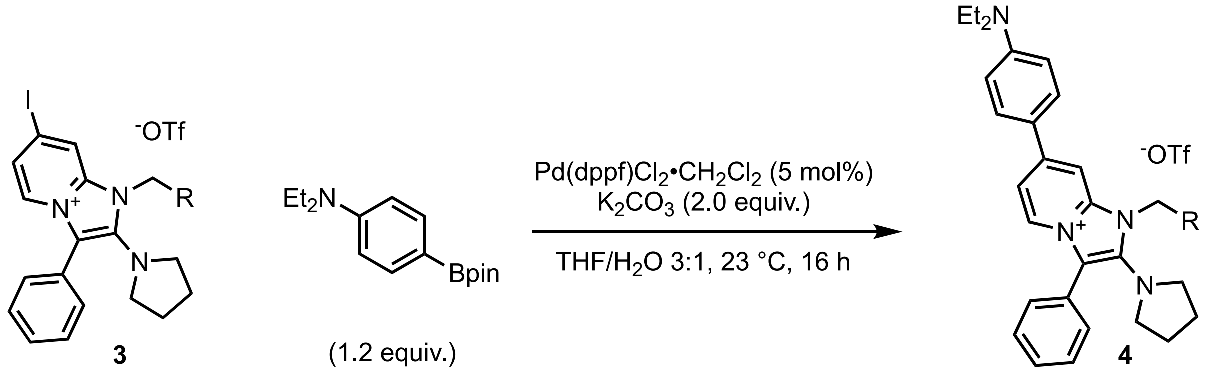

3 (1.2 equiv.) **4**

N,N-diethyl-4-(4,4,5,5-tetramethyl-1,3,2-dioxaborolan-2-yl)aniline (1.2 equiv.), [1,1′-bis(diphenylphosphino)ferrocene]dichloropalladium(II) complex with dichloromethane (0.05 equiv.) and K_2CO_3 (2.0 equiv.) were added in a Schlenk tube under argon atmosphere. Iodo-PyrAte **3** (1.0 equiv.) dissolved in THF (0.1 M) was added to the mixture, followed by water (0.033 M). The mixture was stirred at room temperature for 16 h, before being diluted and extracted with CH_2Cl_2. The organic phase was dried over magnesium sulfate, concentrated and the remaining mixture was purified by column

chromatography (silica gel, DMA [CH_2Cl_2/MeOH/NH_4OH 90:10:1] in CH_2Cl_2 0% to 60%) to afford the desired cross-coupling product **4**.

3.3. Characterization Data

Methyl 7-azidoheptanoate (2a)

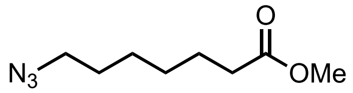

Prepared according to **General Procedure A**. Data are in accordance with literature.[85]

Yield: 1.75 g, 95%.

Methyl 4-azidobutanoate (2b)

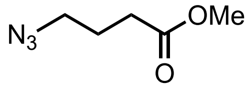

Prepared according to **General Procedure A**. Data are in accordance with literature. 33 [86]

Yield: 1.4 g, 92%.

7-Iodo-1-(7-methoxy-7-oxoheptyl)-3-phenyl-2-(pyrrolidin-1-yl)-1H-imidazo[1,2-a]pyridin-4-ium trifluoromethanesulfonate (3a)

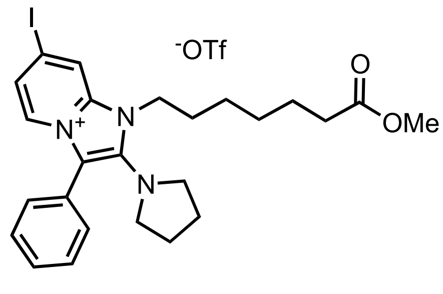

Prepared according to **General Procedure B**.

Yield: 299 mg, 88%, brown oil.

^{1}H NMR (400 MHz, $CDCl_3$): δ 8.17 – 8.15 (m, 1H), 7.66 (d, *J* = 7.1 Hz, 1H), 7.62 – 7.57 (m, 3H), 7.56 – 7.51 (m, 2H), 7.48 (dd, *J* = 7.1, 1.5 Hz, 1H), 4.36 (t, *J* = 7.9 Hz, 2H), 3.67 (s, 3H), 3.23 – 3.17 (m, 4H), 2.33 (t, *J* = 7.3 Hz, 2H), 1.94 – 1.80 (m, 6H), 1.68 – 1.61 (m, 2H), 1.50 – 1.39 (m, 4H) ppm.

^{13}C NMR (100 MHz, $CDCl_3$): δ 174.2, 140.8, 136.2, 131.8 (2C), 131.3, 130.1 (2C), 126.6, 125.0, 124.7, 120.8 (q, *J* = 320.8 Hz, OTf), 119.3, 114.9, 97.5, 52.5 (2C), 51.7, 44.9, 33.9, 28.9, 28.6, 26.6, 25.9 (2C), 24.7 ppm.

^{19}F NMR (565 MHz, CDCl$_3$): δ –78.2 ppm.

IR (neat) ν_{max}: 2950, 2860, 1730, 1636, 1274, 1028, 635 cm^{-1}.

HRMS (ESI$^+$): calculated for [M–TfO$^-$]$^+$ ($C_{25}H_{31}IN_3O_2^+$) requires m/z 532.1455, found m/z 532.1469.

7-Iodo-1-(4-methoxy-4-oxobutyl)-3-phenyl-2-(pyrrolidin-1-yl)-1*H*-imidazo[1,2-a]pyridin-4-ium trifluoromethanesulfonate (3b)

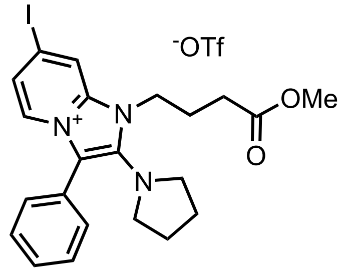

Prepared according to **General Procedure B**.

Yield: 410 mg, 64%, brown powder.

^{1}H NMR (600 MHz, CDCl$_3$): δ 8.36 (d, *J* = 0.7 Hz, 1H), 7.68 (d, *J* = 7.1 Hz, 1H), 7.57 (dd, *J* = 4.1, 2.3 Hz, 3H), 7.55 – 7.51 (m, 2H), 7.49 (d, *J* = 7.0 Hz, 1H), 4.48 – 4.35 (m, 2H), 3.66 (s, 3H), 3.18 (dd, *J* = 7.7, 5.5 Hz, 4H), 2.53 (t, *J* = 6.5 Hz, 2H), 2.20 – 2.12 (m, 2H), 1.88 – 1.77 (m, 4H) ppm.

^{13}C NMR (151 MHz, CDCl$_3$): δ 173.2, 140.8, 136.3, 131.7 (2C), 131.3, 130.0 (2C), 126.7, 124.9, 124.6, 120.8 (q, *J* = 321.0 Hz, OTf), 119.5, 114.8, 98.0, 52.4 (2C), 52.0, 43.9, 30.4, 25.8 (2C), 24.0 ppm.

^{19}F NMR (565 MHz, CDCl$_3$): δ –78.2 ppm.

IR (neat) ν_{max}: 1730, 1638, 1444, 1261, 1223, 1152, 1030, 637 cm^{-1}.

HRMS (ESI$^+$): exact mass calculated for [M–TfO$^-$]$^+$ ($C_{22}H_{25}IN_3O_2^+$) requires *m/z* 490.0986, found *m/z* 490.0987.

7-(4-(diethylamino)phenyl)-1-(7-methoxy-7-oxoheptyl)-3-phenyl-2-(pyrrolidin-1-yl)-1*H*-imidazo[1,2-*a*]pyridin-4-ium trifluoromethanesulfonate (4a)

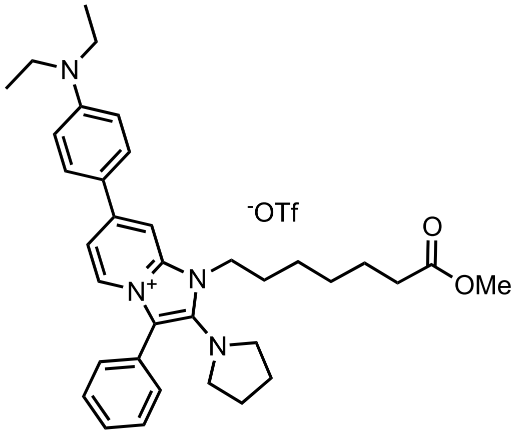

Prepared according to **General Procedure C**.

Yield: 193 mg, 67%, brown foam.

^{1}H NMR (400 MHz, CDCl$_3$): δ 7.93 (s, 1H), 7.88 (d, *J* = 7.2 Hz, 1H), 7.75 (d, *J* = 9.0 Hz, 2H), 7.66–7.56 (m, 3H), 7.55–7.45 (m, 3H), 6.77 (d, *J* = 7.2 Hz, 2H), 4.52 (t, *J* = 7.4 Hz, 2H), 3.65 (s, 3H), 3.43 (q, *J* = 7.1 Hz, 4H), 3.25–3.09 (m, 4H), 2.31 (t, *J* = 7.3 Hz, 2H), 1.90–1.79 (m, 6H), 1.70–1.58 (m, 2H), 1.54–1.35 (m, 4H), 1.21 (t, *J* = 7.1 Hz, 6H) ppm.

^{13}C NMR (100 MHz, CDCl$_3$): δ 174.3, 149.6, 146.0, 140.4, 137.4, 131.6 (2C), 131.0, 130.0 (2C), 129.0 (2C), 125.3, 124.6, 121.5, 115.8, 114.2, 112.1 (2C), 103.9, 52.7 (2C), 51.6, 44.7 (3C), 34.0, 29.1, 28.8, 26.7, 25.9 (2C), 24.7, 12.7 (2C) ppm.

IR (neat) ν_{max}: 2970, 2936, 2869, 1734, 1649, 1599, 1536, 1466, 1262, 1213, 1155, 1031, 637 cm^{-1}.

HRMS (ESI$^+$): exact mass calculated for [M–TfO$^-$]$^+$ ($C_{22}H_{25}IN_3O_2^+$) requires *m/z* 553.3537, found *m/z* 553.3543.

7-(4-(Diethylamino)phenyl)-1-(4-methoxy-4-oxobutyl)-3-phenyl-2-(pyrrolidin-1-yl)-1H-imidazo[1,2-a]pyridin-4-ium trifluoromethanesulfonate (4b)

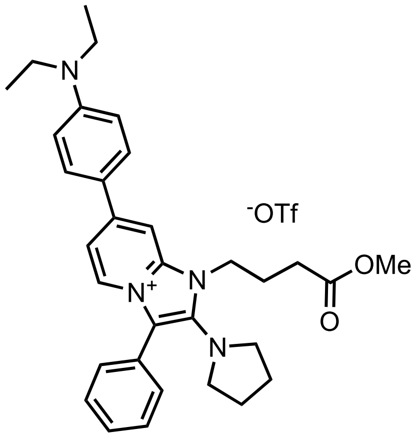

Prepared according to **General Procedure C**.

Yield: 180 mg, 88%, brown oil.

^{1}H NMR (600 MHz, CDCl$_3$): δ 8.16 (s, 1H), 7.88 (d, *J* = 7.2 Hz, 1H), 7.80 (d, *J* = 8.9 Hz, 2H), 7.65 – 7.60 (m, 3H), 7.52 (d, *J* = 7.6 Hz, 2H), 7.49 (d, *J* = 7.0 Hz, 1H), 6.78 (d, *J* = 8.9 Hz, 2H), 4.66 – 4.56 (m, 2H), 3.69 (s, 3H), 3.44 (q, *J* = 7.0 Hz, 4H), 3.21 (t, *J* = 6.4 Hz, 4H), 2.65 (t, *J* = 6.6 Hz, 2H), 2.29 – 2.21 (m, 2H), 1.88 (t, *J* = 6.7 Hz, 4H), 1.22 (t, *J* = 7.1 Hz, 6H) ppm.

^{13}C NMR (151 MHz, CDCl$_3$): δ 173.5, 149.6, 146.0, 140.3, 137.6, 131.6 (2C), 131.0, 130.0 (2C), 128.9 (2C), 125.2, 124.5, 121.3, 115.7, 114.1, 112.0 (2C), 103.8, 52.6 (2C), 51.9, 44.6 (2C), 43.6, 30.6, 25.8 (2C), 24.2, 12.7 ppm. Triflate carbon was not detectable by NMR spectroscopy.

^{19}F NMR (565 MHz, CDCl$_3$): δ –78.2 ppm.

IR (neat) v_{max}: 2972, 2933, 2872, 1733, 1645, 1536, 1467, 1446, 1438, 1408, 1376, 1356, 1315, 1263, 1212, 1156, 1115, 1078, 1031, 808, 637 cm^{-1}.

HRMS (ESI$^+$): exact mass calculated for $[M–TfO^-]^+$ ($C_{32}H_{39}N_4O_2^+$) requires *m/z* 511.3068, found *m/z* 511.3087.

(*S*)-7-(4-(diethylamino)phenyl)-1-(7-(((1-(3-(dimethylamino)propyl)-1-(4-fluorophenyl)-1,3-dihydroisobenzofuran-5-yl)methyl)amino)-7-oxoheptyl)-3-phenyl-2-(pyrrolidin-1-yl)-1*H*-imidazo[1,2-*a*]pyridin-4-ium trifluoromethanesulfonate (PYR-C6-CIT)

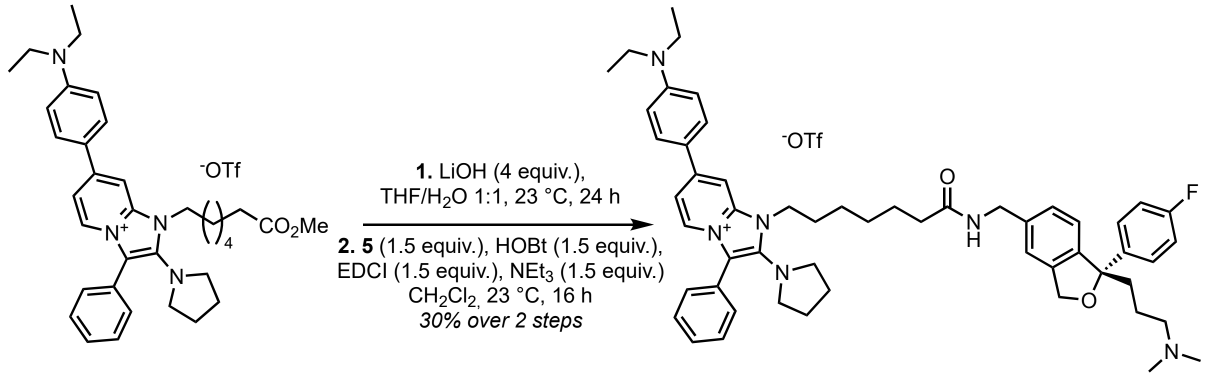

Methyl ester Pyrate **4a** (35.1 mg, 0.05 mmol, 1.0 equiv.) was dissolved in a mixture of THF/H_2O 1:1 (v/v, 0.1 M). LiOH monohydrate (8.4 mg, 0.2 mmol, 4.0 equiv.) was added, and the resulting mixture was stirred at room temperature for 24 h. Then, the mixture was acidified by addition of aqueous solution of HCl (1 N) to pH = 2-3. The aqueous phase was subsequently extracted with CH_2Cl_2 (3 times), the combined organic layers were dried over magnesium sulfate, filtered and concentration under reduced pressure to afford quantitatively the desired pure carboxylic acid, which was directly used at the next step without further purification.

The crude carboxylic acid described above was dissolved in 0.5 mL DCM and HOBt (10.1 mg, 0.075 mmol, 1.5 equiv. based on the methyl ester above), EDCI (14.4 mg, 0.075 mmol, 1.5 equiv.), triethylamine (10.5 µL, 0.075 mmol, 1.5 equiv.), and reduced (*S*)-citalopram (**5**) (24.6 mg, 0.075 mmol, 1.5 equiv.) were added under argon flow. The mixture was stirred for 16 h at room temperature before EtOAc was added, and the organic layer was washed with saturated aqueous solution of ammonium chloride and then saturated aqueous solution of sodium bicarbonate. The organic layer was dried over magnesium sulfate, filtered, and concentrated under reduced pressure The resulting material was purified with column chromatography (silica gel, 0% DMA to 100% DMA in DCM (DMA = DCM:MeOH:NH_4OH (aq., 25%) = 90:10:1) to afford the desired **PYR-C6-CIT** conjugate.

Yield: 30 mg, 60%, brown foam.

^{1}H NMR (600 MHz, CDCl$_3$): δ 7.99 (s, 1H), 7.86 (d, *J* = 7.2 Hz, 1H), 7.76 (d, *J* = 8.8 Hz, 2H), 7.64–7.60 (m, 3H), 7.49–7.38 (m, 6H), 7.27–7.25 (m, 1H), 7.20 (s, 1H), 7.17 (d, *J* = 7.8 Hz, 1H), 6.95–6.90 (m, 2H) 6.76 (d, *J* = 7.2 Hz, 2H), 5.09 (d, *J* = 12.4 Hz, 1H), 5.03 (d, *J* = 12.4 Hz, 1H), 4.46–4.73 (m, 4H), 3.40 (q, *J* = 7.1 Hz, 4H), 3.19–3.11 (m, 4H), 2.66–2.53 (m, 2H), 2.40 (s, 6H), 2.34 (t, *J* = 7.1 Hz, 2H), 2.16 (t, *J* = 7.5 Hz, 2H), 1.92–1.87 (m, 6H), 1.73–1.67 (m, 2H), 1.56–1.38 (m, 6H), 1.18 (t, *J* = 7.1 Hz, 6H) ppm.

^{13}C NMR (151 MHz, CDCl$_3$): δ 173.8, 161.9 (d, J_{CF} = 244.8 Hz), 149.7, 146.2, 142.1, 141.2, 140.2, 139.4, 139.3, 137.5, 131.4 (2C), 131.2, 130.1 (2C), 128.9 (2C), 127.8, 126.9 (d, J_{CF} = 8.8 Hz, 2C), 125.1, 124.4, 121.9, 121.1, 121.0, 115.4, 115.0 (d, J_{CF} = 21.2 Hz, 2C), 114.1, 112.1 (2C), 103.4, 90.7, 72.0, 58.6, 52.6 (2C), 44.6 (2C), 44.4, 44.0 (2C), 43.2, 38.7, 35.7, 29.1, 28.1, 26.0, 25.9, 25.2 (2C), 20.8, 12.7 (2C) ppm.

^{19}F NMR (565 MHz, CDCl$_3$): δ –78.2, –116.4 ppm.

IR (neat) $ν_{max}$: 2928, 2869, 1649, 1599, 1536, 1466, 1260, 1157, 1030, 834, 637 cm^{-1}.

HRMS (ESI$^+$): exact mass calculated for [M–TfO$^-$]$^+$ ($C_{54}H_{66}FN_6O_2^+$) requires *m/z* 849.5226, found *m/z* 849.5224.

(*S*)-7-(4-(Diethylamino)phenyl)-1-(4-(((1-(3-(dimethylamino)propyl)-1-(4-fluorophenyl)-1,3-dihydroisobenzofuran-5-yl)methyl)amino)-4-oxobutyl)-3-phenyl-2-(pyrrolidin-1-yl)-1*H*-imidazo[1,2-a]pyridin-4-ium trifluoromethanesulfonate (PYR-C3-CIT)

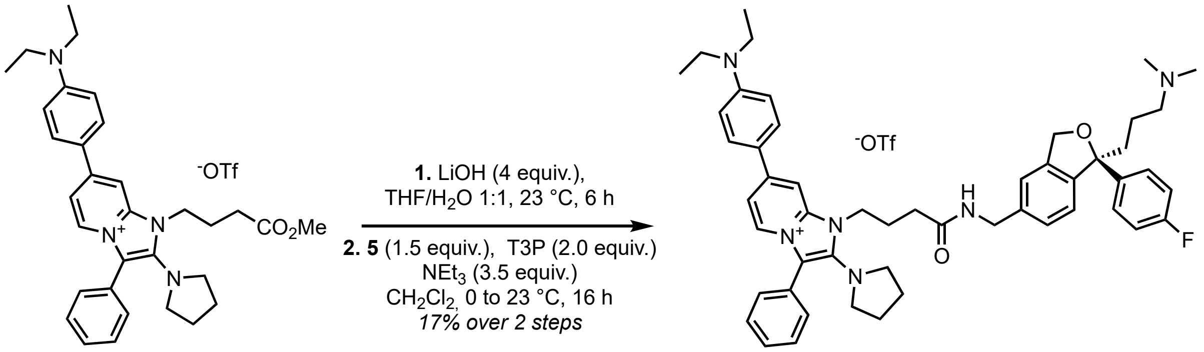

Methyl ester Pyrate **4b** (180 mg, 0.272 mmol, 1.0 equiv.) was dissolved in a mixture of THF/H_2O 3:1 (v/v, 0.1 M). LiOH monohydrate (45.7 mg, 1.09 mmol, 4.0 equiv.) was added, and the resulting mixture was stirred at room temperature for 6 h. Then, the mixture was acidified by addition of aqueous solution of HCl (1 N) to pH = 2-3. The aqueous phase was subsequently extracted with CH_2Cl_2 (3 times), the combined organic layers were dried over magnesium sulfate, filtered and concentration under reduced pressure to afford quantitatively the desired pure carboxylic acid **6b**.

To a mixture of the pyrate-carboxylic acid **6b** (120 mg, 0.186 mmol, 1.0 equiv.), triethylamine (90.5 µL, 0.649 mmol, 3.5 equiv.) and the reduced (*S*)-citalopram (**5**) (91.4 mg, 0.278 mmol ,1.5 equiv.) in CH_2Cl_2 (0.1 M) was slowly added T3P (50% in EtOAc, 221 µL, 0.371 mmol, 2 equiv.) at 0 °C. The reaction mixture was slowly allowed to warm to room temperature and stirred for 16 h. After that period, the reaction was diluted with CH_2Cl_2 and the mixture was washed successively with H_2O, saturated aqueous solution of sodium bicarbonate, and brine. The organic layer was dried over magnesium sulfate, filtered, concentrated under reduced pressure, and purified by column chromatography (silica gel, 0% to 60% DMA [CH_2Cl_2/MeOH/NH_4OH 90:10:1] in CH_2Cl_2) to afford the desired **PYR-C3-CIT** conjugate.

Yield: 30 mg, 17%, bright yellow-green powder.

^{1}H NMR (600 MHz, $CDCl_3$): δ 9.44 (t, *J* = 5.7 Hz, 1H), 8.35 (s, 1H), 7.89 (d, *J* = 9.0 Hz, 2H), 7.80 (d, *J* = 7.2 Hz, 1H), 7.65 – 7.58 (m, 3H), 7.49 – 7.45 (m, 2H), 7.43 (dd, *J* = 7.2, 1.4 Hz, 1H), 7.41 – 7.35 (m, 3H), 7.29 (s, 1H), 7.13 (d, *J* = 7.8 Hz, 1H), 6.90 (t, *J* = 8.7 Hz, 2H), 6.79 (d, *J* = 9.0 Hz, 2H), 5.01 (dd, *J* = 41.3, 12.3 Hz, 2H), 4.58 – 4.46 (m, 2H), 4.43 (d, *J* = 6.1 Hz, 2H), 3.43 (q, *J* = 7.0 Hz, 4H), 3.12 (t, *J* = 6.4 Hz, 4H), 2.85 – 2.74 (m, 2H), 2.45 – 2.33 (m, 2H), 2.32 – 2.17 (m, 8H), 2.15 – 2.05 (m, 2H), 1.83 (t, *J* = 6.5 Hz, 4H), 1.51 (s, 1H), 1.39 (s, 1H), 1.21 (t, *J* = 7.1 Hz, 6H) ppm.

^{13}C NMR (151 MHz, $CDCl_3$): δ 172.9, 161.8 (d, J_{CF} = 244.9 Hz), 149.8, 145.9, 142.2, 141.4, 140.2, 139.7, 139.2, 137.7, 131.5, 131.1, 130.0, 129.0 (2C), 127.8, 127.0 (d, J_{CF} = 8.0 Hz, 2C), 125.3, 124.0, 121.7, 121.0, 120.9, 115.3, 115.0 (d, J_{CF} = 21.2 Hz, 2C), 113.4, 112.1 (2C), 103.8, 90.7, 72.0, 59.2 (*deduced from HSQC*), 52.5 (2C), 44.7 (2C), 44.6 (2C, *deduced from HSQC*), 44.0, 43.0, 39.2, 33.0, 25.9, 25.8 (2C), 21.5 (*deduced from HSQC*), 12.7 (2C) ppm. Triflate carbon was not detectable by NMR spectroscopy.

^{19}F NMR (565 MHz, $CDCl_3$): δ –75.0, –116.7 ppm.

IR (neat) ν_{max}: 3227, 3197, 2950, 2924, 2870, 2854, 1717, 1650, 1599, 1536, 1505, 1466, 1406, 1376, 1354, 1312, 1030, 1012, 833, 811 cm^{-1}.

HRMS (ESI$^+$): exact mass calculated for [M–TfO$^-$]$^+$ ($C_{51}H_{60}FN_6O_2^+$) requires *m/z* 807.4756, found *m/z* 807.4758.

<u>NMR Spectra</u>

7-Iodo-1-(7-methoxy-7-oxoheptyl)-3-phenyl-2-(pyrrolidin-1-yl)-1H-imidazo[1,2-a]pyridin-4-ium trifluoromethanesulfonate (3a)

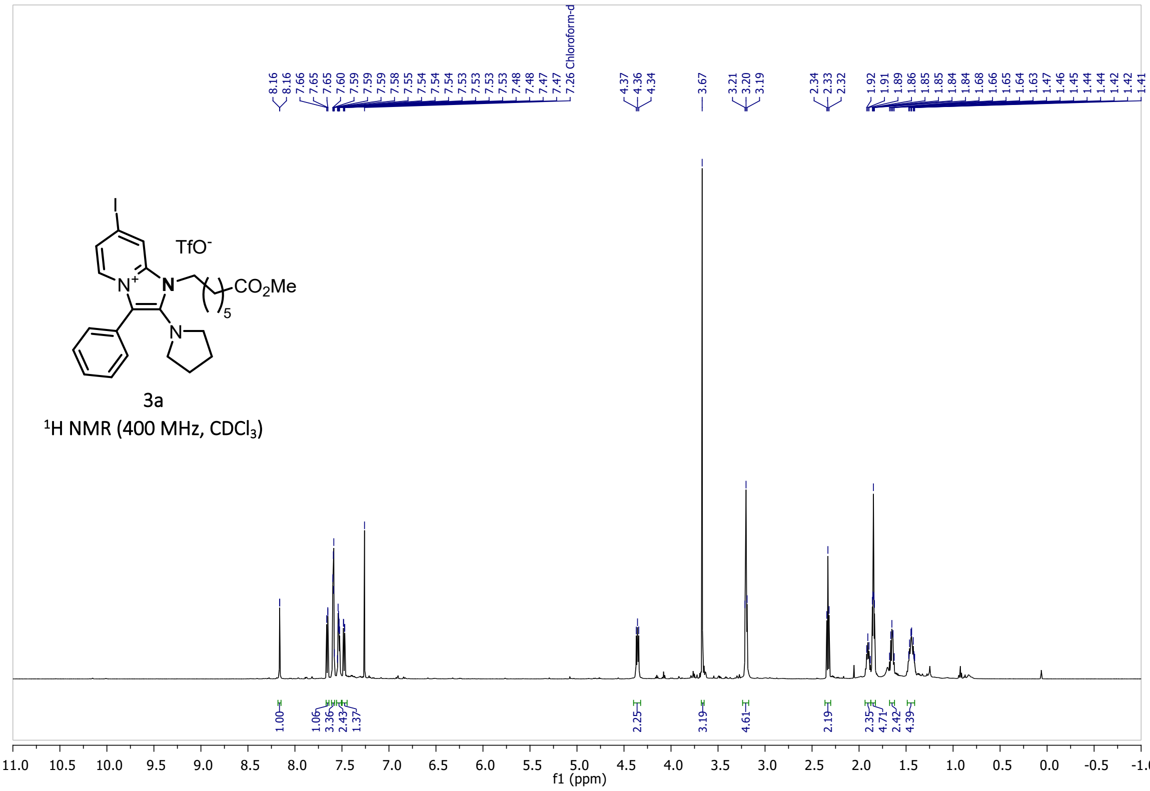

TfO⁻
CO2Me
5
3a
1H NMR (400 MHz, CDCl3)
7.26 Chloroform-d
f1 (ppm)

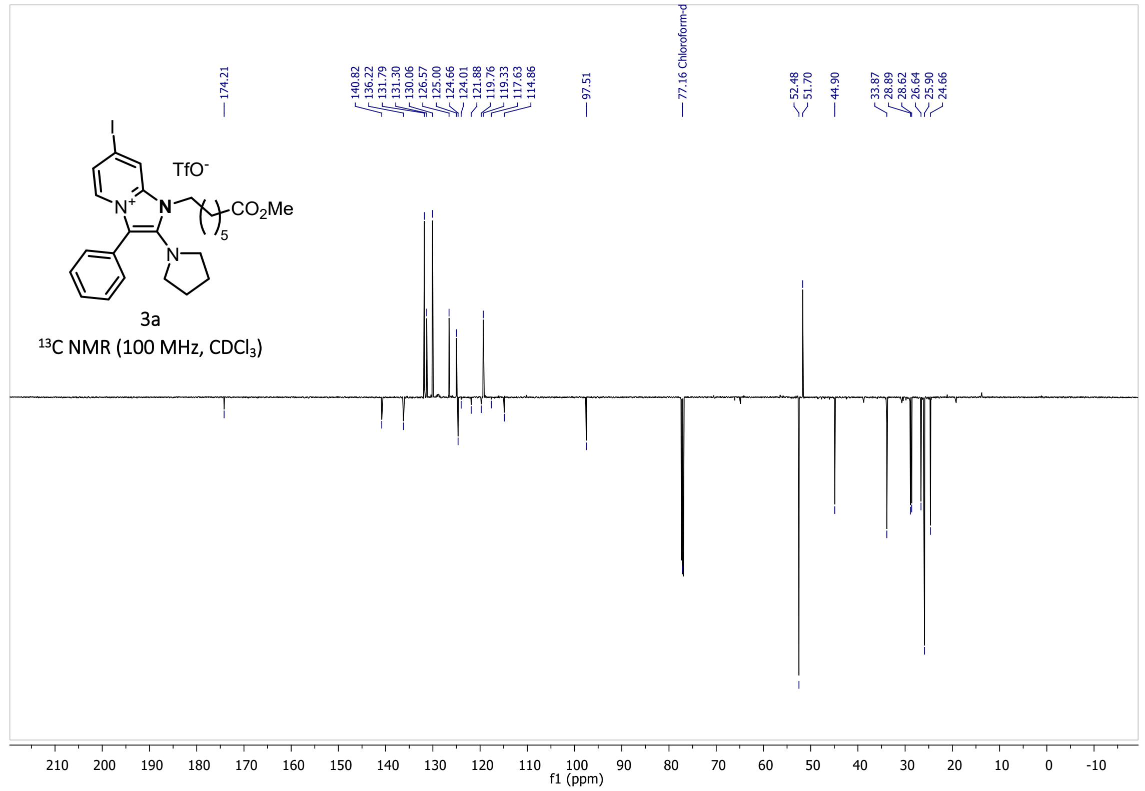

TfO⁻
CO2Me
5
3a
13C NMR (100 MHz, CDCl3)
174.21
140.82
136.22
131.79
131.30
130.06
126.57
125.00
124.66
124.01
121.88
119.76
119.33
117.63
114.86
97.51
77.16 Chloroform-d
52.48
51.70
44.90
33.87
28.89
28.62
26.64
25.90
24.66
f1 (ppm)

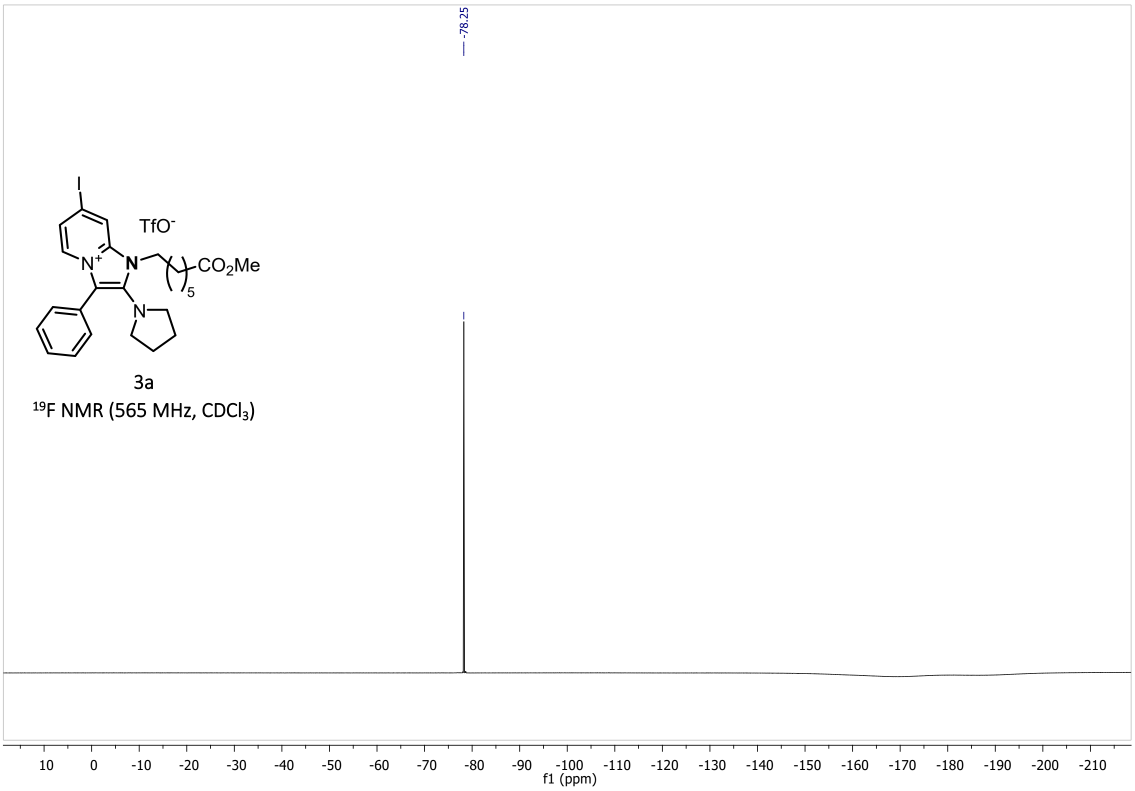

7-Iodo-1-(4-methoxy-4-oxobutyl)-3-phenyl-2-(pyrrolidin-1-yl)-1*H*-imidazo[1,2-a]pyridin-4-ium trifluoromethanesulfonate (3b)

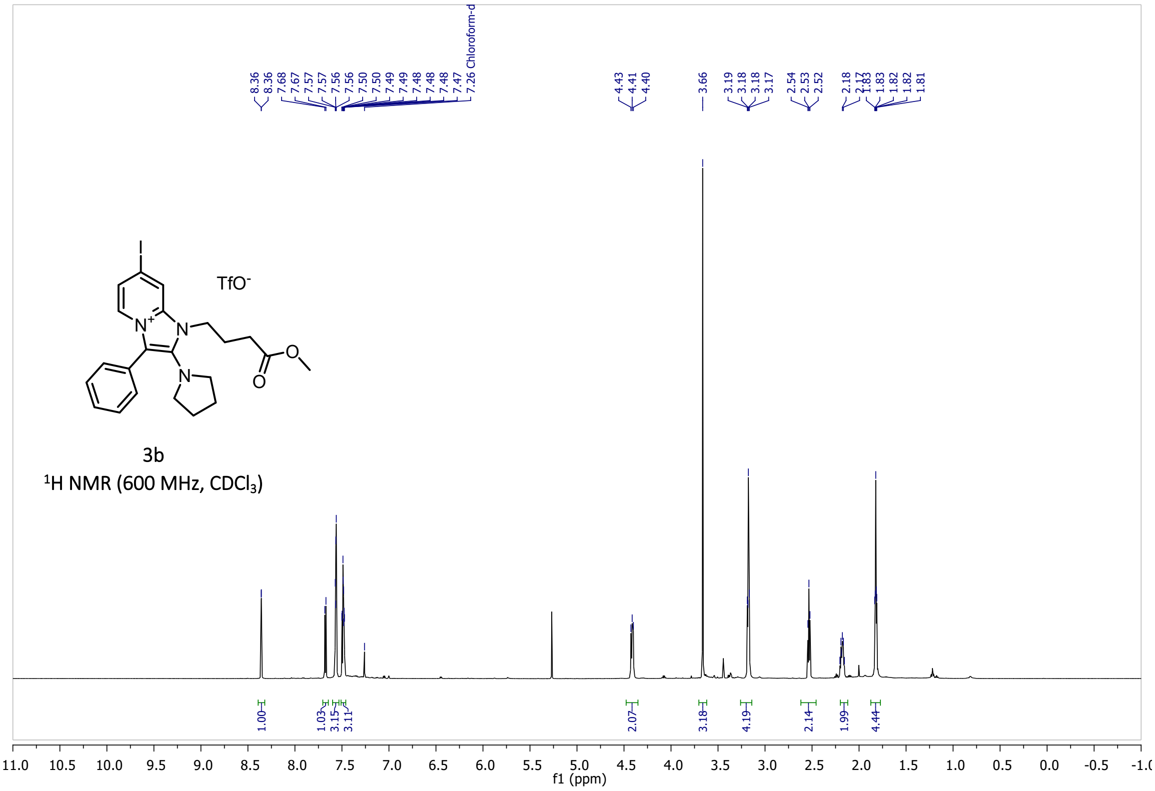

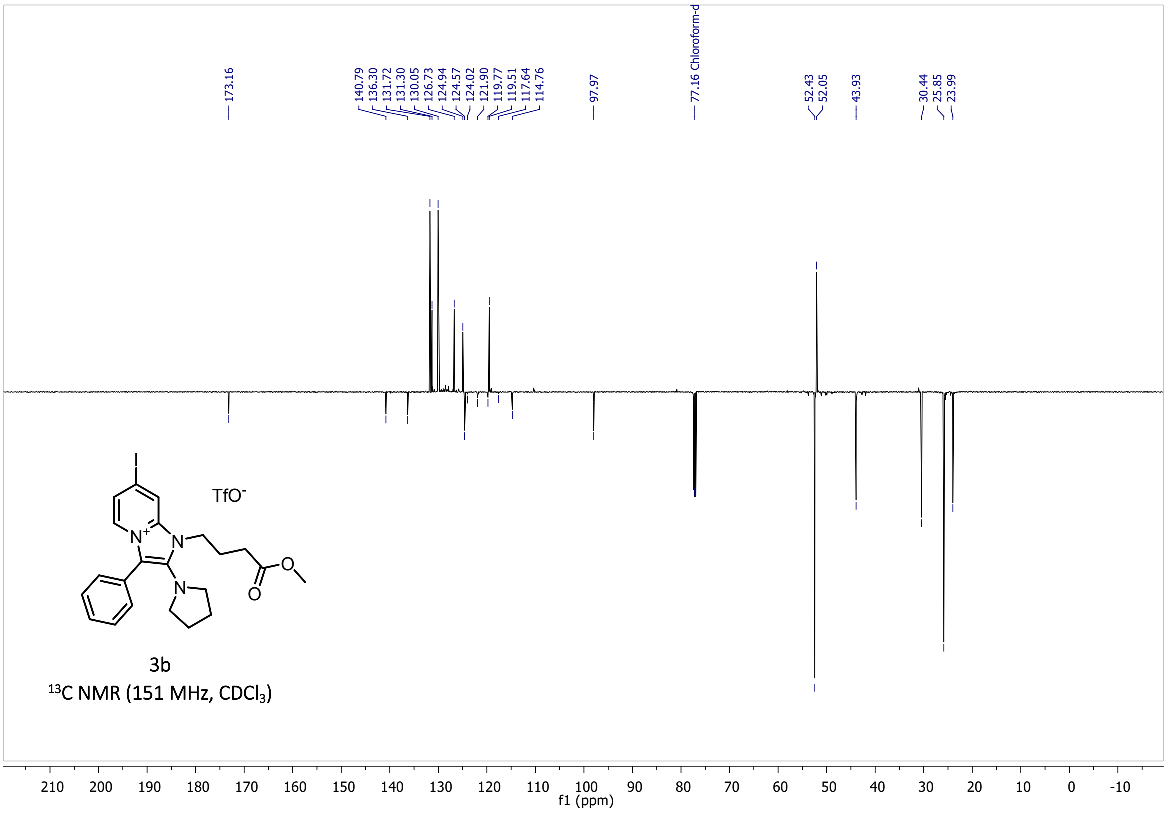
173.16
140.79
136.30
131.72
131.30
130.05
126.73
124.94
124.57
124.02
121.90
119.77
119.51
117.64
114.76
97.97
77.16 Chloroform-d
52.43
52.05
43.93
30.44
25.85
23.99
TfO-
3b
13C NMR (151 MHz, CDCl3)
f1 (ppm)

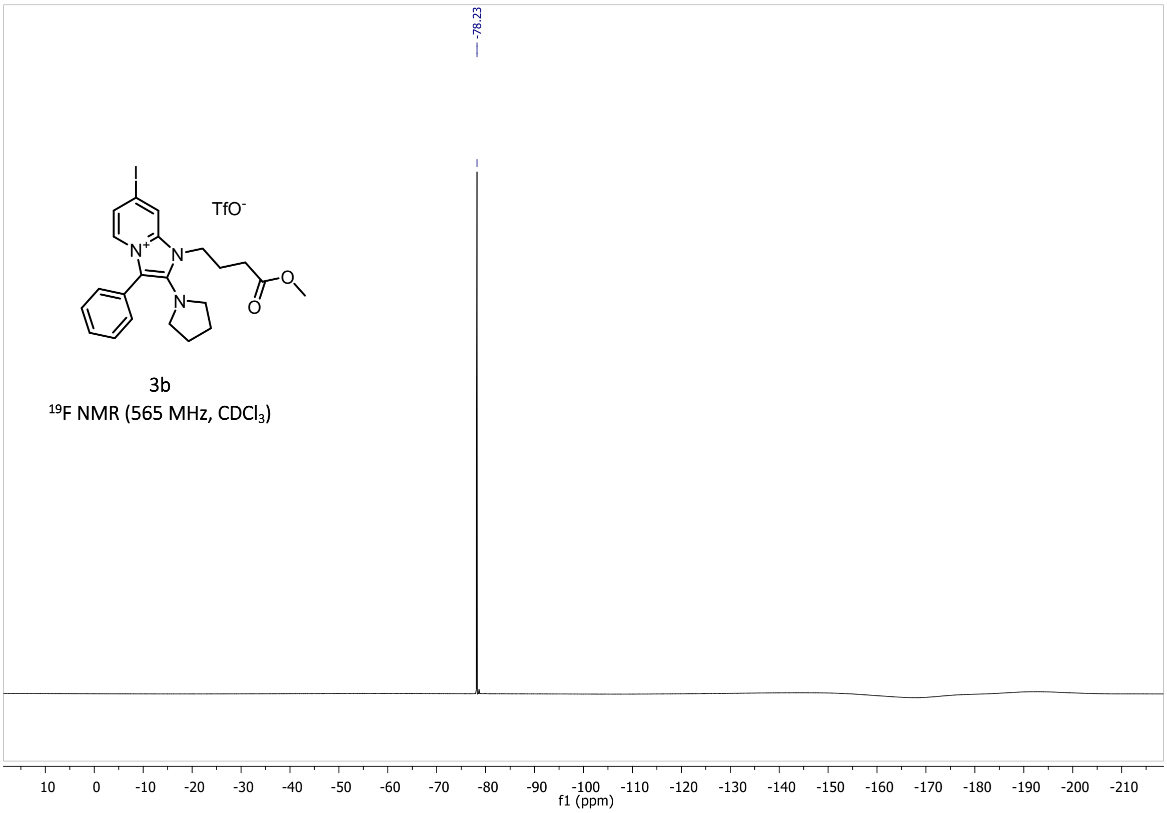
-78.23
TfO-
3b
19F NMR (565 MHz, CDCl3)
f1 (ppm)

7-(4-(diethylamino)phenyl)-1-(7-methoxy-7-oxoheptyl)-3-phenyl-2-(pyrrolidin-1-yl)-1*H*-imidazo[1,2-*a*]pyridin-4-ium trifluoromethanesulfonate (4a)

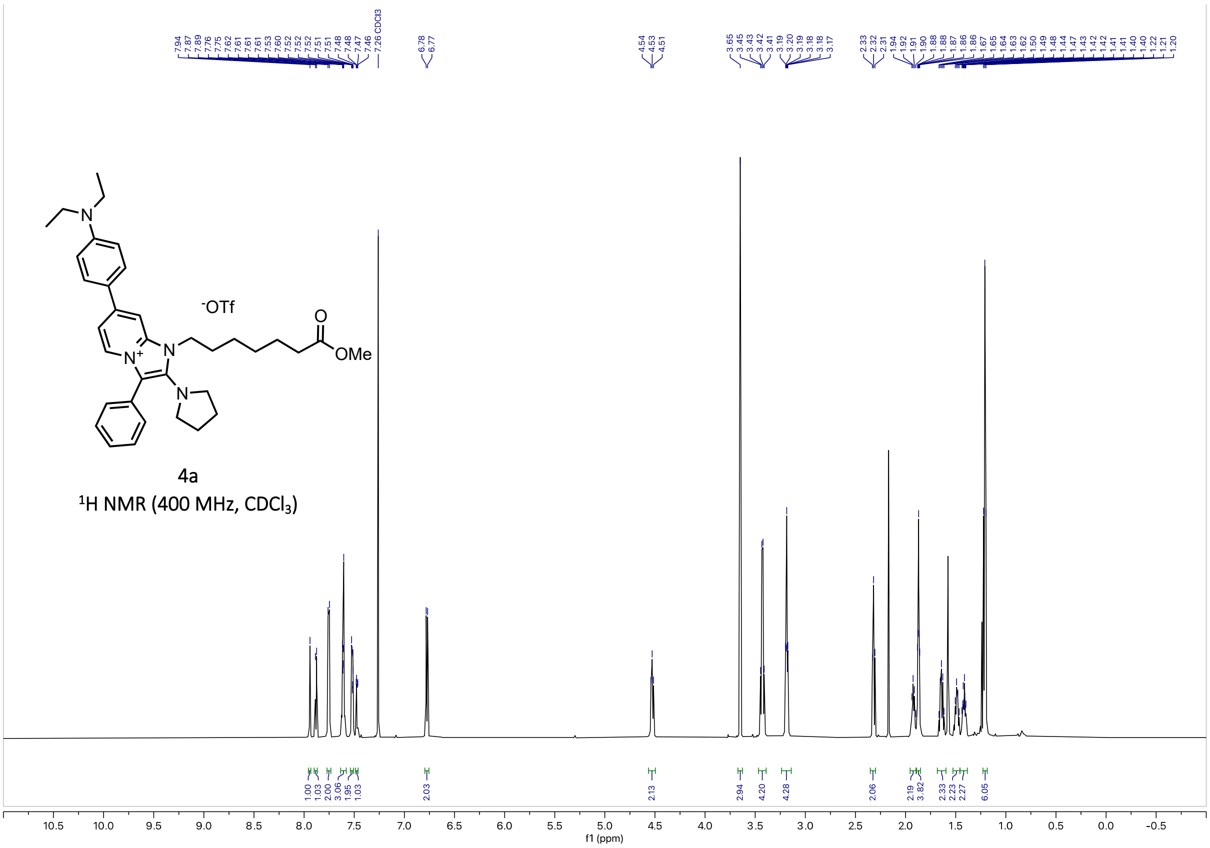

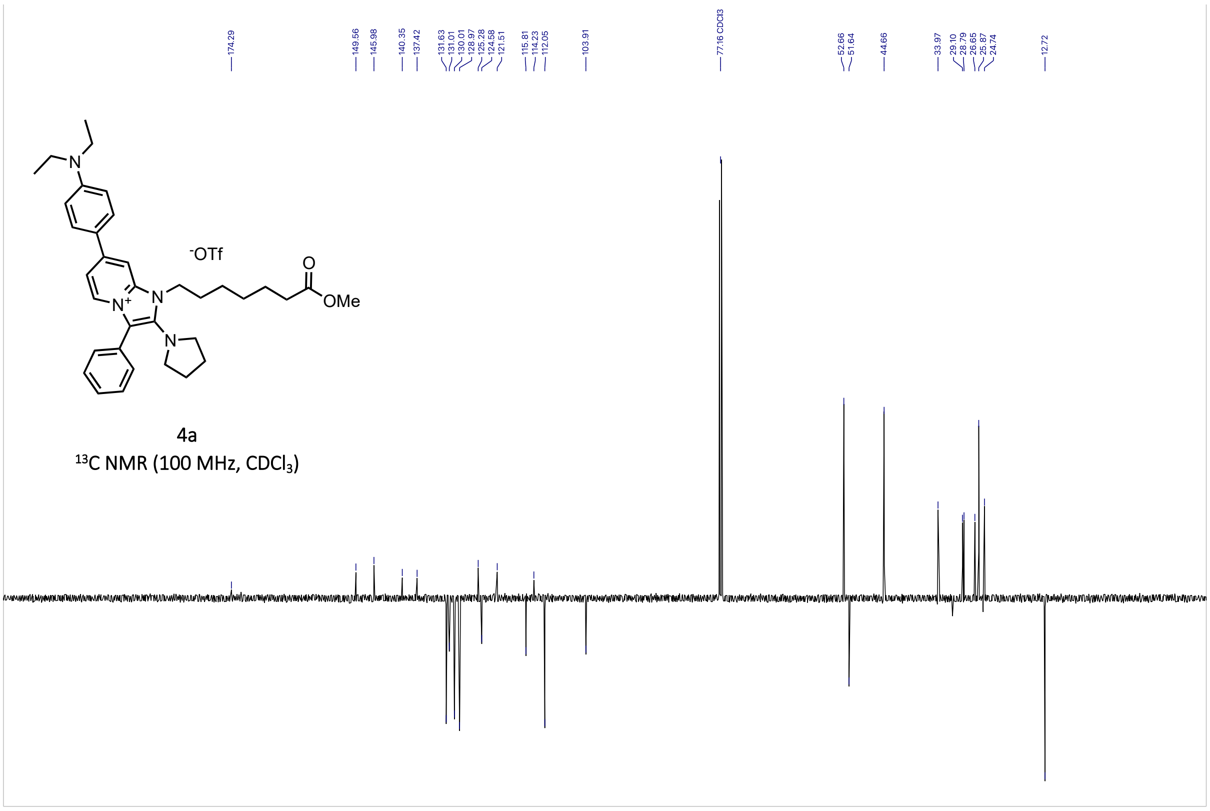

7-(4-(Diethylamino)phenyl)-1-(4-methoxy-4-oxobutyl)-3-phenyl-2-(pyrrolidin-1-yl)-1H-imidazo[1,2-a]pyridin-4-ium trifluoromethanesulfonate (4b)

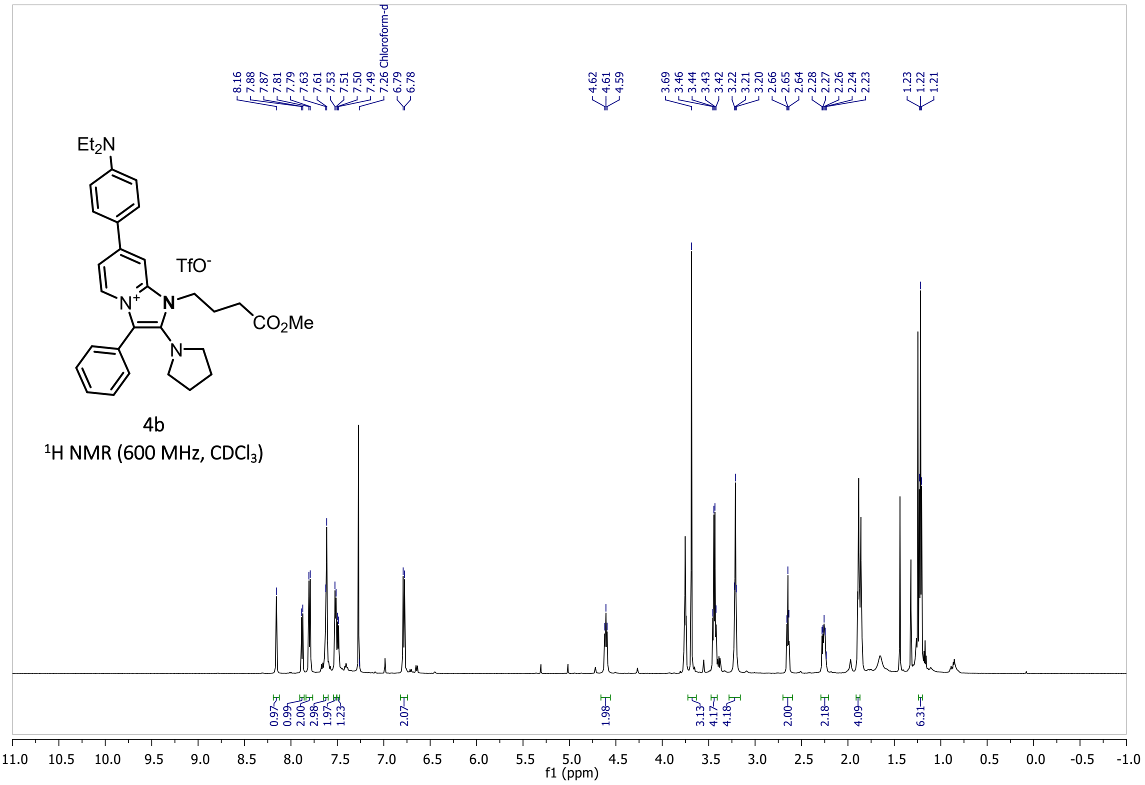

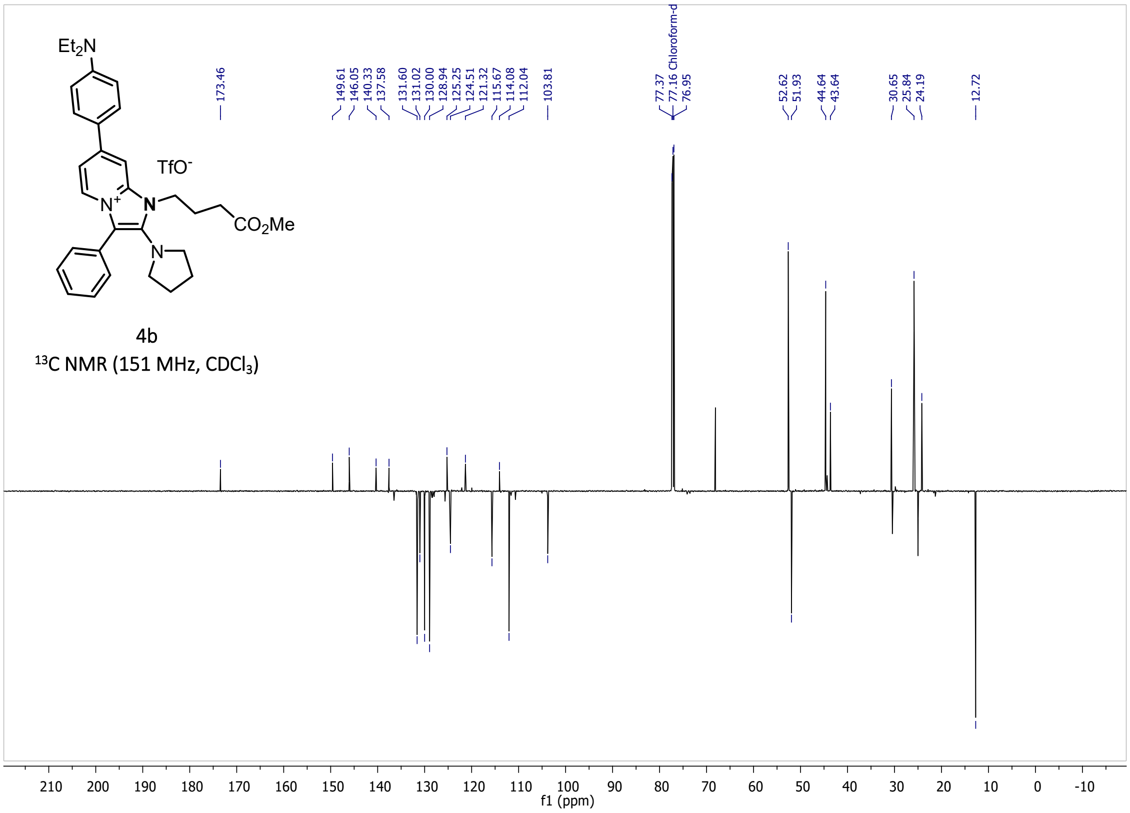

Et_2N
TfO^-
CO_2Me
4b
^{13}C NMR (151 MHz, $CDCl_3$)
173.46
149.61
146.05
140.33
137.58
131.60
131.02
130.00
128.94
125.25
124.51
121.32
115.67
114.08
112.04
103.81
77.37
77.16 Chloroform-d
76.95
52.62
51.93
44.64
43.64
30.65
25.84
24.19
12.72
f1 (ppm)

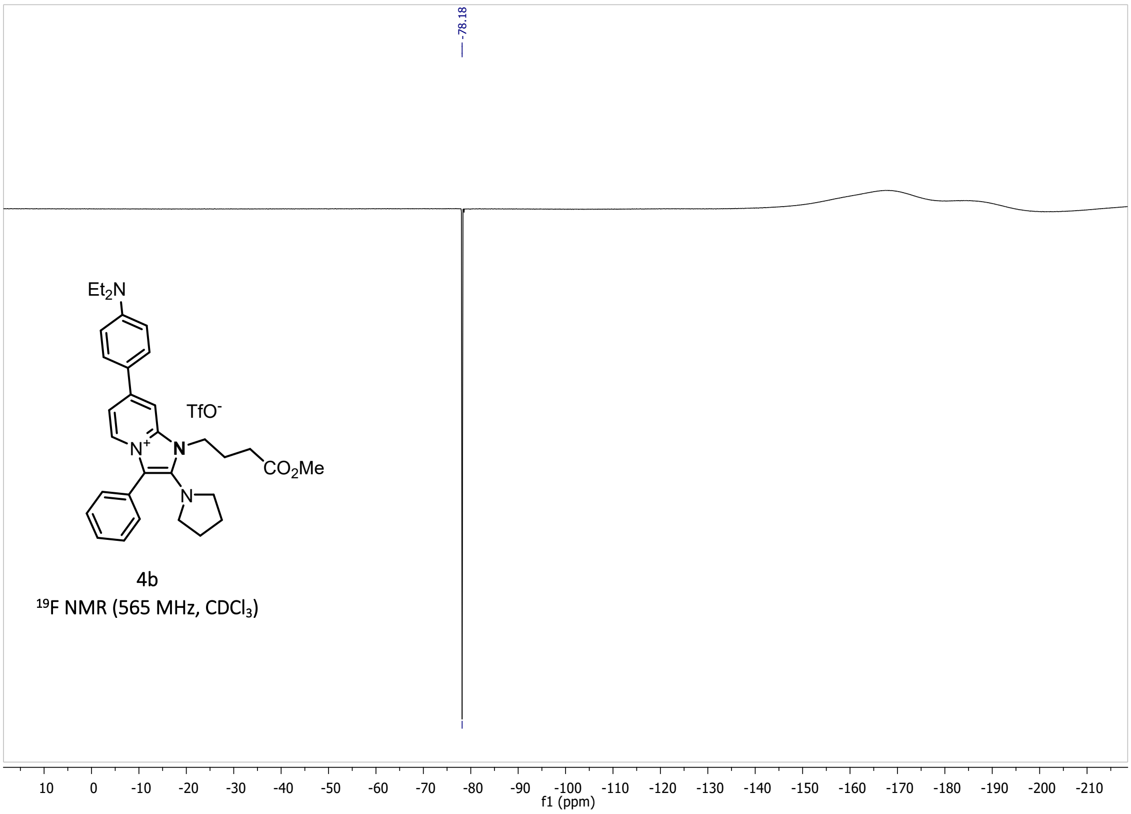

-78.18
Et_2N
TfO^-
CO_2Me
4b
^{19}F NMR (565 MHz, $CDCl_3$)
f1 (ppm)

(*S*)-7-(4-(diethylamino)phenyl)-1-(7-(((1-(3-(dimethylamino)propyl)-1-(4-fluorophenyl)-1,3-dihydroisobenzofuran-5-yl)methyl)amino)-7-oxoheptyl)-3-phenyl-2-(pyrrolidin-1-yl)-1*H*-imidazo[1,2-*a*]pyridin-4-ium trifluoromethanesulfonate (PYR-C6-CIT)

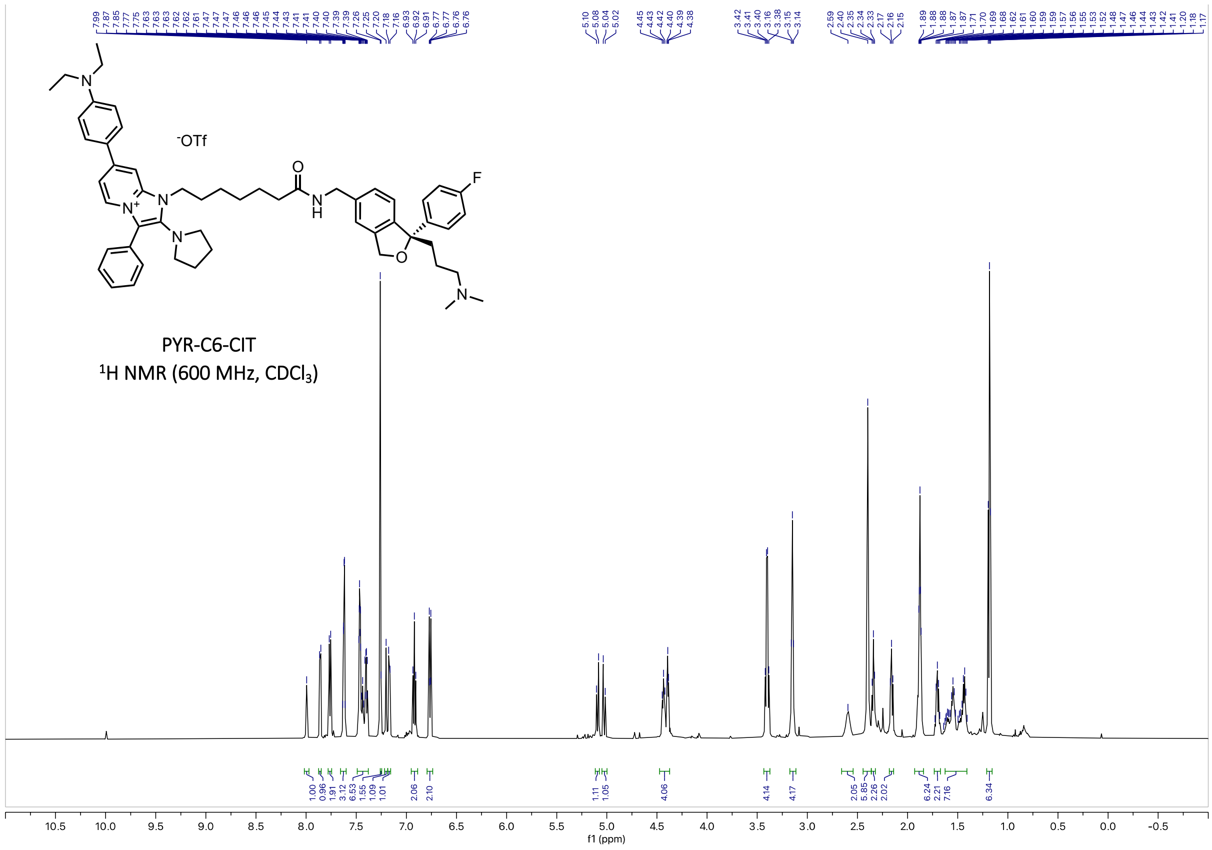

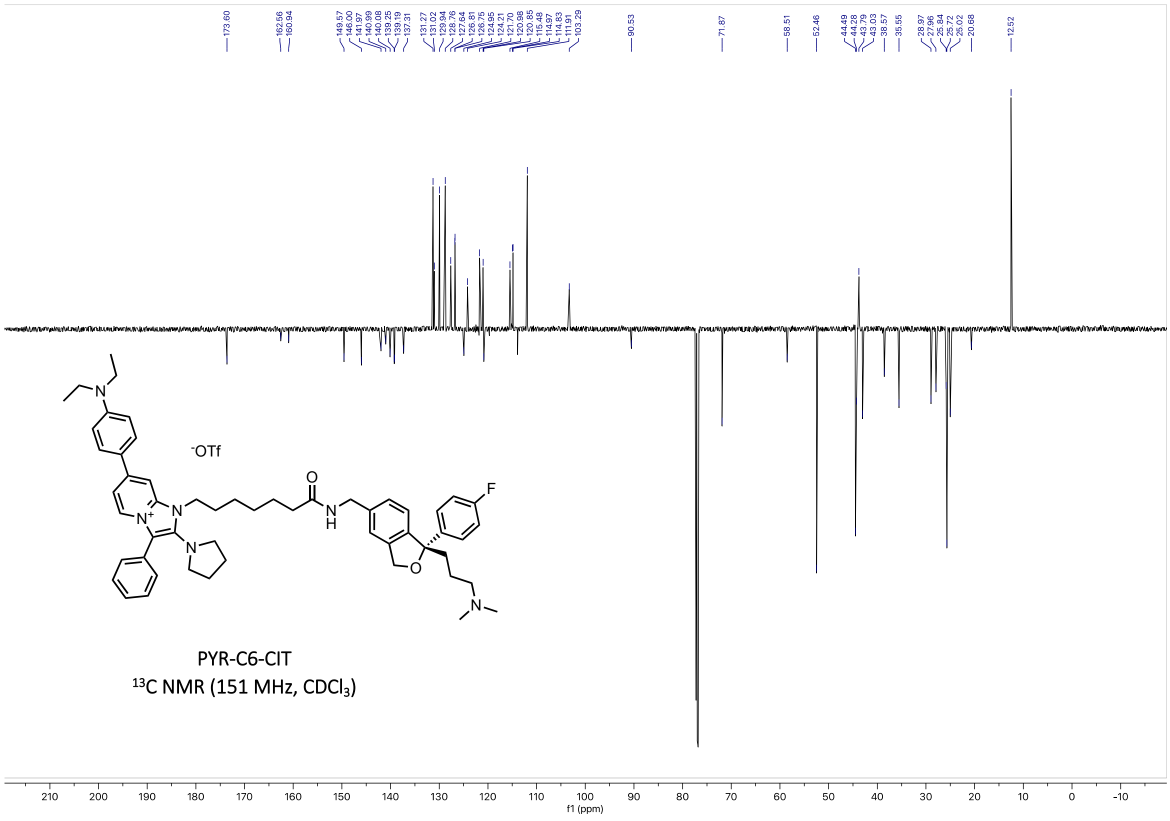

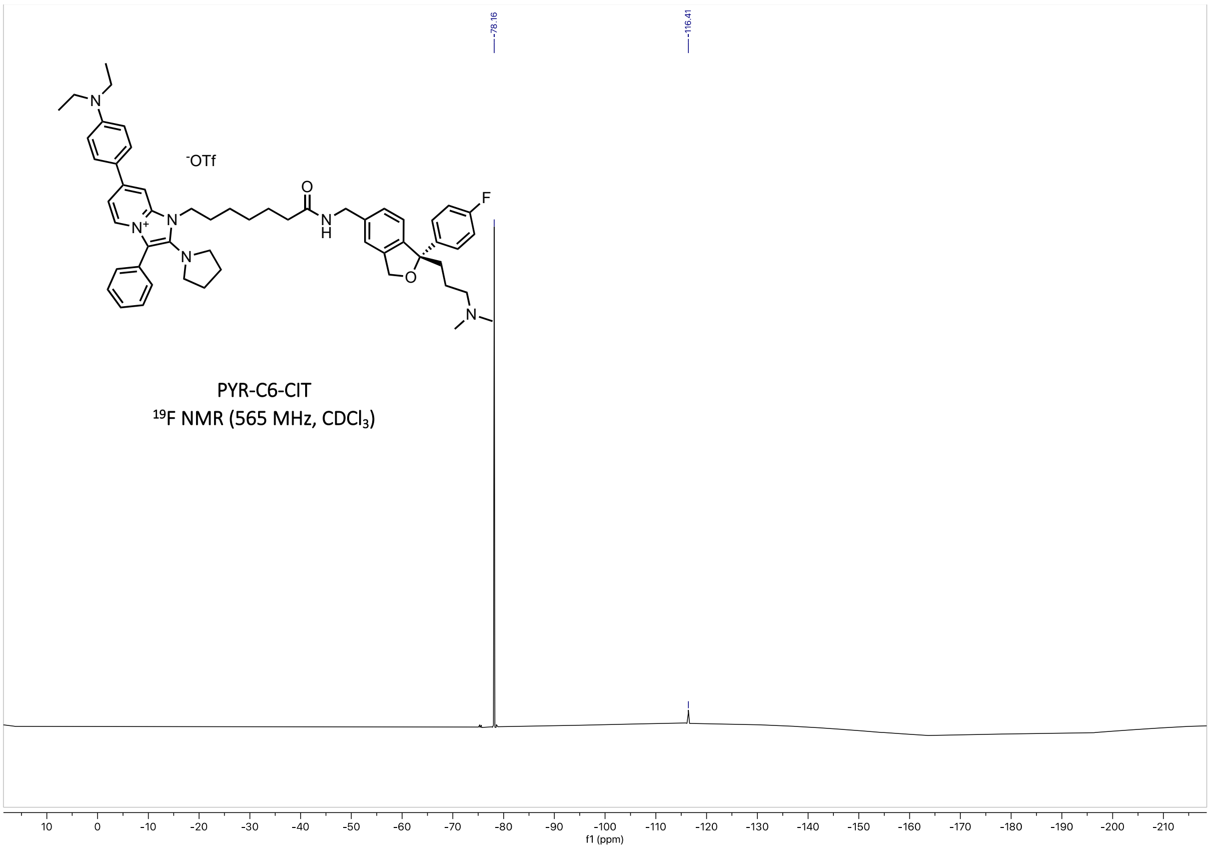

-78.16
-116.41
-OTf
PYR-C6-CIT
19F NMR (565 MHz, CDCl3)
10 0 -10 -20 -30 -40 -50 -60 -70 -80 -90 -100 -110 -120 -130 -140 -150 -160 -170 -180 -190 -200 -210
f1 (ppm)

(*S*)-7-(4-(Diethylamino)phenyl)-1-(4-(((1-(3-(dimethylamino)propyl)-1-(4-fluorophenyl)-1,3-dihydroisobenzofuran-5-yl)methyl)amino)-4-oxobutyl)-3-phenyl-2-(pyrrolidin-1-yl)-1*H*-imidazo[1,2-a]pyridin-4-ium trifluoromethanesulfonate (PYR-C3-CIT)

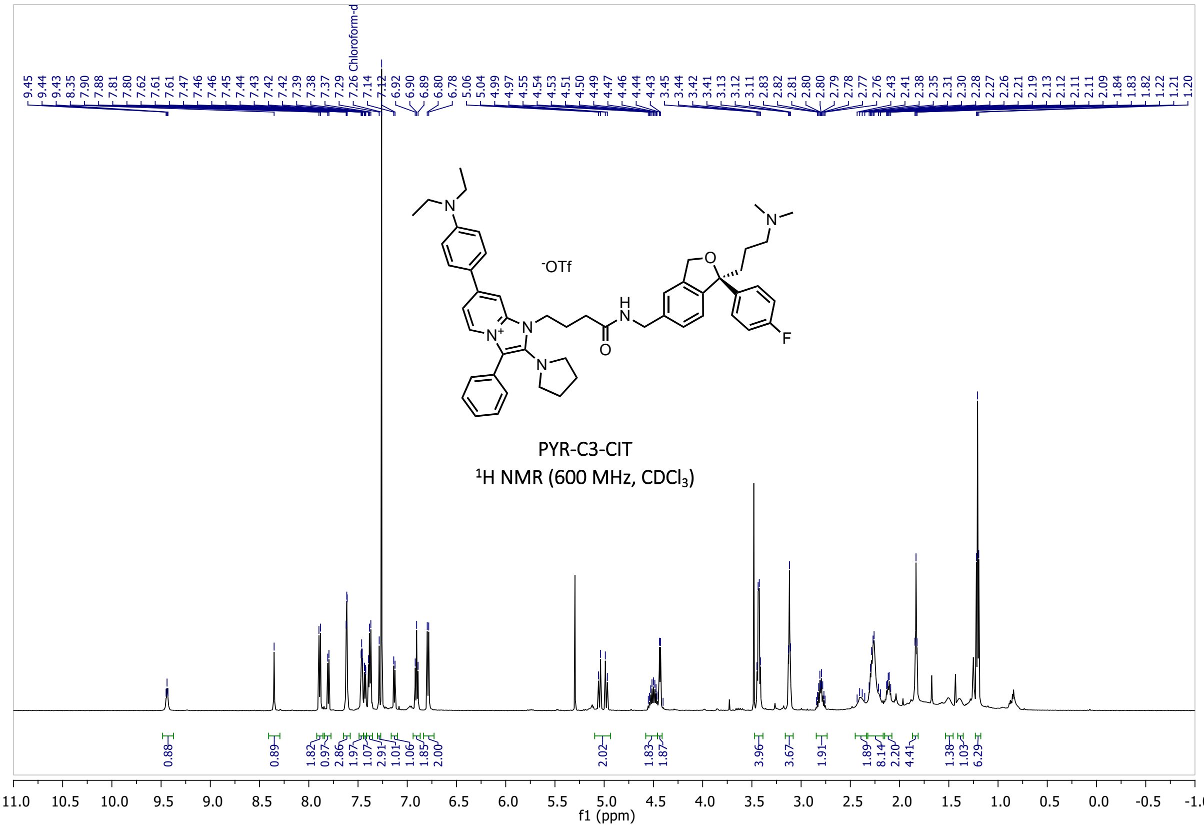

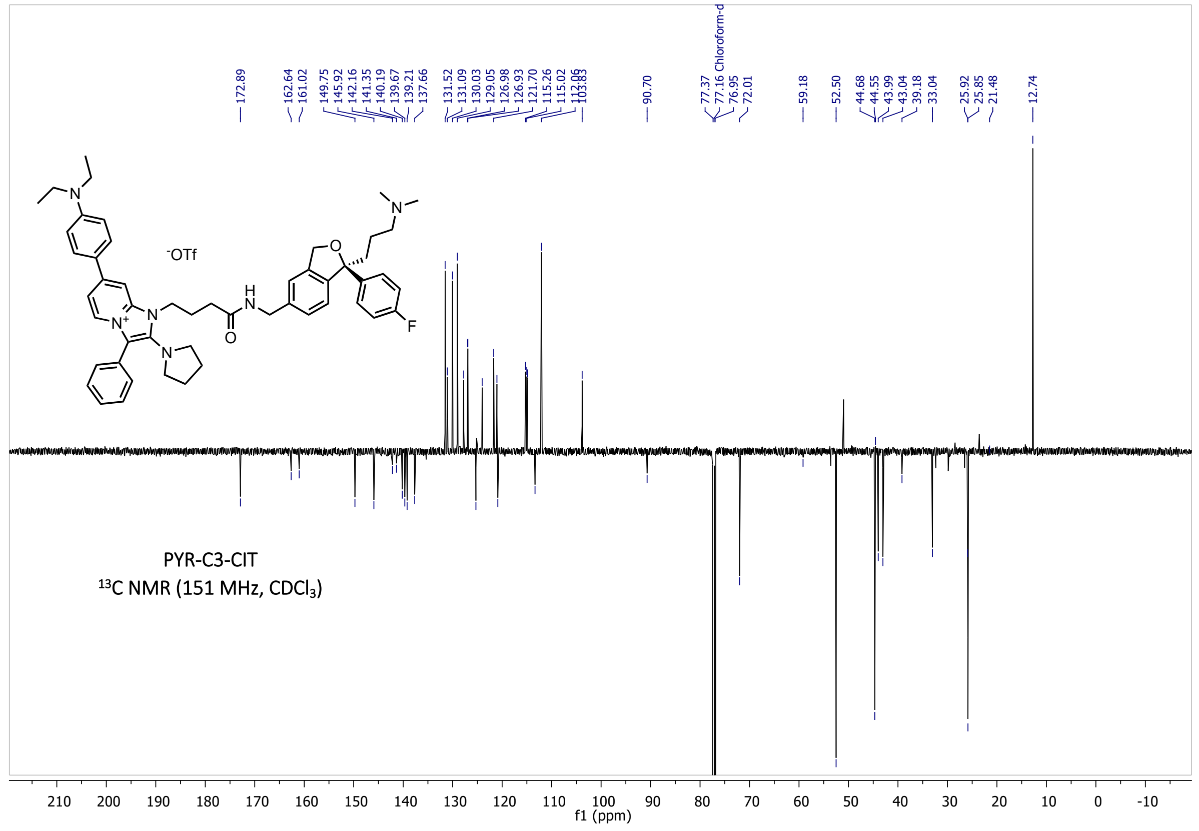

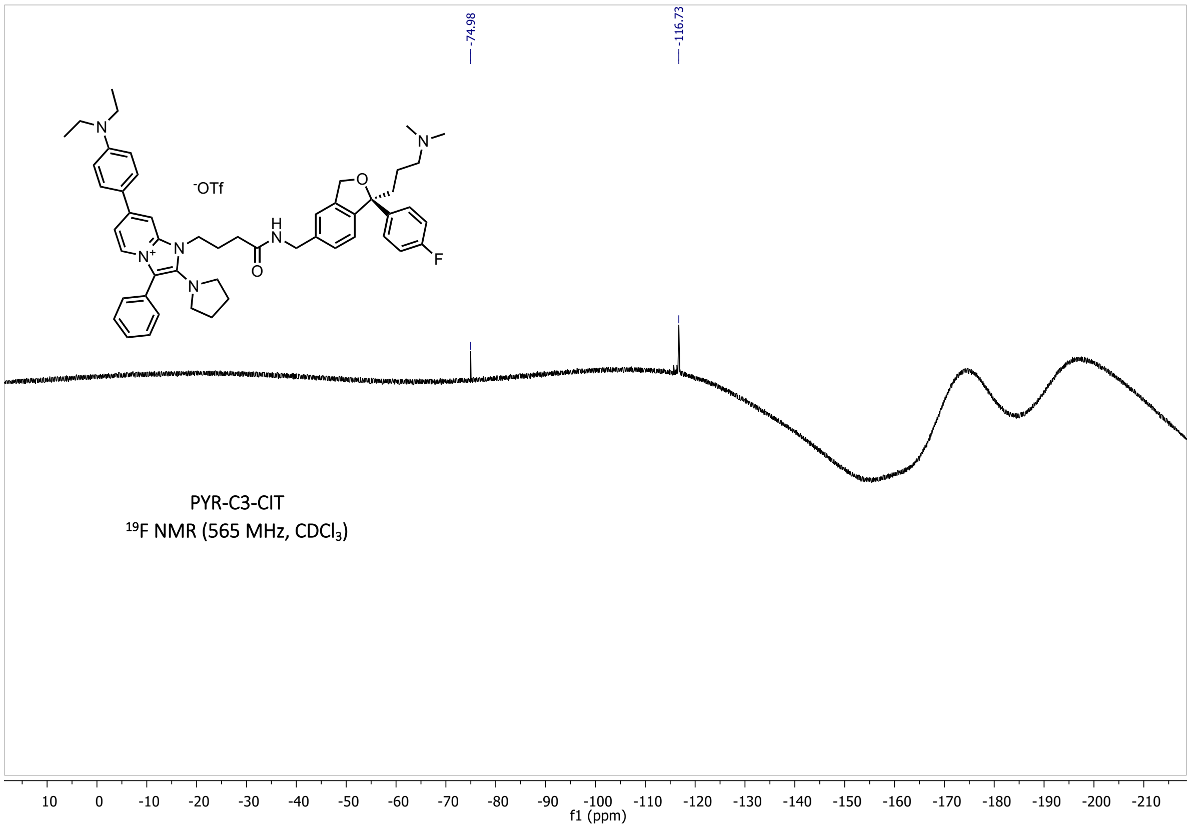

-74.98
-116.73
-OTf
N
H
O
F
PYR-C3-CIT
19F NMR (565 MHz, CDCl3)
10 0 -10 -20 -30 -40 -50 -60 -70 -80 -90 -100 -110 -120 -130 -140 -150 -160 -170 -180 -190 -200 -210
f1 (ppm)

REFERENCES

(1) Bolte, S.; Cordelières, F. P. A Guided Tour into Subcellular Colocalization Analysis in Light Microscopy. *J. Microsc.* **2006**, *224* (3), 213–232. https://doi.org/10.1111/j.1365-2818.2006.01706.x.

(2) Morris, G. M.; Huey, R.; Lindstrom, W.; Sanner, M. F.; Belew, R. K.; Goodsell, D. S.; Olson, A. J. AutoDock4 and AutoDockTools4: Automated Docking with Selective Receptor Flexibility. *J. Comput. Chem.* **2009**, *30* (16), 2785–2791. https://doi.org/10.1002/jcc.21256.

(3) Coleman, J. A.; Green, E. M.; Gouaux, E. X-Ray Structures and Mechanism of the Human Serotonin Transporter. *Nature* **2016**, *532* (7599), 334–339. https://doi.org/10.1038/nature17629.

(4) Case, D. A.; Cerutti, D. S.; Cheatham III, T. E.; Darden, T. A.; Duke, R. E.; Giese, T. J.; Gohlke, H.; Goetz, A. W.; Greene, D.; Homeyer, N.; Izadi, S.; Kovalenko, A.; Lee, T. S.; LeGrand, S.; Li, P.; Lin, C.; Liu, J.; Luchko, T.; Luo, R.; Mermelstein, D.; Merz, K. M.; Monard, G.; Nguyen, H.; Omelyan, I.; Onufriev, A.; Pan, F.; Qi, R.; Roe, D. R.; Roitberg, A.; Sagui, C.; Simmerling, C. L.; Botello-Smith, W. M.; Swails, J.; Walker, R. C.; Wang, J.; Wolf, R. M.; Wu, X.; Xiao, L.; York, D. M.; Kollman, P. A. AMBER 2017. University of California, San Francisco 2017.

(5) Jakalian, A.; Jack, D. B.; Bayly, C. I. Fast, Efficient Generation of High-Quality Atomic Charges. AM1-BCC Model: II. Parameterization and Validation. *J. Comput. Chem.* **2002**, *23* (16), 1623–1641. https://doi.org/10.1002/jcc.10128.

(6) Maier, J. A.; Martinez, C.; Kasavajhala, K.; Wickstrom, L.; Hauser, K. E.; Simmerling, C. Ff14SB: Improving the Accuracy of Protein Side Chain and Backbone Parameters from Ff99SB. *J. Chem. Theory Comput.* **2015**, *11* (8), 3696–3713. https://doi.org/10.1021/acs.jctc.5b00255.

(7) Singer, N. K.; González, L.; Monari, A. Molecular Photoswitches Regulating the Activity of the Human Serotonin Transporter. *J. Phys. Chem. Lett.* **2023**, *14*, 10333–10339. https://doi.org/10.1021/acs.jpclett.3c02655.

(8) Phillips, J. C.; Braun, R.; Wang, W.; Gumbart, J.; Tajkhorshid, E.; Villa, E.; Chipot, C.; Skeel, R. D.; Kalé, L.; Schulten, K. Scalable Molecular Dynamics with NAMD. *J. Comput. Chem.* **2005**, *26* (16), 1781–1802. https://doi.org/10.1002/jcc.20289.

(9) Phillips, J. C.; Hardy, D. J.; Maia, J. D. C.; Stone, J. E.; Ribeiro, J. V.; Bernardi, R. C.; Buch, R.; Fiorin, G.; Hénin, J.; Jiang, W.; McGreevy, R.; Melo, M. C. R.; Radak, B. K.; Skeel, R. D.; Singharoy, A.; Wang, Y.; Roux, B.; Aksimentiev, A.; Luthey-Schulten, Z.; Kalé, L. V.; Schulten, K.; Chipot, C.; Tajkhorshid, E. Scalable Molecular Dynamics on CPU and GPU Architectures with NAMD. *J. Chem. Phys.* **2020**, *153* (044130). https://doi.org/10.1063/5.0014475.

(10) Dickson, C. J.; Madej, B. D.; Skjevik, Å. A.; Betz, R. M.; Teigen, K.; Gould, I. R.; Walker, R. C. Lipid14: The Amber Lipid Force Field. *J. Chem. Theory Comput.* **2014**, *10* (2), 865–879. https://doi.org/10.1021/ct4010307.

(11) Jorgensen, W. L.; Chandrasekhar, J.; Madura, J. D.; Impey, R. W.; Klein, M. L. Comparison of Simple Potential Functions for Simulating Liquid Water. *J. Chem. Phys.* **1983**, *79* (2), 926–935. https://doi.org/10.1063/1.445869.

(12) Li, P.; Roberts, B. P.; Chakravorty, D. K.; Merz, K. M. Rational Design of Particle Mesh Ewald Compatible Lennard-Jones Parameters for +2 Metal Cations in Explicit Solvent. *J. Chem. Theory Comput.* **2013**, *9* (6), 2733–2748. https://doi.org/10.1021/ct400146w.

(13) Hopkins, C. W.; Le Grand, S.; Walker, R. C.; Roitberg, A. E. Long-Time-Step Molecular Dynamics through Hydrogen Mass Repartitioning. *J. Chem. Theory Comput.* **2015**, *11* (4), 1864–1874. https://doi.org/10.1021/ct5010406.

(14) Andersen, H. C. Rattle: A “Velocity” Version of the Shake Algorithm for Molecular Dynamics Calculations. *J. Comput. Phys.* **1983**, *52* (1), 24–34. https://doi.org/10.1016/0021-9991(83)90014-1.

(15) Ryckaert, J. P.; Ciccotti, G.; Berendsen, H. J. C. Numerical Integration of the Cartesian Equations of Motion of a System with Constraints: Molecular Dynamics of n-Alkanes. *J. Comput. Phys.* **1977**, *23* (3), 327–341. https://doi.org/10.1016/0021-9991(77)90098-5.

(16) Humphrey, W.; Dalke, A.; Schulten, K. VMD: Visual Molecular Dynamics. *J. Mol. Graph.* **1996**, *14* (1), 33–38. https://doi.org/10.1016/0263-7855(96)00018-5.

(17) Srinivasan, J.; Cheatham, T. E.; Cieplak, P.; Kollman, P. A.; Case, D. A. Continuum Solvent Studies of the Stability of DNA, RNA, and Phosphoramidate-DNA Helices. *J. Am. Chem. Soc.* **1998**, *120* (37), 9401–9409. https://doi.org/10.1021/ja981844+.

(18) Homeyer, N.; Gohlke, H. Free Energy Calculations by the Molecular Mechanics Poisson-Boltzmann Surface Area Method. *Mol. Inform.* **2012**, *31* (2), 114–122. https://doi.org/10.1002/minf.201100135.

(19) Miller, B. R.; McGee, T. D.; Swails, J. M.; Homeyer, N.; Gohlke, H.; Roitberg, A. E. MMPBSA.Py : An Efficient Program for End-State Free Energy Calculations. *J. Chem. Theory Comput.* **2012**, *8* (9), 3314–3321. https://doi.org/10.1021/ct300418h.

(20) Case, D. A.; Belfon, K.; Ben-Shalom, I. Y.; Brozell, S. R.; Cerutti, D. S.; Cheatham III, T. E.; Cruzeiro, V. W. D.; Darden, T. A.; Duke, R. E.; Giambasu, G.; Gilson, M. K.; Gohlke, H.; Goetz, A. W.; Harris, R.; Izadi, S.; Izmailov, S. A.; Kasavajhala, K.; Kovalenko, A.; Krasny, R.; Kurtzman, T.; Lee, T. S.; LeGrand, S.; Li, P.; Lin, C.; Liu, J.; Luchko, T.; Luo, R.; Man, V.; Merz, K. M.; Miao, Y.; Mikhailovskii, O.; Monard, G.; Nguyen, H.; Onufriev, A.; Pan, F.; Pantano, S.; Qi, R.; Roe, D. R.; Roitberg, A.; Sagui, C.; Schott-Verdugo, S.; Shen, J.; Simmerling, C. L.; Skrynnikov, N. R.; Smith, J.; Swails, J.; Walker, R. C.; Wang, J.; Wilson, L.; Wolf, R. M.; Wu, X.; Xiong, Y.; Xue, Y.; York, D. M.; Kollman, P. A. AMBER 2020. University of California, San Francisco 2020.

(21) Lu, Q.; Luo, R. A Poisson-Boltzmann Dynamics Method with Nonperiodic Boundary Condition. *J. Chem. Phys.* **2003**, *119* (21), 11035–11047. https://doi.org/10.1063/1.1622376.

(22) Marenich, A. V.; Cramer, C. J.; Truhlar, D. G. Universal Solvation Model Based on Solute Electron Density and on a Continuum Model of the Solvent Defined by the Bulk Dielectric Constant and Atomic Surface Tensions. *J. Phys. Chem. B* **2009**, *113* (18), 6378–6396. https://doi.org/10.1021/jp810292n.

(23) Frisch, M. J.; Trucks, G. W.; Schlegel, H. B.; Scuseria, G. E.; Robb, M. A.; Cheeseman, J. R.; Scalmani, G.; Barone, V.; Petersson, G. A.; Nakatsuji, H.; Li, X.; Caricato, M.; Marenich, A. V.; Bloino, J.; Janesko, B. G.; Gomperts, R.; Mennucci, B.; Hratchian, H. P.; Ortiz, J. V.; Izmaylov, A. F.; Sonnenberg, J. L.; Williams-Young, D.; Ding, F.; Lipparini, F.; Egidi, F.; Goings, J.; Peng, B.; Petrone, A.; Henderson, T.; Ranasinghe, D.; Zakrzewski, V. G.; Gao, J.; Rega, N.; Zheng, G.; Liang, W.; Hada, M.; Ehara, M.; Toyota, K.; Fukuda, R.; Hasegawa, J.; Ishida, M.; Nakajima, T.; Honda, Y.; Kitao, O.; Nakai, H.; Vreven, T.; Throssell, K.; Montgomery Jr., J. A.; Peralta, J. E.; Ogliaro, F.; Bearpark, M. J.; Heyd, J. J.; Brothers, E. N.; Kudin, K. N.; Staroverov, V. N.; Keith, T. A.; Kobayashi, R.; Normand, J.; Raghavachari, K.; Rendell, A. P.; Burant, J. C.; Iyengar, S. S.; Tomasi, J.; Cossi, M.; Millam, J. M.; Klene, M.; Adamo, C.; Cammi, R.; Ochterski, J. W.; Martin, R. L.; Morokuma, K.; Farkas, O.; Foresman, J. B.; Fox, D. J. Gaussian 16. Gaussian, Inc., Wallingford CT 2016.

(24) Perdew, J. P. Density-Functional Approximation for the Correlation Energy of the Inhomogeneous Electron Gas. *Phys. Rev. B* **1986**, *33* (12), 8822–8824. https://doi.org/10.1103/PhysRevB.33.8822.

(25) Becke, A. D. Density-Functional Exchange-Energy Approximation with Correct Asymptotic Behavior. *Phys. Rev. A* **1988**, *38* (6), 3098–3100. https://doi.org/10.1103/PhysRevA.38.3098.

(26) Weigend, F.; Ahlrichs, R. Balanced Basis Sets of Split Valence, Triple Zeta Valence and Quadruple Zeta Valence Quality for H to Rn: Design and Assessment of Accuracy. *Phys. Chem. Chem. Phys.* **2005**, *7* (18), 3297–3305. https://doi.org/10.1039/b508541a.

(27) Weigend, F. Accurate Coulomb-Fitting Basis Sets for H to Rn. *Phys. Chem. Chem. Phys.* **2006**, *8* (9), 1057–1065. https://doi.org/10.1039/b515623h.

(28) Grimme, S.; Ehrlich, S.; Goerigk, L. Effect of the Damping Function in Dispersion Corrected Density Functional Theory. *J. Comput. Chem.* **2011**, *32* (7), 1456–1465. https://doi.org/10.1002/jcc.21759.

(29) Becke, A. D. Density-Functional Thermochemistry. III. The Role of Exact Exchange. *J. Chem. Phys.* **1993**, *98* (7), 5648–5652. https://doi.org/10.1063/1.464913.

(30) Neese, F.; Wennmohs, F.; Becker, U.; Riplinger, C. The ORCA Quantum Chemistry Program Package. *J. Chem. Phys.* **2020**, *152* (22), 224108.1-224108.18. https://doi.org/10.1063/5.0004608.

(31) Neese, F. Software Update: The ORCA Program System—Version 5.0. *Wiley Interdiscip. Rev. Comput. Mol. Sci.* **2022**, *12* (5), 1–15. https://doi.org/10.1002/wcms.1606.

(32) Würth, C.; Grabolle, M.; Pauli, J.; Spieles, M.; Resch-Genger, U. Relative and Absolute Determination of Fluorescence Quantum Yields of Transparent Samples. *Nat. Protoc.* **2013**, *8* (8), 1535–1550. https://doi.org/10.1038/nprot.2013.087.

(33) Yu, T. Bin; Bai, J. Z.; Guan, Z. Cycloaddition-Promoted Self-Assembly of a Polymer into Well-Defined β Sheets and Hierarchical Nanofibrils. *Angew. Chemie - Int. Ed.* **2009**, *48* (6), 1097–1101. https://doi.org/10.1002/anie.200805009.
